# Neural Processing of Noise‐Vocoded Speech Under Divided Attention: An fMRI‐Machine Learning Study

**DOI:** 10.1002/hbm.70312

**Published:** 2025-08-07

**Authors:** Han Wang, Rongru Chen, Josef Schlittenlacher, Carolyn McGettigan, Stuart Rosen, Patti Adank

**Affiliations:** ^1^ Clinical Systems Neuroscience Section, Department of Developmental Neurosciences Great Ormond Street Institute of Child Health, University College London London UK; ^2^ Department of Neurosurgery Great Ormond Street Hospital for Children NHS Foundation Trust London UK; ^3^ Department of Speech, Hearing and Phonetic Sciences University College London London UK

**Keywords:** divided attention, dual task, functional magnetic resonance imaging, machine learning, neuroimaging, noise‐vocoded speech

## Abstract

In real‐life interaction, we often need to communicate under challenging conditions, such as when speech is acoustically degraded. This issue is compounded by the fact that our attentional resources are often divided when we simultaneously need to engage in other tasks. The interaction between the perception of degraded speech and simultaneously performing additional cognitive tasks is poorly understood. Here, we combined a dual‐task paradigm with functional magnetic resonance imaging (fMRI) and machine learning to establish the neural network supporting degraded speech perception under divided attention. We presented 25 human participants with noise‐vocoded sentences while they engaged in a concurrent visuomotor recognition task, employing a factorial design that manipulated both speech degradation and task difficulty. Participants listened to eight‐band (easier) and four‐band (more difficult) noise‐vocoded sentences, while the Gabor task featured two difficulty levels, determined by the angular discrepancy of the target. We employed a machine learning algorithm (Extreme Gradient Boosting, XGBoost) to evaluate the set of brain areas that showed activity predicting the difficulty of the speech and dual tasks. The results illustrated intelligibility‐related responses in frontal and cingulate cortices and bilateral insulae induced by divided attention. Machine learning further revealed modality‐general and specific responses to speech and visual inputs, in a set of frontotemporal regions reported for domain‐general cognitive functions such as attentional control, motor function, and performance monitoring. These results suggest that the management of attentional resources during challenging speech perception recruits a bilateral operculo‐frontal network also associated with processing acoustically degraded speech.

## Graphical abstract

We used fMRI and machine learning to examine how the brain processes degraded speech under divided attention. Results revealed a bilateral operculo‐frontal network supporting speech processing under a concurrent task, highlighting modality‐general and modality‐specific mechanisms for attentional control when both speech and visual tasks are challenging.

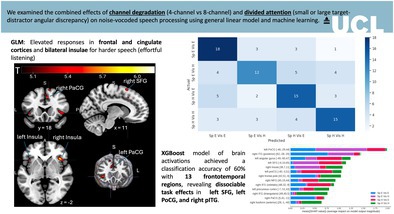

**Summary**

- Listeners recognizing degraded speech under a concurrent task showed increased responses in the frontal/cingulate cortices and insulae, signaling an upregulation of the executive network and effortful listening.
- Concurrent load effects predicted by the responses in these brain regions reflect the dynamic resource dispensing between the two tasks.
- Machine learning provided a robust and explainable approach to revealing the dissociable task effects on activation patterns in a set of frontotemporal regions that were hidden by inferential statistics.

## 1 Introduction

Everyday listening often involves processing acoustically degraded speech (e.g., a poor telephone signal), yet listeners can maintain successful recognition in such suboptimal scenarios (McGettigan et al. 2014; Shannon et al. 1995). However, the cognitive functions (e.g., learning and attention; Carroll 1993) and their underlying neural substrates supporting degraded speech processing remain largely unexplored. In some cases, listeners must process such speech in distraction (e.g., chatting while driving), and recent studies using dual tasks show that degraded speech processing remains robust while attention is split between concurrent tasks (Gennari et al. 2018; Hunter and Pisoni 2018; Wang et al. 2023). Nevertheless, speech perception becomes less accurate for a hard compared to an easy concurrent task (e.g., visual search among different number of distractors; Mattys et al. 2014), suggesting a role of attention in speech processing.

Previous studies used noise vocoding to investigate degraded speech processing in normal hearing listeners. Noise vocoding is an acoustic degradation aiming to simulate the speech processing in cochlear implant users (Davis et al. 2005; Rosen et al. 1999), which removes spectral details while preserving low‐frequency amplitude and temporal information (Shannon et al. 1995). Behaviorally, speech recognition improves logarithmically with the number of frequency bands (Shannon et al. 2004). Neurobiologically, frontal‐temporal regions were found to be related to processing noise‐vocoded speech. Increased blood‐oxygen‐level‐dependent (BOLD) responses were located for less degraded sentences in the left anterior superior temporal sulcus and gyrus (STS and STG; Scott et al. 2006), suggesting their sensitivity to acoustic‐phonetic details. Degradation‐dependent activities were also revealed for frontal regions like the left inferior frontal gyrus (IFG), insulae, anterior cingulate gyrus (ACG), and speech motor regions like the left precentral gyrus. Elevated responses were found in these regions for moderately degraded speech (four to six bands) compared to clear speech (Erb et al. 2013; Hervais‐Adelman et al. 2012), insinuating the top‐down processing under effortful listening to degraded speech (Poldrack et al. 1999).

A few studies have explored the effects of distraction on processing degraded speech. Using a selective attention paradigm (i.e., selecting a fraction of sensory inputs while ignoring distractions; Corbetta et al. 1991), Wild et al. (2012) found increased responses in the left IFG for degraded speech than for clear speech when listeners only attended to and performed the speech task but attempted to ignore concurrent distractors. When performing a distracting task while ignoring speech, STS activity was negatively correlated with the degree of speech degradation. Ritz et al. (2022) further showed that even low‐load visual distractors diminished the BOLD response to even mildly degraded speech in bilateral STG (i.e., 12‐band noise vocoding). These results imply that attention modulates IFG activity under effortful listening, and that distraction disrupts acoustic‐phonetic processing in superior temporal areas. Under selective attention, however, it is unclear whether the neural effect of distractors is due to distraction itself or because speech is task‐irrelevant when attention is exhausted by a distraction task. Gennari et al. (2018) instead used a dual‐task paradigm (to investigate divided attention; Hahn et al. 2008) where listeners split their attention between a syllable recognition task and a concurrent visual task. They revealed elevated responses in the paracingulate (PaCG) and ACG to a more difficult visual task, which was negatively correlated with the suppressed response in STG and the middle temporal gyrus (MTG). These findings revealed the neural signature for allocating attention across tasks.

In summary, past studies have explored the neural signatures associated with the effect of acoustic degradation and distraction on speech processing, but no studies have investigated how degradation and divided attention jointly affect speech processing. Moreover, the brain substrates related to attentional allocation under such conditions remain unexplored. We employed a powerful machine learning (ML) method to capture high‐dimensional and nonlinear neural interactions, enabling a comprehensive investigation of these dual challenges. Our findings revealed modality‐general and specific responses to speech and visual inputs in relevant frontotemporal regions and found neural signatures for the dispensing of resources across tasks within this network.

## 2 Materials and Methods

### 2.1 Participants

Twenty‐five participants (14 females [F] and 11 males [M] between 18 and 30 years of age [Y], mean = 24Y, standard deviation [SD] = 4.6Y) completed the study. All self‐declared to be monolingual British English speakers residing in the United Kingdom at the time of the experiment. All reported no neurological disorders (including dyslexia), normal hearing, and normal or corrected‐to‐normal vision. All participants were recruited via the university's recruitment platform Sona Systems (Sona Systems 2023) and paid at a rate corresponding to £12.50 per hour. The experiment was approved by the Research Ethics Committee of University College London (#0599.001).

### 2.2 Speech Task

The speech task in the dual‐task design used sentences from the Bamford–Kowal–Bench (BKB) corpus (Bench et al. 1979) and Adaptive Sentence List (ASL; Macleod and Summerfield 1990) produced by a female speaker (Table A1). Both sentence sets contain short and highly predictable sentences having simple vocabulary and syntax, for example, “He played with his train” (BKB) and “She brought her camera” (ASL). The recordings were collected in an anechoic chamber at UCL using a Type 4190 microphone on a Brüel & Kjær 2231 sound level meter (sampling at 16 bit and 22.05 kHz), which was connected to a Sony 60ES digital audio tape recorder.

The BKB corpus consists of 336 sentences (each with three to four key words), and the ASL corpus contains 270 sentences (each with three key words). The sentence set was first normalized to the same root mean square amplitude (70 dB; Kennedy‐Higgins et al. 2020) in Praat (version 6.1.42; Boersma 2001) before being processed by a noise vocoder adapted from Rosen et al. (1999) in MATLAB (version R2021a; MathWorks). Six hundred and six sentences (also see Section 3.1 *Practice and Main Runs*) were respectively band‐pass filtered into four and eight logarithmically spaced frequency bands between 50 and 5000 Hz following Greenwood's (1990) frequency‐position function. The frequencies of the lower band edges for the four‐band speech were 50 Hz, 311 Hz, 889 Hz, and 2169 Hz. For eight bands, the lower edges were 50 Hz, 155 Hz, 311 Hz, 544 Hz, 889 Hz, 1404 Hz, 2169 Hz, and 3307 Hz. Each band's amplitude envelope was extracted using half‐wave rectification and a low‐pass filter (cutoff at 300 Hz). This envelope was used to modulate a fragment of white noise, which was then filtered by the same band‐pass filter used to extract the envelope, before all the band outputs were summed together.

### 2.3 Concurrent Visual Task

**FIGURE 1:**
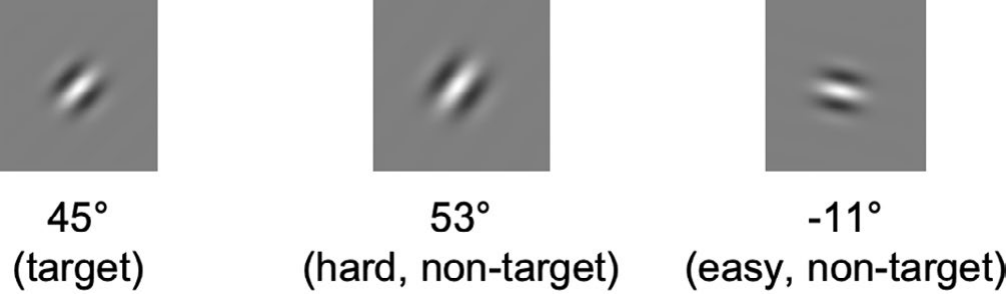
Examples for the target and nontarget Gabor patches used in the visual task. The plots of patches are for illustration purposes thus not scaled to their actual size. These examples do not exhaust all possible orientations of a nontarget patch.

The concurrent task was a visual decision task where participants judged the orientation of a Gabor patch (Calder‐Travis and Ma 2020). Each patch was a sine wave grating presented through a Gaussian window with a SD of 0.16 cm (cm) and a frequency of 2.80 cycles per cm (Figure 1). All stimuli were displayed on a gray background (RGB = [128, 128, 128]). Peaks and troughs of the sine waves took the possible maximum and minimum RGB values ([255, 255, 255] and [0, 0, 0], respectively) at the center of the Gaussian window. We also adjusted the phase of these Gabor patches to ensure there was always a peak of the sine wave at the center of the Gaussian window. Each Gabor patch was located at the center of the display.

## 3 Experimental Design and Statistical Analyses

### 3.1 Practice and Main Runs

**FIGURE 2:**
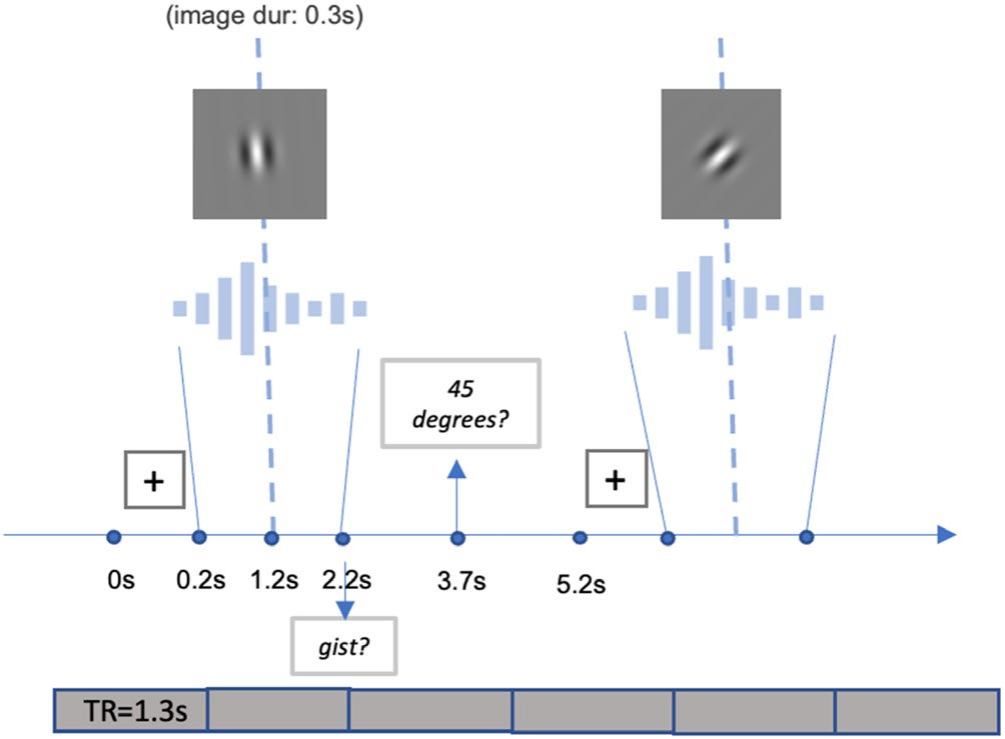
Participants performing the dual task heard an eight‐ or four‐band BKB sentence while they judged the orientation of a Gabor patch presented briefly. They then were prompted to indicate whether they understood the gist of the sentence and whether the Gabor patch was oriented at 45° clockwise from vertical. The plots of the fixation cross and the Gabor patch are for illustration only and not scaled to their actual size. fMRI data acquisition was a continuous scan with a repetition time (i.e., TR) of 1.3 s for each volume (also see Section 3.3).

This study comprised two types of tasks: a single speech task (for practice) and a dual speech‐visual task (for practice and main runs, i.e., sessions). In the dual task, participants recognized a noise‐vocoded sentence while judging whether a Gabor patch was angled at a target orientation (45° clockwise, Figure 1). Participants were not instructed to prioritize either task and were only told to perform both tasks together (Figure 2), as it would be hard to prevent participants from dynamically changing their allocations of resources over time, which might be particularly true for a real‐life scenario. In each trial, a fixation cross was displayed at the screen's center for 200 ms. Participants then heard a noise‐vocoded sentence whose midpoint was aligned to the midpoint of a 2 s window. The Gabor patch appeared 150 ms prior to the midpoint of the sentence duration and ended at 150 ms following the midpoint. Subsequently, participants were given 1.5 s to respond whether they understood the gist of the sentence and another 1.5 s to indicate whether the Gabor patch displayed the target orientation. Participants gave both responses by pressing the left (“Yes”) or right (“No”) arrow keys on a keyboard (for the practice run outside the scanner) or the left (“Yes”) or right (“No”) keys on a button box (for the runs in the scanner, see below). During the single‐task practice, they only heard and responded to the speech stimuli, and each trial terminated after the gist response window.

Speech‐task difficulty was manipulated with the number of frequency bands: the easy task had eight bands, whereas the hard task had four bands. Following Calder‐Travis and Ma (2020) and Wang et al. (2023), visual‐task difficulty was manipulated by varying the difference in orientation between the target and nontarget trials (i.e., target‐distractor angular discrepancy, or TD). The range of difference in orientations between a nontarget and a target (Δ) was 48° < Δ ≤ 60° for the easy task, and 6° < Δ ≤ 18° for the hard task. The orientations of the nontarget Gabor patches came from a uniform distribution, so that all possible nontarget orientations were equally likely to enter the sample.

The study took place at the Birkbeck‐UCL Centre for Neuroimaging. Participants first read the information sheet and signed an informed consent form. They then performed two practice runs (i.e., sessions) outside the scanner. The first run included the single speech task with six trials in each block. The second run was four blocks of the dual task with four trials in each block. For both runs, there was a 12 s rest period between blocks. Task order was pseudo‐randomized between blocks, such that each participant performed each condition once within each run. Stimuli were delivered over the 14‐in. display (1920 × 1080 pixels) of a Lenovo X1 Carbon laptop and Etymotic Research ER4P in‐ear sound monitors.

Participants then went through an functional magnetic resonance imaging (fMRI) safety screening before they were taken into the scanner suite and positioned on the scanner bed. Earphones were inserted (Sensimetrics S14 with Comply P‐Series earbuds), and participants were given a button box for task response. Participants completed one practice run and six main runs in the scanner. The practice run contained four blocks of a dual task with four trials in each block. The main runs had eight blocks of a dual task with six trials within each block. The duration of the rest intervals was jittered within each run, with a mean of 12 s and a range of 10–14 s. Task order was pseudo‐randomized between blocks, such that each participant performed each condition once within the practice run and twice within each main run. Participants were offered a 1‐min break between the runs. Visual stimuli were displayed via a projector onto a 13‐in. screen in the fMRI scanner (1920 × 1080 pixels) and participants viewed the stimuli from around 20 cm from the screen.

For each participant, unique sentences were randomly drawn from the original set of 606 sentences for the practice and main runs. Half of the trials had a correct answer of “Yes” for the visual task within each block. After the main runs conclude, all but one participant (subject 06, for time constraint) had a structural scan before they vacated the scanner suite.

### 3.2 Post‐Scan Task and Questionnaire

Immediately after the main runs, participants performed a surprise memory task aiming to offer a secondary measure for how degraded speech was processed under divided attention as the in‐scanner “gist” report was not a direct measure for the recognition of sentence contents (Wild et al. 2012). In the post‐scan task, participants indicated whether a sentence was presented during the main runs. Ninety‐six extant sentences from the main runs (24 per task condition) and 32 new sentences (randomly drawn from the unused set) comprised the task, where sentence order was randomized. In each trial, a sentence was shown at the center of the display, and participants were prompted to respond: “Did you hear the sentence in the main experiment?” in a 2‐s time window by pressing the left (“Yes”) or right (“No”) arrow keys on a keyboard. The task proceeded to the next trial automatically after the response window. Stimuli were delivered using the laptop used for practice. After the experiment, participants completed a questionnaire where they indicated separately how much effort and attention they invested on a 0–100 scale for each task (see Questionnaire D1 for details).

### 3.3 fMRI Data Acquisition

fMRI scans were performed in a 3‐Tesla PRISMA scanner with gradient‐echo echo‐planar imaging (EPI) using a 32‐channel head coil (TR/TE = 1300 ms/35.20 ms, flip angle = 65°, FOV = 212 × 212 mm, slices = 62, slice thickness = 2 mm, voxel size = 2 × 2 × 2 mm). Volumes were acquired using a multiband sequence in an ascending interleaved order (acceleration factor = 4). Two hundred and sixty volumes were acquired per participant per run. The first four volumes were discarded to allow the T1‐relaxation time to become stable. A structural scan (MPRAGE‐GRAPPA) was acquired using the following parameters: TR/TE = 2300 ms/2.98 ms, flip angle = 9°, FOV = 256 × 256 mm, slices = 208, slice thickness = 2 mm, voxel size = 1 × 1 × 1 mm.

### 3.4 Behavioral Data Analysis

#### 3.4.1 Dual Task

The response for gist understanding in the speech task and the correctness for the visual task were the dependent measures (i.e., 0 or 1). We fit two GLMMs using the mixed() function in the afex R‐package (version 1.3‐0; Singmann et al. 2016) for each task separately to uncover the relationship between the predictors and the behavioral responses in the main runs. The models assumed binomially distributed residuals and adopted a logit link function. Both models had Speech‐Task Difficulty (i.e., easy, hard), Visual‐Task Difficulty (i.e., easy, hard), and their interaction as predictors. Both models initially included random intercepts for Participant and Sentence, and random slopes for Speech‐Task Difficulty, Visual‐Task Difficulty, and their interaction by Participant and Sentence. To select an optimal fitting model for our data, we first removed random effects that caused a convergence failure (Mickan et al. 2020). Next, we excluded the random effects whose inclusion yielded inaccurate estimates of the raw responses—a sign of overfitting (Nannen 2003). Lastly, we applied a backward model selection procedure using the anova() function, which conducted a chi‐square test on the goodness‐of‐fit (i.e., the minus twice the log‐likelihood) of two models. Each time, we performed a comparison between a model and a simpler model excluding a certain random effect and removed the effect from the model where it did not significantly contribute to the model fit. We continued such comparisons until we found the best‐fitting model. The best‐fitting model for speech‐task correctness included random intercepts for Participant and Sentence, as well as random slopes for Speech‐ and Visual‐Task Difficulty by Participant and Speech‐Task Difficulty by Sentence. The final model for visual‐task correctness included a random intercept for Participant and random slopes for Visual‐Task Difficulty by Participant.

To conduct a 2 × 2 factorial analysis, the analysis of variance (ANOVA) tables were generated with the anova() function in the afex package. Here, hypothesis testing on the main effects and interaction was conducted via a chi‐square model comparison on the log likelihood between a simpler model and the model having one more fixed‐effect term in each step. Follow‐up pairwise analyses (whenever a significant interaction term was present) were conducted using the emmeans() and pairs() functions, all under the afex package. The *p*‐value threshold was adjusted by Bonferroni's method for pairwise comparisons. The analysis included data from 23 participants as responses were missing from two participants (subjects 02 and 04) because of a configuration error of the button box.

In the current study, TD was used to modulate the visual‐task performance and to load on speech processing. Visual‐task responses were predicted to be below or near the chance accuracy (50%) to a small TD stimulus as participants might struggle to reject a nontarget due to the small discrepancy. In contrast, for large TD stimuli, responses were predicted to be consistently above chance level as a large TD makes it easy to discern a target (Calder‐Travis and Ma 2020). In addition, speech‐task responses might also be affected by TD, as a performance trade‐off was predicted between the two tasks. As such, two separate GLMM were fitted using the mixed() function in the afex R‐package to further examine how the visual‐task correctness of the nontarget trials (i.e., correct‐rejection rate; main runs) and the speech‐task correctness of these trials varied continuously as a function of TD under different visual‐ and speech‐task difficulties. The model assumed binomially distributed residuals and adopted a logit link function, and had TD (in degrees), Speech‐Task Difficulty (i.e., easy, hard), Visual‐Task Difficulty (i.e., easy, hard), and their interaction as predictors. Both models initially included random intercepts for Participant and Sentence, and random slopes for TD, Speech‐Task Difficulty, Visual‐Task Difficulty, and their interaction by Participant and Sentence. The model selection procedure followed that of the main behavioral analysis outlined above. The final model for visual‐task correctness (nontarget trials) included a random intercept for Participant and random slopes for Visual‐Task Difficulty by Participant. The best model for speech‐task accuracy (of the nontarget trials in the visual task) had a random intercept for Participant and random slopes for Speech‐Task Difficulty by Participant.

#### 3.4.2 Post‐Scan Task

Responses in the post‐scan task were analyzed using signal detection theory by subtracting the *z*‐score of the proportion of hits from the *z*‐score of the proportion of false alarm, giving a measure for the sensitivity of detecting sentences used for the main runs from the unheard sentences (i.e., *d*′; Macmillan 1993). The data was analyzed by a repeated‐measure ANOVA in the afex R‐package. The model included Speech‐Task Difficulty, Visual‐Task Difficulty, and their interaction as a predictor and Participant Rating as a response. The methods for the post hoc analysis (where applicable) followed that of the dual‐task data. The analysis included data from 24 participants as one participant (subject 01) did not perform the task due to the time constraint of the scanning slot.

### 3.5 fMRI Data Analysis

#### 3.5.1 General Linear Model

The imaging data were first checked for abnormalities and converted from DICOM to NIFTI format using the dicom2nifti package (version 1.0.20230411; Li et al. 2016). Subsequent analyses were conducted with Statistical Parametric Mapping 12 (SPM, version 7771; Wellcome Centre for Human Neuroimaging, University College London) using a customized MATLAB script. First, the first functional EPI volume in each run was realigned to the first volume of the first run, before each image in each run was registered to the first volume of that run. The functional volumes in each run were then co‐registered onto the structural scan using linear transformation after manually defining the individual's anterior commissure as the reference point (i.e., [0, 0, 0]) on the structural image. For participants who had a structural scan, a deformation field was generated to probabilistically map the voxels of different tissue types in individual structural images to the tissues of the standard Montreal Neurological Institute 152 (MNI‐152) template space. That deformation field was then used to respectively transform the co‐registered structural and functional images for each run into the MNI space for subsequent group‐level analysis. For the single participant who did not receive a structural scan, their functional images for each run were transformed into the MNI space using the transformation parameters estimated for the mapping between the mean functional image for each run and the MNI template. Functional volumes were smoothed using a 6 × 6 × 6 mm full‐width‐half‐maximum Gaussian kernel.

A model predicting what a canonical BOLD response for the current experiment should look like was created by convolving the experimental design (i.e., a continuous uniform function involving the onset and duration of an experimental block) with a canonical hemodynamic response function (i.e., HRF; Lindquist et al. 2009). Signals at 1/128 Hz or lower were removed from the preprocessed time series of functional images to account for the low‐frequency physical noise of the scanner. Next, GLMs predicting the BOLD responses of the preprocessed functional images were fitted per participant, including the following predictors: (1) the canonical BOLD response (i.e., predictor‐of‐interest; contrast vectors: speech easy visual easy (Sp E Vis E) > rest [1 0 0 0], speech easy visual hard (Sp E Vis H) > rest [0 1 0 0], speech hard visual easy (Sp H Vis E) > rest [0 0 1 0], speech hard visual hard (Sp H Vis H) > rest [0 0 0 1]; the contrast weights were replicated and scaled across runs); (2) the temporal derivative of the canonical BOLD response (to account for temporal delays in the acquisition slices of the hemodynamic responses over a whole volume); (3) the transformation parameters during image realignment (to account for the effect of head motion).

A GLM was fit to examine the effect of speech‐ and visual‐task difficulties on the voxel‐wise, whole‐brain BOLD responses at a group level. We extracted and analyzed the *β* coefficients for the canonical BOLD term from the individual GLMs. Due to data loss (for subjects 01, 02, and 10), the analysis included 22 participants. The model had Speech‐Task Difficulty (i.e., easy, hard), Visual‐Task Difficulty (i.e., easy, hard), and their interaction as predictors. For each contrast (e.g., speech hard > speech easy), voxels were considered significant at a threshold corresponding to a false positive rate of 5% (*p* < 0.05, family‐wise error corrected).

#### 3.5.2 Correlation Analysis

To establish the correlations between brain activity and behavioral effects of task difficulty, we used SPM to extract the mean *β* coefficients in those significant clusters identified by the whole‐brain group analysis for the canonical BOLD term per condition per individual GLM. These values were then averaged across task conditions and correlated with participants' self‐reported effort and attention measures, as well as their mean behavioral task response. Moreover, the difference in BOLD responses between the hard and easy task conditions was correlated with the difference in behavioral task responses of these conditions.

#### 3.5.3 ML

##### 3.5.3.1 Extreme Gradient Boosting (XGBoost)

The traditional voxel‐based‐mass‐univariate approach using GLM is often biased toward focal response magnitude (i.e., *β* coefficients) between the corresponding voxels and can only detect the linear contribution of task conditions in predicting BOLD responses. Moreover, in hypothesis testing using GLM, the interpretation of results is usually constrained by a conventional statistical significance threshold. For example, a conventional nonsignificant result cannot validate a null hypothesis as it only indicates a failure to reject it. These issues of GLM make it difficult to find spatially distributed, subtle, and nonlinear differences in activation patterns across conditions (Davatzikos 2004). An alternative is analyzing the data in a multivariate approach, where an ML algorithm in a classification task seeks the relationship between input features X (e.g., the neural activity in different brain regions) and an output Y (e.g., discrete class values like the task conditions). Herein, the algorithm learns to find a separating plane in the multi‐dimensional data space that divides the data into different classes (Kotsiantis et al. 2006). Instead of being limited to comparing the cross‐condition response magnitude of a specific voxel or region, this approach therefore allows us to identify information in the patterns of neural responses across brain regions (Weaverdyck et al. 2020).

XGBoost (Chen and Guestrin 2016) was used to predict task conditions from neural response patterns and identify brain regions that impact most on classification. Gradient boosting is a model ensemble technique where multiple weak learning algorithms (e.g., simple classification trees) are sequentially trained and added to create a strong learner. After initiating the model to generate a prediction, the algorithm fits the model iteratively on the residual of the previous model and adds the predicted residual to the old prediction. Compared to traditional gradient boosting, XGBoost controls overfitting by penalizing the complexity of weak learners, for example, via shrinking individual trees' leaf weights for a large sum of residuals (i.e., a more complex model; Chen and Guestrin 2016; also see Equation (4) in Note A3).

XGBoost was chosen for the following reasons: (1) the algorithm has achieved state‐of‐the‐art performance in many data mining competitions with scores outperforming more complex models like deep neural networks (Chen and Guestrin 2016); (2) the algorithm is useful for learning nonlinear relationships because it holds no assumption about the relationships between variables compared to other methods like support vector machine, one of the most widely used algorithms for fMRI multivariate analysis (Chen and Guestrin 2016; Salcedo‐Sanz et al. 2014); and (3) tree‐based models show robust performance even with small to medium datasets (Floares et al. 2017; Ghosh et al. 2017). XGBoost has been recently applied for diagnostic analysis using both MRI (Ryu et al. 2020; Wang et al. 2018) and fMRI images (Pang et al. 2021; Torlay et al. 2017): Across these four XGBoost imaging studies, sample sizes were in the range of 16–142 per category, and two to four categories were used for classification. Thus, the sample size per class (*N* = 25) seems to be rather small in the current study. Nevertheless, XGBoost has shown exceptional accuracy in recognizing a small sample of patients with epilepsy (*N* = 16) from healthy participants (*N* = 39) based on the analysis of their language networks using fMRI images acquired under a phonological and a semantic task (Torlay et al. 2017). Therefore, XGBoost seems to be a viable and robust tool for multivariate analysis of fMRI activation patterns with a small to medium sample size.

##### 3.5.3.2 Model Training and Evaluation

One hundred unthresholded *t*‐value statistical parametric maps (SPM t‐maps) from individual GLMs were used as the input feature matrix X to XGBoost to predict an output vector Y (i.e., task conditions). T‐maps, instead of the raw preprocessed images were used, as they were also the input to the group‐level GLM analysis, allowing a direct comparison between the two approaches. There were 25 t‐maps per condition, with parameters scaled across the six functional runs. Labels of task conditions were encoded as numeric values for the response vector Y. To avoid overfitting, the dimensions of the input t‐maps (i.e., 79 × 95 × 79) were reduced by averaging the voxels per larger region of the cortex, according to brain parcellation proposed by Schaefer et al. (2018) based on resting‐state functional connectivity. Learning algorithms (see Note A3 for details) were implemented with the XGBoost (version 1.7.6; Chen and Guestrin 2016) and scikit‐learn (version 1.2.2; Pedregosa et al. 2011) Python packages on an Nvidia V100 Tensor Core GPU (Python version 3.10).

Hyperparameters (see Table 1) and parcellation resolutions (i.e., numbers of features: 100, 200, 300, 400, and 500 parcels) were tested in an exhaustive grid search to find a baseline (whole brain) model with the parameters yielding the best validation accuracy. Here, the model was evaluated in a stratified five‐fold cross validation. For each combination of the hyperparameters, the data was split into five parts, with each part having a balanced representation of class labels. Then, the model was trained and tested five times, each time using a different fold as the test set and the rest as the training set. The mean validation accuracy per hyperparameter set was used as a metric for model performance. A held‐out test set was not included considering the relatively small sample size, following previous imaging studies using XGBoost (Pang et al. 2021; Ryu et al. 2020; Torlay et al. 2017; Wang et al. 2018). After finding the best hyperparameters (Table 1), a binomial test was conducted to determine whether the observed model accuracy was due to chance by comparing the best accuracy against a binomial distribution of 5760 configurations (see Table 1) having a sample size of 100 and a probability of success of 0.25. The distribution generation and the binomial test were respectively conducted using the rbinom() and pbinom() functions in the stats R‐package (version 4.3.2).

**TABLE 1:** Hyperparameters tested for the baseline XGBoost model.

Hyperparameters	Description	Tested values	Best value
colsample_bytree	The proportion of features to be randomly sampled per tree	0.1, 0.3, 0.5, 0.7	0.1
gamma	The minimum loss reduction required to make a tree split	0, 1, 2, 3, 4, 5, 6, 7	0
learning_rate	Learning rate	0.01, 0.04, 0.07, 0.1, 0.13	0.07
max_depth	The maximum depth of a tree	1, 2, 3, 4, 5, 6	3
n_estimators	Number of trees	50, 100, 150, 200, 250, 300	100

*Note:* Tested values denote the values tested in an extensive grid search. Best value denotes the values corresponding to the highest validation accuracy.

##### 3.5.3.3 Feature Selection and Model Interpretation

To select the most important features (i.e., brain regions) contributing to model prediction of task conditions, feature importance was evaluated by Shapley Additive Explanations (SHAP; Lundberg and Lee 2017; Štrumbelj and Kononenko 2014). SHAP is based on game theory and the concept of Shapley values (Shapley 1953), which in the ML context explains individual feature value's contribution to the prediction by offering interpretations at individual datapoint level regarding how a feature value drives specific prediction. For a class k, a positive Shapley value for a specific feature suggests that the presence of the feature increases the model's prediction toward that class, whereas a negative Shapley value for a feature indicates that the presence of that feature decreases the model's prediction for that class (see Note A4 for computational details).

Feature selection was performed by ranking the features by their Shapley values and training models iteratively with the best hyperparameters and number of features found for the baseline model (Kha et al. 2021). Shapley values were obtained with the TreeExplainer in the shap Python package (version 0.43; Lundberg et al. 2020). The iteration started with the model containing the top one feature. On each iteration, the immediately lower ranked feature was added to the model. If no improvements were observed for validation accuracy in five consecutive iterations, features corresponding to the latest peak accuracy over the past iterations were selected as the “best” feature set. A grid search was conducted with the selected features to further determine the best hyperparameters for this feature set (see Table B1).

Shapley values in multiclass classification only reflect how changes in feature value impact predicting one class against all other classes, making inference for pairwise classes difficult. Therefore, binary classification models with the selected features were fitted for data from pairs of classes in a grid search for hyperparameters to uncover distinct regions sensitive to speech‐ and/or visual‐task difficulties (i.e., Sp H Vis E and Sp E Vis E, Sp H Vis H and Sp E Vis H, Sp E Vis H and Sp E Vis E, and Sp H Vis H and Sp H Vis E). A binomial test was conducted to determine whether the observed accuracies were due to chance by comparing the best accuracy against a binomial distribution of 5760 configurations (see Table B3) having a sample size of 100 and a probability of success of 0.5 (the *p*‐value threshold was adjusted by Bonferroni's method for four tests).

## 4 Results

### 4.1 Behavioral Results

#### 4.1.1 Speech Task

**FIGURE 3:**
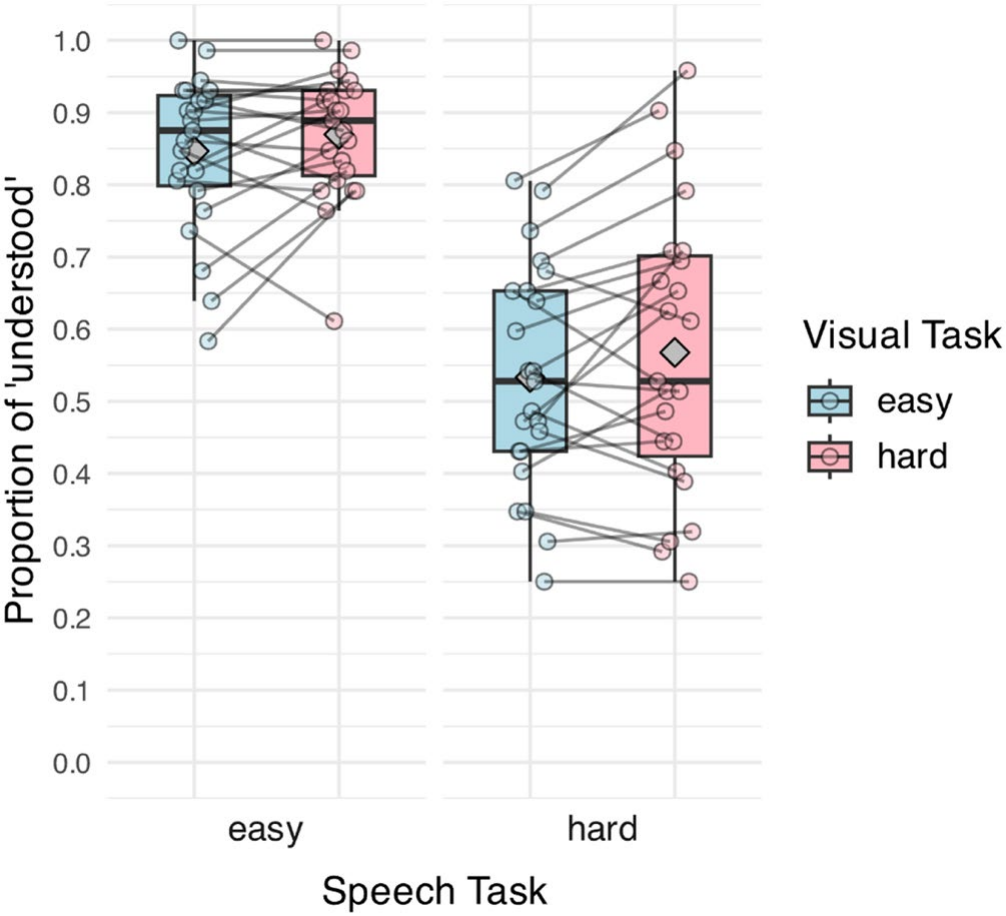
Proportion of sentence reported as “understood” for the speech task. The fill of the boxes represents visual‐task difficulty. *X*‐axis shows speech‐task difficulty. Points display the mean response per participant, and lines connect the individual responses across conditions. Gray diamonds denote the mean of each task condition.

Table 2 shows the fixed‐effect outputs for the GLMM. Figure 3 illustrates the proportion of sentences reported as “understood” by speech‐ and visual‐task difficulty. The main effects of speech‐ and visual‐task difficulty on sentence report were significant, but there was no interaction between the two predictors. The difficulty of the two tasks modulated speech recognition performance in opposite directions. Listeners understood fewer sentences for hard than for easy speech, but they understood more sentences when the visual task was hard than when it was easy (Sp E Vis E: 0.857 [SD = 0.11]; Sp H Vis E: 0.533 [SD = 0.15]; Sp E Vis H: 0.870 [SD = 0.09]; Sp H Vis H: 0.568 [SD = 0.20]).

**TABLE 2:** Model outputs for the GLMM assessing the fixed effects of Speech‐ and Visual‐task difficulty on the proportion of “understood” for the speech task.

	Df	Chisq	Chi Df	*p*
DiffSpeech	12	59.91	1	**< 0.001**
DiffVisual	12	5.07	1	**0.024**
DiffSpeech:DiffVisual	12	0.82	1	**0.366**

*Note:* Bold values indicate statistically significant values.

Abbreviations: Chi Df: the degrees of freedom associated with the chi‐squared distribution; Chisq: the fixed‐effect term's chi‐squared statistics (i.e., difference between the minus twice of the log likelihoods of the two models differing in that fixed‐effect term); Df: degrees of freedom of the fixed‐effect terms; DiffSpeech: Speech‐task difficulty; DiffVisual: Visual‐task difficulty.

In the post‐scan recall task, there was a significant main effect of speech‐task difficulty on the *d*′ score, but the main effect of visual‐task difficulty and the interaction term were not significant. That is, listeners recalled more sentences from the easy‐speech blocks than they did for the hard blocks—Sp E Vis E 0.400 [SD = 0.38]; Sp H Vis E: 0.204 [SD = 0.40]; Sp E Vis H: 0.398 [SD = 0.36]; Sp H Vis H: 0.281 [SD = 0.39] (see Table B2 and Figure C1 for model outputs and visualized results).

#### 4.1.2 Visual Task

**FIGURE 4:**
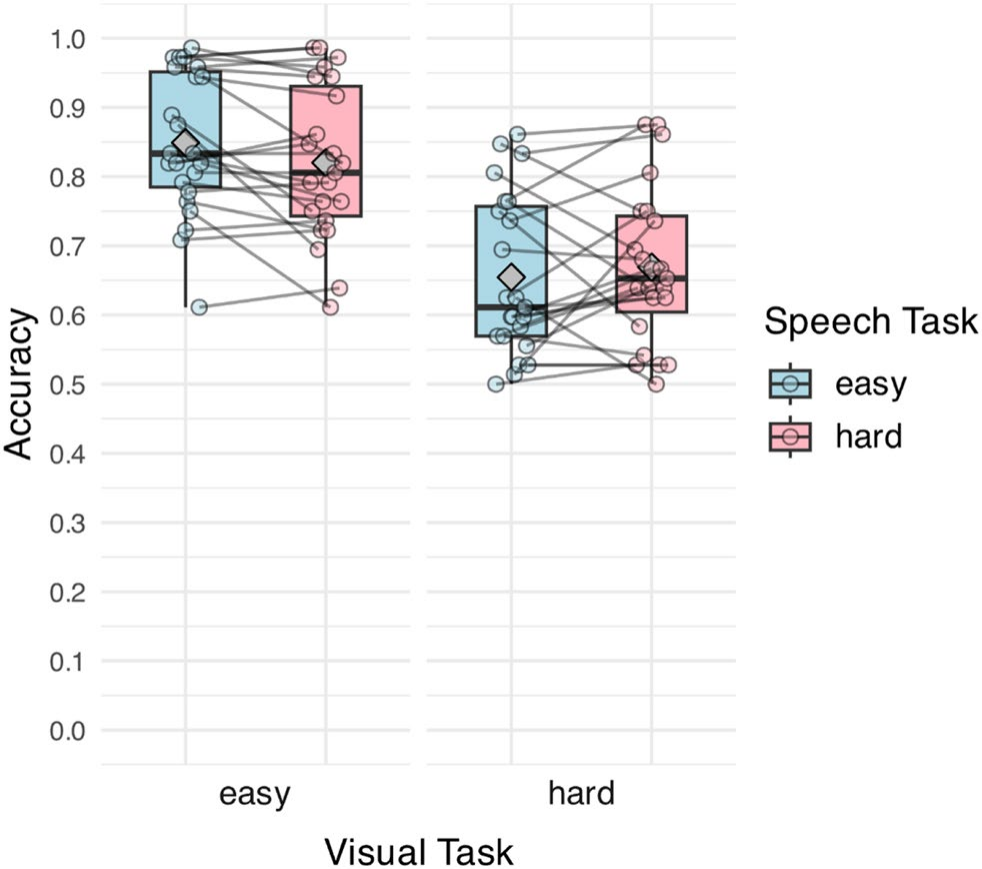
Accuracy of the visual task. The fill of the boxes represents speech‐task difficulty. The *X*‐axis shows visual‐task difficulty. Points display the mean response per participant, and lines connect the individual responses across conditions. Gray diamonds denote the mean of each task condition.

Figure 4 illustrates the visual‐task accuracy by visual‐ and speech‐task difficulty. The main effect of visual‐task difficulty and the interaction between the two tasks were significant (see Table 3 for model outputs). Visual‐task difficulty significantly modulated visual‐task accuracy—listeners performed significantly worse in the hard than in the easy visual task for both difficulty levels of the speech task (Sp E Vis E: 0.849 [SD = 0.10]; Sp E Vis H: 0.655 [SD = 0.12]; Sp H Vis E: 0.820 [SD = 0.11]; Sp H Vis H: 0.669 [SD = 0.11]; both *p*'s < 0.001). Notably, the speech task had a significantly larger impact on the visual‐task accuracy when the visual task was easy than when performing the hard visual task. Performing the hard speech task significantly worsened the performance of the visual task compared to when the speech task was easy [lower by a proportion of 0.029; *β* (SE) = −0.23 (0.10), *p* = 0.020], whereas visual‐task accuracy was comparable for both speech‐task levels under a hard visual task [*β* (SE) = −0.07 (0.07), *p* = 0.37].

**TABLE 3:** Model outputs for the GLMM assessing the fixed effects of Visual‐ and Speech‐task difficulty on Visual‐task accuracy.

	Df	Chisq	Chi Df	*p*
DiffSpeech	6	1.67	1	**0.197**
DiffVisual	6	28.07	1	**< 0.001**
DiffSpeech:DiffVisual	6	5.72	1	**0.017**

*Note:* Bold values indicate statistically significant values.

Abbreviations: Chi Df: the degrees of freedom associated with the chi‐squared distribution; Chisq: the fixed‐effect term's chi‐squared statistics (i.e., difference between the minus twice of the log likelihoods of the two models differing in that fixed‐effect term); Df: degrees of freedom of the fixed‐effect terms; DiffSpeech: Speech‐task difficulty; DiffVisual: Visual‐task difficulty.

#### 4.1.3 Effect of TD on Dual‐Task Performance

**FIGURE 5:**
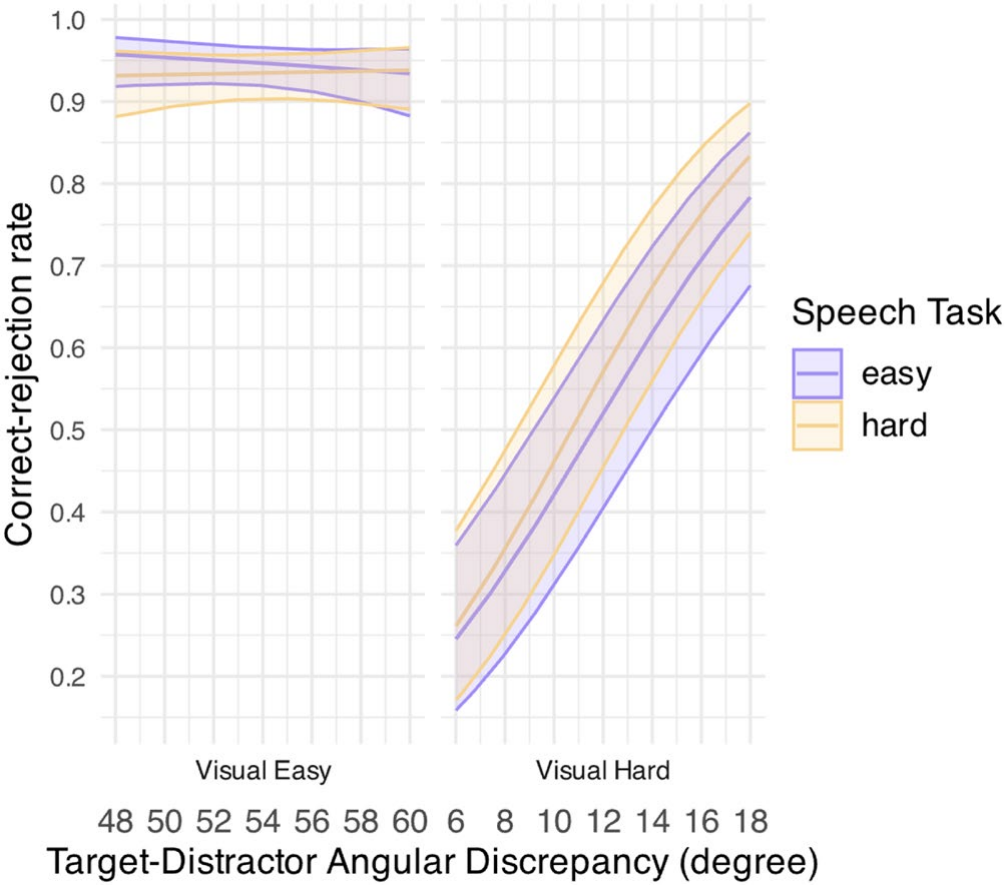
GLMM‐estimated correct‐rejection rate of the visual task for the nontarget trials, displayed as a function of target‐distractor angular discrepancy for an easy (middle purple line) and a hard speech task (middle yellow line) under an easy (left panel) and a hard visual task (right panel). Filled areas represent 95% confidence intervals.

As expected, the analysis on TD (Table 4 and Figure 5) further showed that listeners' visual‐task performance (i.e., correct‐rejection rate) improved significantly and equally with increasing TD under a hard visual task for both easy and hard speech conditions [easy speech: *β* (SE) = 0.20 (0.02), *p* < 0.001; hard speech: *β* (SE) = 0.22 (0.02), *p* < 0.001]. Although performance was below chance for a small TD (i.e., 6°–10°), distractors became discernable from a target for a TD larger than 10° (i.e., correct rejection > 0.5; easy speech: 11.4°, hard speech: 10.4°). By contrast, their visual‐task performance remained consistently high for the whole TD range under an easy visual task for both speech‐task conditions [easy speech: *β* (SE) = −0.04 (0.04), *p* = 0.355; hard speech: *β* (SE) = 0.01 (0.04), *p* = 0.806]. These results confirmed that task difficulty manipulated via TD effectively affected listeners' visual‐task performance and listeners were able to perform above chance in most cases even for a challenging visual task.

**TABLE 4:** Model outputs for the GLMM assessing the fixed effects of TD, Speech‐task difficulty, and Visual‐task difficulty on the visual‐task accuracy of the nontarget visual‐task trials.

Fixed effects				
	*β*	Std. error	*z*	*p*
(Intercept)	−1.12	0.28	−4.03	**< 0.001**
TD	0.20	0.02	8.16	**< 0.001**
SpeechHard [SpeechEasy_VisualHard]	0.08	0.23	0.37	**0.711**
VisualEasy [SpeechEasy_VisualHard]	4.23	0.42	10.01	**< 0.001**
TD:SpeechHard	0.02	0.03	0.59	**0.554**
TD:VisualEasy	−0.24	0.05	−5.00	**< 0.001**
SpeechHard:VisualEasy [SpeechEasy_VisualHard]	−0.58	0.45	−1.30	**0.193**
TD:SpeechHard:VisualEasy [SpeechEasy_VisualHard]	0.03	0.06	0.41	**0.681**

*Note:* The reference level is shown in brackets. Bold values indicate statistically significant values.

**FIGURE 6:**
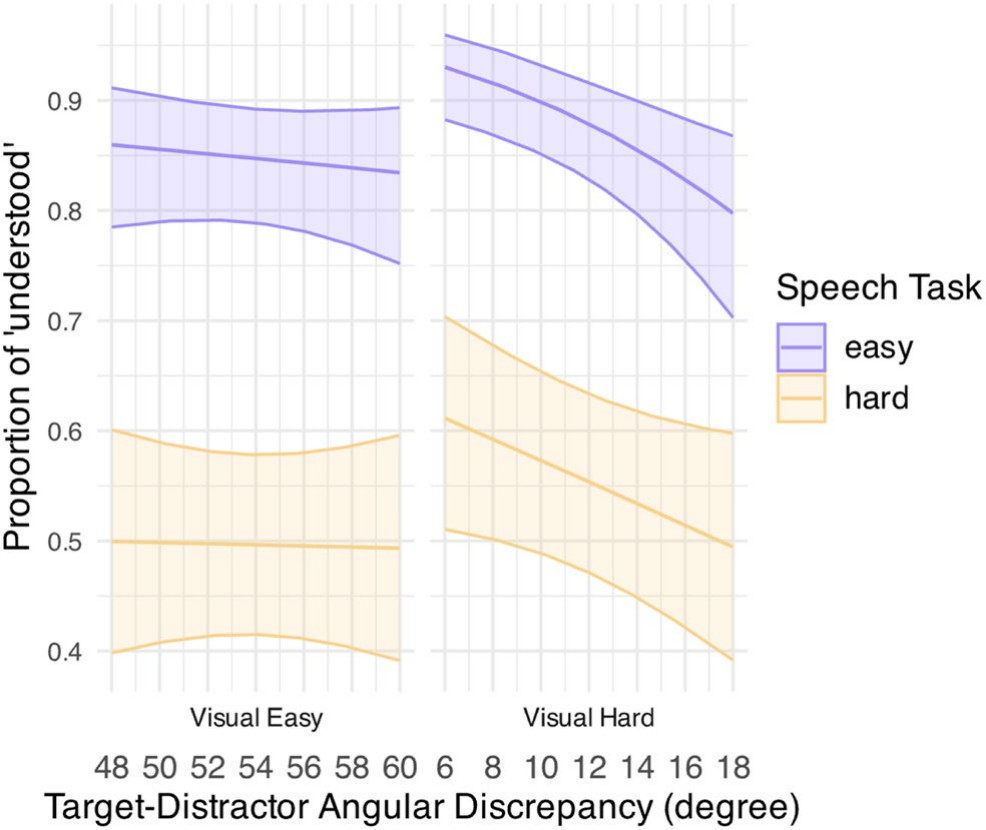
GLMM‐estimated proportion of sentence reported as “understood” for the speech task in the nontarget visual‐task trials, displayed as a function of target‐distractor angular discrepancy for an easy (middle purple line) and a hard speech task (middle yellow line) under an easy (left panel) and a hard visual task (right panel). Filled areas represent 95% confidence intervals.

The analysis on speech‐task accuracy showed that sentence recognition was also affected by TD (Table 5 and Figure 6). Notably, listeners' speech‐task performance generally deteriorated for a larger TD under a hard visual task. Specifically, speech performance seemed to drop significantly as TD became larger under an easy visual task [*β* (SE) = −0.10 (0.03), *p* < 0.001] but only worsened marginally for a larger TD when both tasks were challenging [*β* (SE) = −0.04 (0.02), *p* = 0.06]. Sentence recognition performance, however, remained consistently high for all TDs under an easy visual task for both speech‐task conditions [easy speech: *β* (SE) = −0.02 (0.03), *p* = 0.555; hard speech: *β* (SE) = 0.002 (0.02), *p* = 0.923]. As such, TD had an opposite effect on the two tasks' performance for the hard visual condition—listeners performed better in the visual task but worse in the speech task for a larger TD, particularly when the speech task was easy. These results insinuated a performance trade‐off between the two tasks when under a challenging visual task, where better visual‐task performance (i.e., better correct rejection for a larger TD) corresponded to worse speech‐task performance (i.e., worse “gist” report for a larger TD).

**TABLE 5:** Model outputs for the GLMM assessing the fixed effects of TD, Speech‐task difficulty, and Visual‐task difficulty on the speech‐task accuracy of the nontarget visual‐task trials.

Fixed effects				
	*β*	Std. error	*z*	*p*
(Intercept)	2.59	0.29	8.81	**< 0.001**
TD	−0.10	0.03	−3.34	**< 0.001**
SpeechHard [SpeechEasy_VisualHard]	−2.14	0.29	−7.32	**< 0.001**
VisualEasy [SpeechEasy_VisualHard]	−0.78	0.30	−2.59	**0.010**
TD:SpeechHard	0.06	0.04	1.67	0.096
TD:VisualEasy	0.09	0.04	2.08	**0.038**
SpeechHard:VisualEasy [SpeechEasy_VisualHard]	0.32	0.36	0.89	**0.376**
TD:SPeechHard:VisualEasy [SpeechEasy_VisualHard]	−0.05	0.05	−0.94	**0.349**

*Note:* The reference level is shown in brackets. Bold values indicate statistically significant values.

### 4.2 fMRI Results

#### 4.2.1 General Linear Model

**FIGURE 7:**
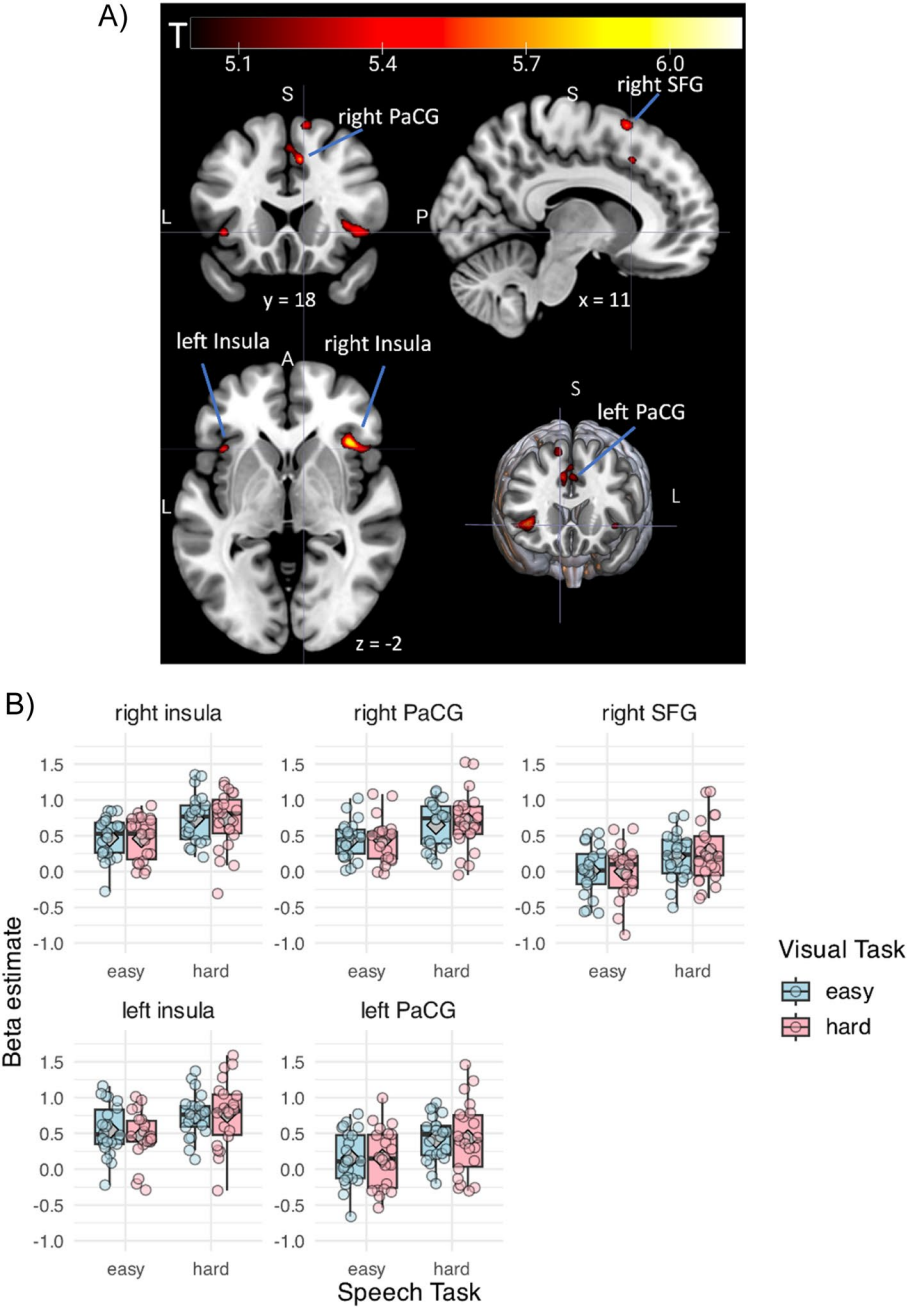
Effect of task difficulty on BOLD responses (i.e., *β* estimates for the canonical BOLD term from the individual GLMs). (A) The clusters of voxels showing a significant effect of speech hard > speech easy were displayed in warm colors. Colors of the significant clusters reflect the *t*‐statistic values of the contrast (*t* > 5.06, *p* < 0.05, family‐wise error corrected; cluster size > 0). (B) *X*‐axis shows speech‐task difficulty. Fill of the boxes represents visual‐task difficulty. Panels show brain regions. Points display the mean beta estimate per participant averaged across the voxels in that region. Gray diamonds denote the mean of each task condition.

Figure 7 shows the clusters of voxels illustrating a significant effect of speech hard > speech easy in the whole‐brain analysis (Figure 7A) and individual GLMs' mean *β* coefficient estimates for these clusters (Figure 7B). Table 6 outlines MNI coordinates for the voxel having a peak BOLD response in different clusters of activation (anatomical labels were obtained from Harvard‐Oxford cortical structural atlas; https://fsl.fmrib.ox.ac.uk/fsl/fslwiki/Atlases). Larger BOLD responses for the speech‐hard than speech‐easy conditions were found in bilateral insulae, bilateral PaCG, and right superior frontal gyrus (SFG), consistent with these regions responding to degradation in speech (Erb et al. 2013; Hervais‐Adelman et al. 2012; Ritz et al. 2022) and increased task demand under distraction (Gennari et al. 2018). No clusters showed a significant effect of visual‐task difficulty, nor an interaction between the two tasks.

**TABLE 6:** Clusters showing significant activation for speech hard > speech easy in the group‐level, whole‐brain GLM.

Cluster labels	*t*	*z*	*p*	MNI coordinates			Number of voxels in the cluster
				*x*	*y*	*z*	
Right insula	6.14	5.57	0.001	38	22	0	101
Right PaCG	5.90	5.38	0.002	8	20	42	49
Right SFG	5.80	5.31	0.004	12	14	62	34
Left insula	5.60	5.15	0.007	−38	18	−2	11
Left PaCG	5.52	5.09	0.010	−2	26	42	20

*Note:* Parameter estimates were thresholded at *t* > 5.06, *p* < 0.05, family‐wise error corrected. Coordinates show the voxels having a peak BOLD response. Clusters containing more than 0 voxels were displayed.

#### 4.2.2 Correlation Analysis

**FIGURE 8:**
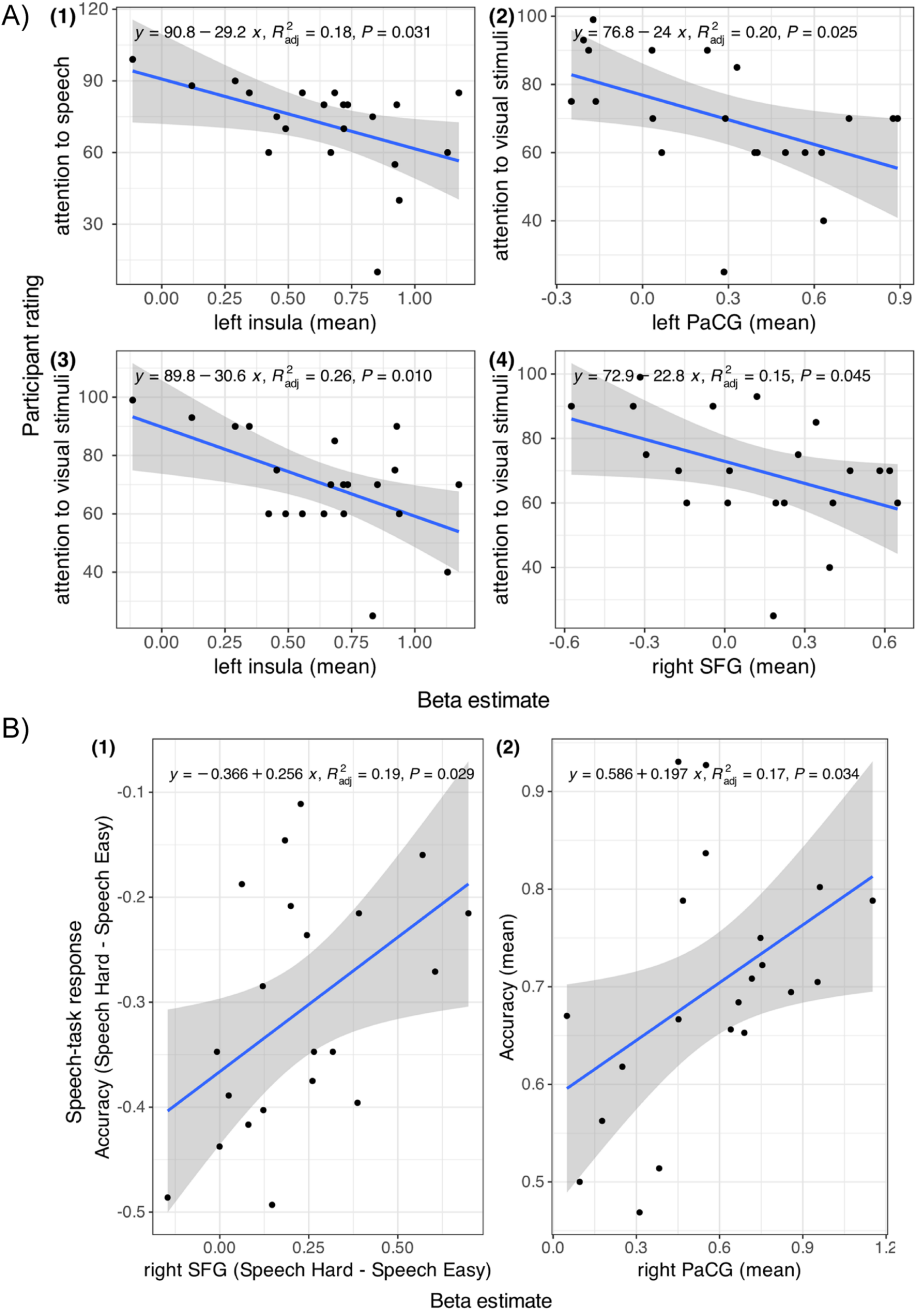
Simple regression models examining the relationship between the subjective attention ratings and BOLD responses (A), as well as between behavioral and BOLD responses (B). Points display the measures per participant. Filled areas represent 95% confidence intervals. Each model's regression function, model fit (*R* ^2^), and *p* value are displayed at the top‐left corner of each panel.

Figure 8 examines the relationships between BOLD responses (i.e., GLM *β* estimates for the clusters showing a significant effect of speech hard > speech easy), scores for the effort and attention questionnaire, as well as task behavioral responses. The figure shows the pairs of measures displaying a significant effect from an exploratory correlation analysis (see Figure C2).

In general, there was a negative correlation between the mean BOLD responses (across conditions) and the overall effort and attention scores—mean activity in left insula negatively correlated with the estimated attention to speech task (Figure 8A.1), and likewise for the activities in left PaCG (Figure 8A.2), left insula (Figure 8A.3), and right SFG (Figure 8A.4) negatively correlated with the attention score for the visual task. That is, listeners who showed stronger activation in the corresponding regions tended to report less attention to either task.

In addition, there was a positive correlation between the difference in right SFG responses for the hard and easy speech conditions and the difference in speech task response for these conditions (Figure 8B.1)—listeners who showed a more elevated activation for the hard compared to easy speech task had a lower loss in speech‐task performance when conducting a hard versus easy speech task. There was also a positive correlation between the mean responses in right PaCG and the mean speech‐task performance across conditions (Figure 8B.2)—listeners who had a stronger activation in right PaCG tended to also perform better in the speech task. These results suggest responses to acoustic degradation in the frontal and cingulate cortices were associated with attentional modulation across the concurrent tasks and alleviating degradation in speech (Gennari et al. 2018). These results suggest that increased BOLD responses in SFG and PaCG might have helped elicit better task performance in listeners.

#### 4.2.3 XGBoost

##### 4.2.3.1 Baseline (Whole Brain) Model

**FIGURE 9:**
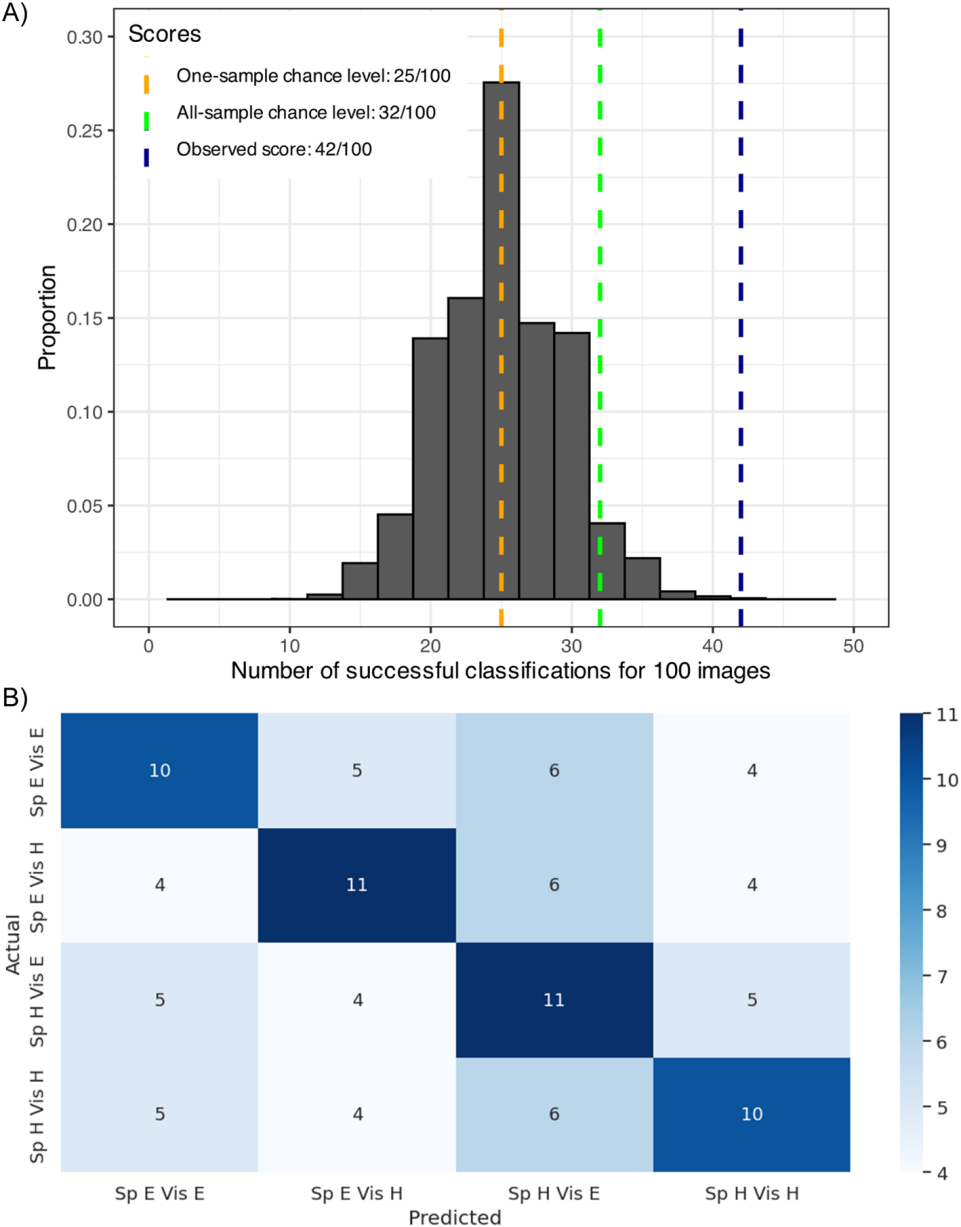
(A) A theoretical binomial distribution of 5760 samples (i.e., the number of tested hyperparameters) each having a sample size of 100 and a success rate of 0.25. *X*‐axis shows the number of successes for classifying 100 images, and the *Y*‐axis shows the proportion for each bin out of all samples. The chance‐level score for one binomial sample is 25/100 (i.e., orange dashed line). The chance‐level score given that the best model was picked out of 5760 hyperparameter configurations is 32/100, corresponding to a probability of 4.5% for observing a larger score given the null hypothesis is true (i.e., *p* = 0.045; green dashed line). The observed accuracy was 42/100 (*p* < 0.001; navy dashed line). (B) The confusion matrix represents the predicted against actual labels for each task condition for all 25 participants during the cross validation of the whole‐brain model. Rows correspond to the actual task conditions and columns indicate the predicted task conditions. Numbers in the cell show the number of participants corresponding to the pair of actual and predicted labels. The color intensity of tiles reflects numeric value. Sp E Vis E: speech easy visual easy; Sp E Vis H: speech easy visual hard; Sp H Vis E: speech hard visual easy; Sp H Vis H: speech hard visual hard.

The first step of the XGBoost analysis was to establish a baseline model using input features covering the whole brain for subsequent feature selection. The model with 400 feature inputs based on the Schaefer atlas (Schaefer et al. 2018) produced the best accuracy (among all tested number of features)—42% for the prediction during a five‐fold cross validation (see Table 1 for best hyperparameters). An accuracy of 100% would indicate a successful prediction of all items in the validation set, whereas a random classification would result in a chance‐level accuracy of 25%. The chance‐level score given that the best model was selected from a binomial distribution of 5760 scores each having a sample size of 100 and a probability of success of 0.25 is 32/100. This score corresponds to a probability of 4.5% for observing a larger score given the null hypothesis is true (i.e., *p* = 0.045; green dashed line in Figure 9A). The binomial test comparing the best accuracy against this null distribution further confirmed that the model performed significantly higher than chance (*p* < 0.001; navy dashed line in Figure 9A). The confusion matrix suggested that the model performed equally well for classifying all four task conditions, with an accuracy between 40% and 44% and an error rate between 16% and 24% per misclassified class (Figure 9B).

##### 4.2.3.2 Model With Selected Features

**FIGURE 10:**
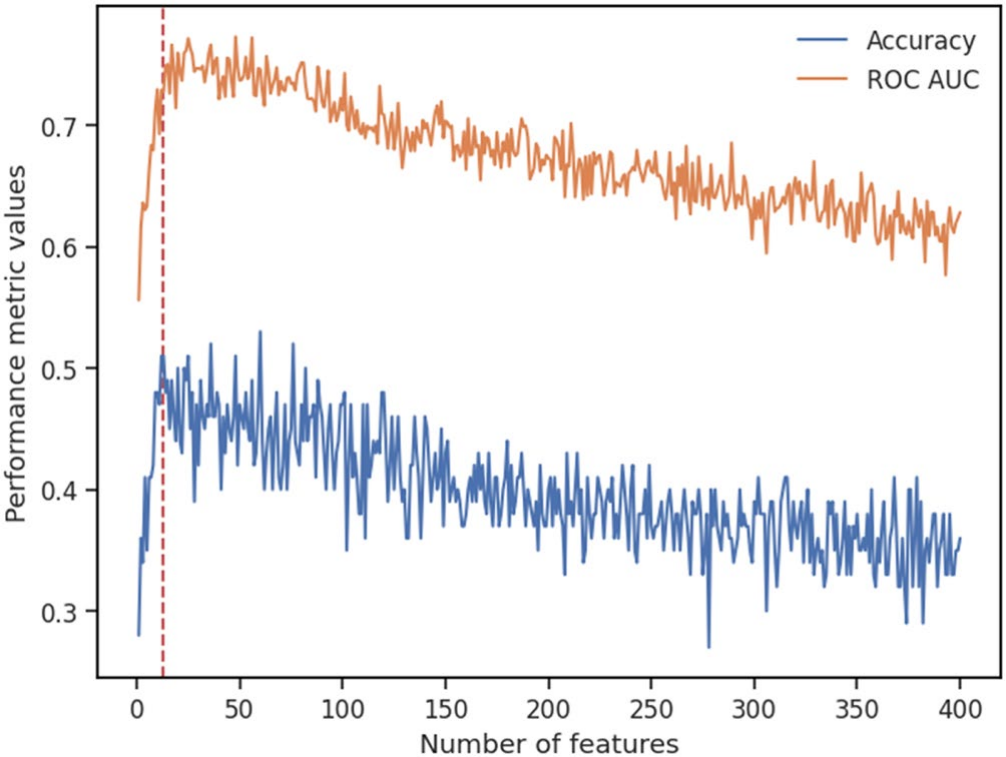
The performance metrics of the models containing different number of features. Models were fitted with features having top mean SHAP values iteratively added to the model. Fills of the lines show performance metrics: The accuracy and the area under the receiver operating characteristic curve (ROC AUC) of the test batches. The ROC was the true positive rate plotted as a function of the false positive rate for two classes. The AUC was the average AUC for all possible pairwise combinations of classes. A perfect model would score an AUC of 1.0 while a random classification would score 0.5. The red dashed line shows the number of selected features for further analyses.

To identify cortical areas that most significantly contributed to the classification of task conditions for subsequent feature selection, feature importance was evaluated by SHAP values (see Figure C3 for feature ranking and Table A2 for the correspondent MNI coordinates). Figure 10 shows the performance metrics of the models fitted iteratively with top features added to the model. Validation accuracy peaked at the 12th iteration (i.e., 51%) and no improvements were observed for four consecutive iterations thereafter (until the 16th iteration). Therefore, the top 13 features were selected as the corresponding model had the last best accuracy (51%) within these 16 iterations. An extensive grid search finding the best hyperparameters (Table B1) for the 13‐feature model yielded a validation accuracy of 60% during cross validation.

**FIGURE 11:**
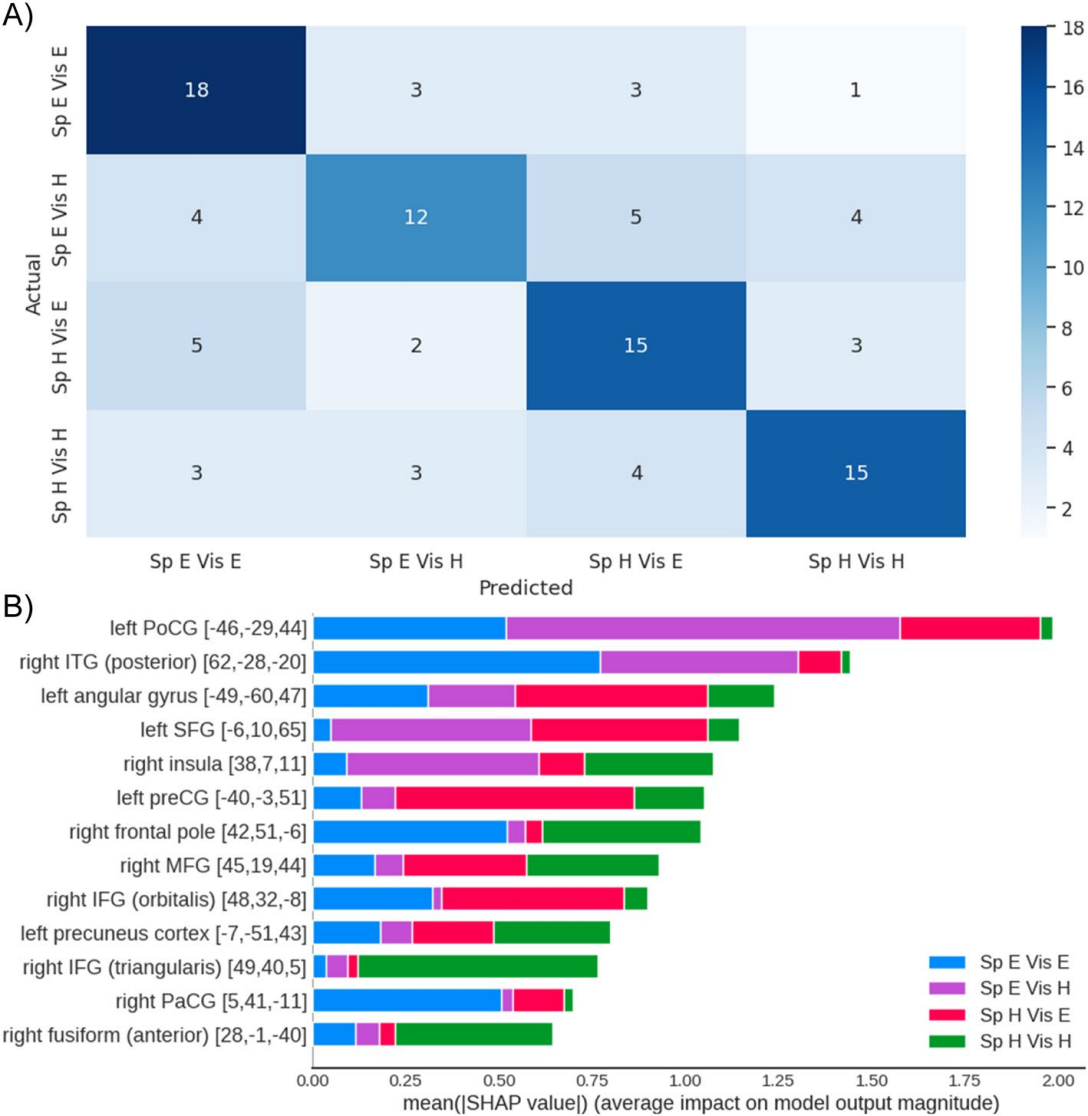
(A) The confusion matrix represents the predicted against actual labels for each task condition for all 25 participants during the cross validation of the top 13‐feature model with best hyperparameters. Rows correspond to the actual task conditions and columns indicate the predicted task conditions. Numbers in the cell show the number of participants corresponding to the pair of actual and predicted labels. The color intensity of tiles reflects numeric value. (B) Top 13 features ranked by their contribution (i.e., SHAP values) to classifying task conditions. *X*‐axis displays the absolute SHAP values per class averaged across all data points. Color bars illustrate classes (i.e., task conditions), and the width of each bar represents the impact of a specific feature on differentiating a certain class from all other classes. MNI coordinates were shown in brackets. ITG: inferior temporal gyrus; MFG: middle frontal gyrus; PoCG: postcentral gyrus; preCG: precentral gyrus; SFG: superior frontal gyrus; Sp E Vis E: speech easy visual easy; Sp E Vis H: speech easy visual hard; Sp H Vis E: speech hard visual easy; Sp H Vis H: speech hard visual hard.

Figure 11 further illustrates the confusion matrix (Figure 11A), and feature importance (i.e., mean SHAP value; Figure 11B) for the 13‐feature model. The top features included regions that were also identified in the GLM analysis—right insula and right PaCG. The features also contained regions previously reported for degraded speech processing: left SFG (McGettigan et al. 2017), left angular gyrus (Eisner et al. 2010), left precentral gyrus (Hervais‐Adelman et al. 2012; Nuttall et al. 2016, 2022; Sohoglu et al. 2012), and left postcentral gyrus (McGettigan et al. 2017); visuospatial processing: left precentral gyrus (Windischberger et al. 2003), right ITG (Adab et al. 2014; Jackson et al. 2018; Zhang et al. 2011); and task‐performance monitoring: right frontal pole (Koechlin 2011; Tsujimoto et al. 2011). Specifically, the following regions were also reported for their roles in attending to auditory or visuospatial signals: right IFG, left SFG, right MFG, left precentral gyrus and postcentral gyrus, left angular gyrus, left precuneus, and right fusiform (Balslev et al. 2013; Degerman et al. 2006; Gennari et al. 2018; Hampshire et al. 2010; Japee et al. 2015; Johansen‐Berg and Matthews 2002).

##### 4.2.3.3 Model Interpretation

**FIGURE 12:**
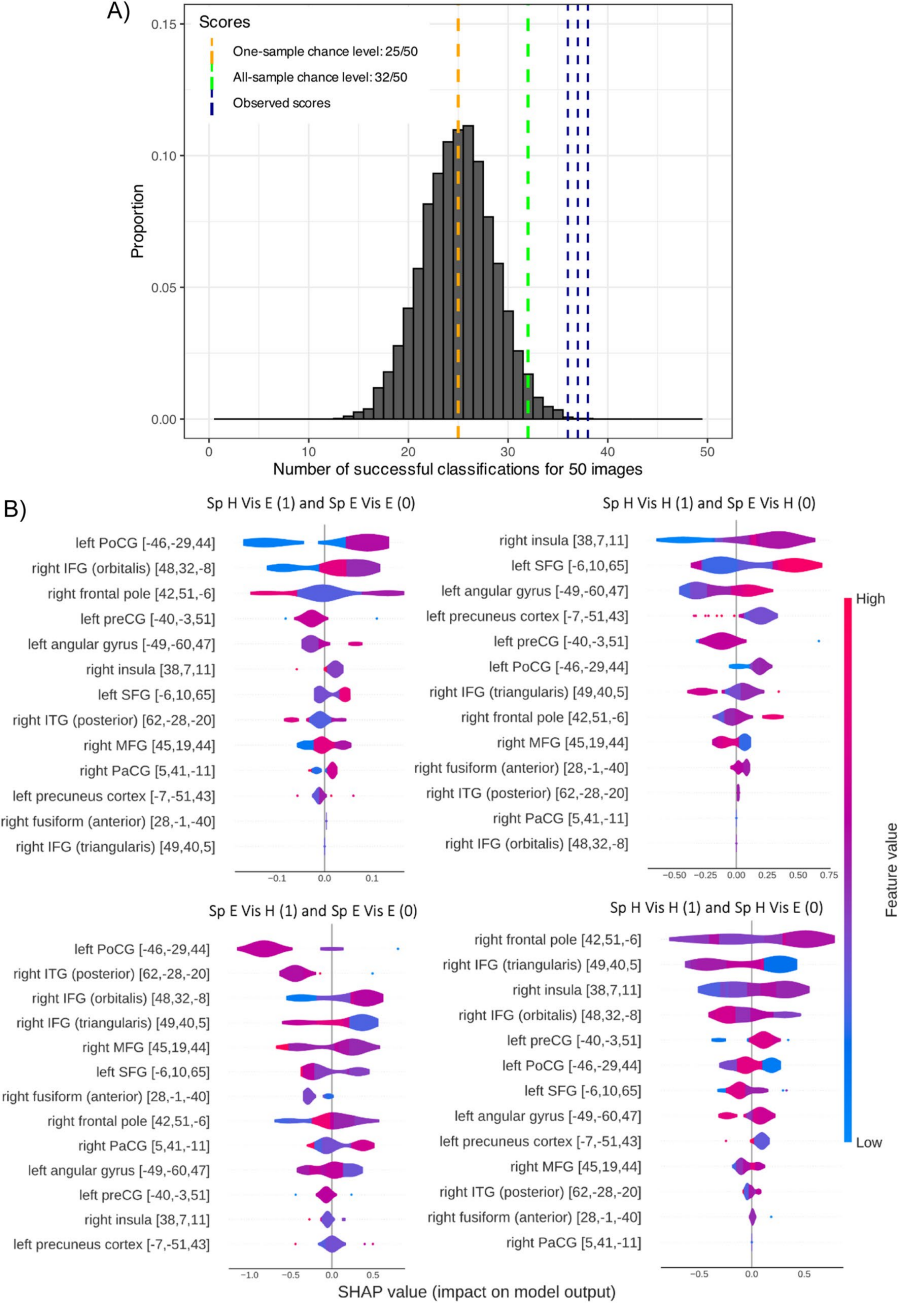
(A) A theoretical binomial distribution of 5760 samples (i.e., the number of tested hyperparameters) each having a sample size of 50 and a success rate of 0.5. *X*‐axis shows the number of successes for classifying 50 images, and the *Y*‐axis shows the proportion for each bin out of all samples. The chance‐level score for one binomial sample is 25/50 (i.e., orange dashed line). The chance‐level score given that the best model was picked out of 5760 hyperparameter configurations is 32/50 (64%), corresponding to a probability of 1.64% for observing a larger score given the null hypothesis is true (i.e., *p* = 0.0164; green dashed line; *p*‐threshold corrected by Bonferroni's method to 0.0125). The observed scores were 38/50 (76%), 38/50 (76%), 36/50 (72%), and 37/50 (74%) (all *p*s < 0.001; navy dashed lines). (B) The summary plot of how the top 13 features impact on the models' prediction. Each panel displays a binary classification model for pairwise task classes (coded as 1 and 0). The density curves per feature show the distributions of samples with regard to the SHAP values (*X*‐axis). Color of the density curve displays the distribution of the original values of a feature over the sample. In each binary classifier (i.e., each panel), a positive Shapley value for a feature suggests that the presence of the feature increases the model's prediction toward class 1, whereas a negative Shapley value for a feature indicates that the presence of that feature decreases the model's prediction for class 1. MNI coordinates were shown in brackets.

Figure 12 illustrates the results of the binary classification models based on the top 13 features. The chance‐level score given that the best model was selected from 5760 hyperparameter scores is 32/50 (64%), corresponding to a probability of 1.64% for observing a larger score given the null hypothesis is true (i.e., *p* = 0.0164; green dashed line in Figure 12A). Here, *p*‐threshold was corrected by Bonferroni's method to 0.0125 as there were four classification models. The binomial test confirms that all models had an accuracy surpassing the chance level of 64% (see Figure 12A for the binomial test results, Table 7 for the models' accuracy scores and Table B3 for best hyperparameters). Figure 12B shows how neural responses of the top 13 regions affect the predictions of the binary classification models containing classes differing in their task difficulty. Table 7 further expatiates the neural responses per region corresponding to a positive prediction (i.e., label 1) in the binary classification, revealing the effects of task difficulty on neural activation patterns. These binary classification models revealed responses that were sensitive to both speech‐ and visual‐task difficulties, that is, right PaCG, right insula, right IFG (orbitalis), and right MFG (i.e., modality‐general responses). The models also identified modality‐specific, raised responses either to a harder speech task (i.e., left SFG, left postcentral gyrus, left angular gyrus, right frontal pole) or a more difficult visual task (i.e., right ITG, precentral gyrus; see Section 5 for more details).

**TABLE 7:** Neural responses of the top 13 features predicting a positive label (1) for binary classification models built to examine how task difficulty impacts neural activation patterns (also see Figure 12).

Task	Pairwise classes	Neural responses per region predicting a positive label (1)	
		Elevated response	Alleviated response
Speech	Sp H Vis E (1) and Sp E Vis E (0) Classification accuracy: 76%	Left postcentral gyrus, right IFG (orbitalis), left angular gyrus, right insula, left SFG, right MFG, right PaCG	Right frontal pole, right pITG
	Sp H Vis H (1) and Sp E Vis H (0) Classification accuracy: 76%	Right insula, left SFG, left angular gyrus, left postcentral gyrus, right frontal pole, right fusiform	Left precuneus cortex, right IFG (triangularis), right MFG
Visual	Sp E Vis H (1) and Sp E Vis E (0) Classification accuracy: 72%	Right IFG (orbitalis), right PaCG	Right IFG (triangularis), right MFG, left SFG, left angular gyrus
	Sp H Vis H (1) and Sp H Vis E (0) Classification accuracy: 74%	Right insula, left precentral gyrus, right MFG, right pITG	Right IFG (triangularis), right IFG (orbitalis), left postcentral gyrus, left SFG, left precuneus cortex

*Note:* Labels of classes were coded as 1 and 0. The table only includes regions showing a positive impact (i.e., a positive SHAP value) on model predictions.

## 5 Discussion

### 5.1 Behavioral Findings

Using a dual‐task paradigm and ML, this study established how acoustic degradation and divided attention jointly affected speech processing. Behaviorally, increasing the difficulty of one task impeded the accuracy of that task. The performance of one task was also modulated by the difficulty of the concurrent task. Specifically, speech recognition was better during a hard than an easy visual task, consistent with previous findings for noise‐vocoded speech processing under a visual recognition task (Ward et al. 2017). Despite no explicit instruction given for prioritizing either task, this finding suggests that listeners might have prioritized the speech task performance. Specifically, if participants had prioritized performing the visual task over the speech task, a more demanding visual task would have hampered the speech performance as a hard visual task would have drawn more resources from the speech task than an easy visual task does (Gennari et al. 2018). This interpretation has also been corroborated by Ward et al. (2017), where listeners were explicitly instructed to prioritize speech task performance in a visual dual task and showed increased speech performance under a hard than an easy visual task.

Visual‐task performance, on the other hand, was negatively affected by the speech task, also in line with the findings by Ward et al. (2017), signaling increased listening effort for processing more degraded speech (Johnsrude and Rodd 2015; Rodd et al. 2010). The fact that this load effect was only observable for an easy task insinuates that task performance might be constrained by the availability of processing resources (Kahneman 1973). The hard visual task was challenging as it had a very small TD and was equally challenging for either speech task. Listeners would have showed slightly better visual performance under an easy speech task than under a hard one if they were able to spare more resources for the visual task. Finally, there was also a continuous positive effect of TD on visual‐task responses for the hard visual condition, which had an opposite effect on the speech‐task response. These findings insinuate the competition between the two tasks for limited processing resources (Kahneman 1973).

### 5.2 Neuroimaging Findings With GLM

In the GLM analysis, performing a hard speech task (recognizing four‐band instead of eight‐band speech) resulted in increased BOLD responses in regions related to attentional control (i.e., PaCG; Gennari et al. 2018) and increased task demand (i.e., insula, SFG; Erb et al. 2013; Hervais‐Adelman et al. 2012; Ritz et al. 2022; Zekveld et al. 2014). The results are consistent with previous reports finding insula and SFG for their association with increased listening effort (Erb et al. 2013; Hervais‐Adelman et al. 2012), including studies using objective measures for listening effort with pupillometry (Zekveld et al. 2014). Prior studies also showed the involvement of PaCG for attentional allocation as a function of processing load for ambiguously produced syllables under divided attention (Gennari et al. 2018). Our study extends this role of PaCG to processing sentence‐level degraded speech. The neuroimaging findings are corroborated by the correlation of activation in these regions to listeners' behavioral task performance and self‐reported attention score (Figure 8). Taken together, our results associate a set of frontal and cingulate regions with resolving the elevated processing load of noise‐vocoded speech under divided attention.

### 5.3 Neuroimaging Findings With XGBoost

The current study implemented XGBoost as a data‐driven approach to explore the spatially dispersed and nonlinear neural activation patterns across the four dual‐task conditions. Specifically, the pipeline involved (1) a whole‐brain model to establish baseline performance, (2) feature ranking and selection according to Shapley values to reduce data dimensionality, (3) an optimal model fitted with the selected top features, and (4) binary classification models fitted with the selected features, enabling pairwise model interpretation with Shapley values. The optimal model predicted task conditions from neural responses with high accuracy and expanded the GLM findings by highlighting contributions on model predictions from 13 frontotemporal regions. The analysis unveiled greater BOLD responses in the right insula, right PaCG, and left SFG for increased speech‐task difficulty, corroborating with the GLM results. The analysis further revealed neural responses that are general or specific to perceptual modalities (i.e., auditory or visual). Specifically, right PaCG, right insula, right IFG (orbitalis), and right MFG were sensitive to both speech‐ and visual‐task difficulties, which might relate these regions to attentional control across modalities. The finding is consistent with past evidence showing overlapped activations in PaCG and insula with increased task difficulty during selective attention to recognizing degraded (low‐pass filtered) words or to deciding on the direction of a visual arrow (Eckert et al. 2009). Likewise, a previous study asked participants to actively listen out for a pitch change in a target sound embedded in naturalistic sounds and search for a color change of a target object in a naturalistic visual scene (Braga et al. 2013). The authors found that both tasks were associated with elevated responses in the right IFG and right MFG, corroborating with the current finding.

In regard to modality‐specific responses for speech‐task difficulty, XGBoost discovered increased responses to larger acoustic degradation under both visual‐task conditions in left SFG, left postcentral gyrus, and angular gyrus. Left SFG responses resonate with our GLM findings. McGettigan et al. (2017) found left postcentral gyrus sensitive to increased acoustic degradation under noise vocoding. Eisner et al. (2010) showed that elevated responses in left angular gyrus were associated with the learnability of noise‐vocoded speech: neural activations increased over the exposure to learnable (i.e., intelligible) speech but not for spectrally inverted speech. Therefore, the activation patterns for left postcentral gyrus and angular gyrus in the current study might reflect degradation and learnability differences across the two speech‐task conditions. The analysis also found degradation‐related response under a hard visual task in frontal pole, a region reported for self‐monitoring of performance, especially for errors that are internally recognized and are not accompanied by explicit feedback (e.g., Ham et al. 2013; Sharp et al. 2010). More importantly, XGBoost detected distinct activation patterns for visual‐task difficulty, which was unobserved in the GLM analysis. Modality‐specific, more pronounced responses in left precentral gyrus and right pITG were found for increasing the visual‐task difficulty under a hard speech task. This finding is consistent with the previous evidence for elevated responses in precentral gyrus related to mental rotation of a visual object (Windischberger et al. 2003) and in ITG associated with attending to specific features in an object for the purpose of visual recognition (Sigala and Logothetis 2002). As such, the finding illustrates pronounced feature‐level processing related to performing a hard visual task.

Critically, the XGBoost model also revealed neural signatures correlated with attentional allocation of resources across the two tasks: regions showing a modality‐specific response to higher speech‐task difficulty (i.e., left SFG, left postcentral gyrus) had a suppressed response to elevated visual‐task difficulty. Conversely, regions having a modality‐specific response to increased visual‐task difficulty showed a suppressed response to lifted speech‐task difficulty (i.e., right pITG). These results resonate with Gennari et al. (2018), where visual‐task difficulty decreased auditory‐related activity under divided attention. As with Gennari et al. (2018), the trade‐off between dual‐task performance in the current study is likely to be modulated by activities in attentional‐control regions including PaCG and insula. This account is corroborated by the negative relationship between PaCG/insula and listeners' self‐reported attention to the visual task. As such, these findings revealed the neural underpinnings for the interaction of acoustic degradation and divided attention in affecting speech processing—the BOLD signal and performance trade‐off between the speech and a concurrent task under paracingulate attentional control.

In summary, XGBoost extended the GLM findings by identifying a set of 13 frontal‐temporal regions, which highlighted both modality‐general responses in right PaCG, right insula, right IFG, and right MFG related to attentional control over concurrent tasks, as well as modality‐specific responses for respective processing of degraded speech (i.e., left postcentral gyrus, left angular gyrus, right frontal pole) and visual input (i.e., right ITG, precentral gyrus) under divided attention, which were undiscovered by GLM. The analysis also revealed dynamic resource dispensing between the two tasks in these modality‐specific regions.

### 5.4 Limitations

MRI scanner generates significant acoustic noise while scanning, for example, 110 dB for our 3‐Tesla PRISMA scanner, and can attenuate listening‐task performance and related brain activations (Elliott et al. 1999; Hall et al. 2009). A common method to counteract the noise effects during auditory experiment is sparse sampling, which introduces silent periods during stimulus presentation at the cost of the number of acquired volumes (i.e., statistical power; Okada and Nakai 2003). Another common scanning sequence is Interleaved Silent Steady‐State Imaging (ISSS), which acquires images in brief bursts that are interleaved with silent periods (Schwarzbauer et al. 2006). This method was shown to be statistically more sensitive than the traditional sparse sampling but was not available at BUCNI (Mueller et al. 2011). As such, we instead incorporated passive noise attenuation using earbuds sitting deep in ear canals, which is one of the most widely used methods for countering scanner noise and was shown to reduce the scanner noise to sufficiently low levels (i.e., by 25–30 dB) for task performance (Moelker and Pattynama 2003). All participants reported that they could hear the stimuli clearly amid the dampened background noise after the in‐scanner practice run.

Our speech task used “gist” report as a proxy for the performance of sentence understanding, following Wild et al. (2012) to minimize the potential in‐scanner head movements induced by a spoken response. As a trade‐off, the response measures how well the participants think they understood the sentences, instead of a direct reflection on how well they understood the sentences. Therefore, listeners might have inaccurately estimated their true performance. Nevertheless, as discussed above, our speech‐task responses illustrated a strong effect of speech‐task difficulty, and the better performance under a hard visual task aligned well with the findings of Ward et al. (2017), where keyword‐based, spoken responses were recorded for BKB sentences. Importantly, the dual‐task accuracy in Ward et al. (2017) for both 8‐band and 4‐band speech (0.97 and 0.73) was higher than those in the current study (Sp E Vis E: 0.86, Sp H Vis E: 0.53), under a visual task that had a similar performance to our easy visual condition (current: 0.84, Ward et al.: 0.87). Moreover, the 8‐band and 4‐band performances in the current study also bracket that of the 6‐band performance (0.61) from our previous dual‐task study using the same speech stimuli but with a spoken response (Wang et al. 2023). Finally, this issue was also partially addressed by the inclusion of the post‐scan surprise memory task, where listeners had a better recall for the easy speech than for the hard speech condition. As such, despite not providing a direct measure for speech performance, it is not likely that our listeners overestimated what they understood or provided an inaccurate estimate intentionally.

### 5.5 Summary

Overall, our results showed that manipulating speech and visual task difficulties under a dual task engages brain regions related to acoustic degradation, visual processing, and divided attention. Specifically, degraded speech processing under divided attention was associated with elevated responses in the superior frontal, cingulate, and insular cortices for increased degradation in speech. High accuracy and explainable prediction models of XGBoost further uncovered 13 frontotemporal brain areas for resource allocation under divided attention, featuring cross‐regional activations sensitive to processing degraded speech (i.e., left SFG, left postcentral gyrus, left angular gyrus, right frontal pole), or visuospatial content (i.e., right ITG, precentral gyrus), or both (i.e., right PaCG, right insula, right IFG, and right MFG). A dissociable effect of speech and visual tasks was found in left SFG/left postcentral gyrus and right ITG, uncovering the joint effect of acoustic degradation and divided attention in speech processing.

## Author Contributions

H.W., C.M., S.R., and P.A. designed the research. H.W. built the experimental paradigm. H.W. and R.C. collected data. H.W. and J.S. contributed to analytic tools and pipelines. H.W. analyzed data. H.W. wrote the first draft of the manuscript. H.W., R.C., J.S., C.M., S.R., and P.A. edited the manuscript.

## Conflicts of Interest

The authors declare no conflicts of interest.

## Supporting information


**Data S1:** Supplemental materials.

## Data Availability

The data that support the findings of this study are openly available in GitHub at https://github.com/hwanguc/brain‐xgboost‐dividedattn‐wangetal.
