# Supplementary material for: Neural Processing of Noise‐Vocoded Speech Under Divided Attention: An fMRI‐Machine Learning Study
Source: Hum Brain Mapp. 2025 Aug 7;46(11):e70312. doi: 10.1002/hbm.70312 (PMC12329574; doi:10.1002/hbm.70312)
Supplement: Supplementary file 1 — Data S1: Supplemental materials. [file HBM-46-e70312-s001.docx]

# Supplemental Materials

A. Stimuli and materials

Table A1. The sentence stimuli pool for the speech task.

| List | Sentence | Keyword Count | Keyword 1 | Keyword 2 | Keyword 3 | Keyword 4 |
| --- | --- | --- | --- | --- | --- | --- |
| BKB | The clown had a funny face | 3 | clown | funny | face | NA |
| BKB | The car engine's running | 3 | car | engines | running | NA |
| BKB | She cut with her knife | 3 | She | cut | knife | NA |
| BKB | Children like strawberries | 3 | Children | like | strawberries | NA |
| BKB | The house had nine rooms | 3 | house | nine | rooms | NA |
| BKB | They’re buying some bread | 3 | Theyre | buying | bread | NA |
| BKB | The green tomatoes are small | 3 | green | tomatoes | small | NA |
| BKB | He played with his train | 3 | He | played | train | NA |
| BKB | The postman shut the gate | 3 | postman | shut | gate | NA |
| BKB | They’re looking at the clock | 4 | Theyre | looking | at | clock |
| BKB | The bag bumps on the ground | 3 | bag | bumps | ground | NA |
| BKB | The boy did a handstand | 3 | boy | did | handstand | NA |
| BKB | A cat sits on the bed | 4 | cat | sits | on | bed |
| BKB | The lorry carried fruit | 3 | lorry | carried | fruit | NA |
| BKB | The rain came down | 3 | rain | came | down | NA |
| BKB | The ice cream was pink | 3 | ice | cream | pink | NA |
| BKB | The ladder's near the door | 3 | ladders | near | door | NA |
| BKB | They had a lovely day | 3 | They | lovely | day | NA |
| BKB | The ball went into the goal | 4 | ball | went | into | goal |
| BKB | The old gloves are dirty | 3 | old | gloves | dirty | NA |
| BKB | He cut his finger | 3 | He | cut | finger | NA |
| BKB | The thin dog was hungry | 3 | thin | dog | hungry | NA |
| BKB | The boy knew the game | 3 | boy | knew | game | NA |
| BKB | Snow falls at Christmas | 3 | Snow | falls | Christmas | NA |
| BKB | She’s taking her coat | 3 | Shes | taking | coat | NA |
| BKB | The police chased the car | 3 | police | chased | car | NA |
| BKB | A mouse ran down the hole | 4 | mouse | ran | down | hole |
| BKB | The lady’s making a toy | 3 | ladys | making | toy | NA |
| BKB | Some sticks were under the tree | 3 | sticks | under | tree | NA |
| BKB | The little baby sleeps | 3 | little | baby | sleeps | NA |
| BKB | They’re watching the train | 3 | Theyre | watching | train | NA |
| BKB | The school finished early | 3 | school | finished | early | NA |
| BKB | The glass bowl broke | 3 | glass | bowl | broke | NA |
| BKB | The dog played with a stick | 3 | dog | played | stick | NA |
| BKB | The kettle's quite hot | 3 | kettles | quite | hot | NA |
| BKB | The farmer keeps a bull | 3 | farmer | keeps | bull | NA |
| BKB | They say some silly things | 4 | They | say | silly | things |
| BKB | The lady wore a coat | 3 | lady | wore | coat | NA |
| BKB | The children are walking home | 3 | children | walking | home | NA |
| BKB | He needed his holiday | 3 | He | needed | holiday | NA |
| BKB | The milk came in a bottle | 3 | milk | came | bottle | NA |
| BKB | The man cleaned his shoes | 3 | man | cleaned | shoes | NA |
| BKB | They ate the lemon jelly | 4 | They | ate | lemon | jelly |
| BKB | The boys running away | 3 | boys | running | away | NA |
| BKB | Father looked at the book | 3 | Father | looked | book | NA |
| BKB | She drinks from her cup | 3 | She | drinks | cup | NA |
| BKB | The room's getting cold | 3 | rooms | getting | cold | NA |
| BKB | A girl kicked the table | 3 | girl | kicked | table | NA |
| BKB | The wife helped her husband | 3 | wife | helped | husband | NA |
| BKB | The machine was quite noisy | 3 | machine | quite | noisy | NA |
| BKB | The old man worries | 3 | old | man | worries | NA |
| BKB | A boy ran down the path | 3 | boy | ran | path | NA |
| BKB | The house had a nice garden | 3 | house | nice | garden | NA |
| BKB | She spoke to her son | 4 | She | spoke | to | son |
| BKB | They’re crossing the street | 3 | Theyre | crossing | street | NA |
| BKB | Lemons grow on trees | 3 | Lemons | grow | trees | NA |
| BKB | He found his brother | 3 | He | found | brother | NA |
| BKB | Some animals sleep on straw | 4 | animals | sleep | on | straw |
| BKB | The jam jar was full | 3 | jam | jar | full | NA |
| BKB | They’re kneeling down | 3 | Theyre | kneeling | down | NA |
| BKB | The girl lost her doll | 3 | girl | lost | doll | NA |
| BKB | The cooks making a cake | 3 | cooks | making | cake | NA |
| BKB | The child grabs the toy | 3 | child | grabs | toy | NA |
| BKB | The mud stuck on his shoe | 3 | mud | stuck | shoe | NA |
| BKB | The bath towel was wet | 3 | bath | towel | wet | NA |
| BKB | The matches lie on the shelf | 3 | matches | lie | shelf | NA |
| BKB | They’re running past the house | 4 | Theyre | running | past | house |
| BKB | The train had a bad crash | 3 | train | bad | crash | NA |
| BKB | The kitchen sink's empty | 3 | kitchen | sink | empty | NA |
| BKB | A boy fell from the window | 3 | boy | fell | window | NA |
| BKB | She used her spoon | 3 | She | used | spoon | NA |
| BKB | The park’s near the road | 3 | parks | near | road | NA |
| BKB | The cook cut some onions | 3 | cook | cut | onions | NA |
| BKB | The dog made an angry noise | 4 | dog | made | angry | noise |
| BKB | He’s washing his face | 3 | Hes | washing | face | NA |
| BKB | Somebody took the money | 3 | Somebody | took | money | NA |
| BKB | The light went out | 3 | light | went | out | NA |
| BKB | They wanted some potatoes | 3 | They | wanted | potatoes | NA |
| BKB | The naughty girl’s shouting | 3 | naughty | girls | shouting | NA |
| BKB | The cold milk's in a jug | 3 | cold | milks | jug | NA |
| BKB | The paint dripped on the ground | 3 | paint | dripped | ground | NA |
| BKB | The mother stirs the tea | 3 | mother | stirs | tea | NA |
| BKB | They laughed at his story | 3 | They | laughed | story | NA |
| BKB | Men wear long trousers | 4 | Men | wear | long | trousers |
| BKB | The small boy was asleep | 3 | small | boy | asleep | NA |
| BKB | The lady goes to the shop | 4 | lady | goes | to | shop |
| BKB | The sun melted the snow | 3 | sun | melted | snow | NA |
| BKB | The father's coming home | 3 | fathers | coming | home | NA |
| BKB | She had her pocket money | 3 | She | pocket | money | NA |
| BKB | The lorry drove up the road | 3 | lorry | drove | road | NA |
| BKB | He’s bringing his raincoat | 3 | Hes | bringing | raincoat | NA |
| BKB | A sharp knife's dangerous | 3 | sharp | knifes | dangerous | NA |
| BKB | They took some food | 3 | They | took | food | NA |
| BKB | The clever girls are reading | 3 | clever | girls | reading | NA |
| BKB | The broom stood in the corner | 3 | broom | stood | corner | NA |
| BKB | The woman tidied her house | 3 | woman | tidied | house | NA |
| BKB | The children dropped the bag | 3 | children | dropped | bag | NA |
| BKB | The dog came back | 3 | dog | came | back | NA |
| BKB | The floor looked clean | 3 | floor | looked | clean | NA |
| BKB | She found her purse | 3 | She | found | purse | NA |
| BKB | The fruit lies on the ground | 3 | fruit | lies | ground | NA |
| BKB | Mother fetches a saucepan | 3 | Mother | fetches | saucepan | NA |
| BKB | They washed in cold water | 4 | They | washed | cold | water |
| BKB | The young people are dancing | 3 | young | people | dancing | NA |
| BKB | The bus went early | 3 | bus | went | early | NA |
| BKB | They had two empty bottles | 4 | They | two | empty | bottles |
| BKB | A ball's bouncing along | 3 | balls | bouncing | along | NA |
| BKB | The father forgot the bread | 3 | father | forgot | bread | NA |
| BKB | The girl has a picture book | 3 | girl | picture | book | NA |
| BKB | The orange was quite sweet | 3 | orange | quite | sweet | NA |
| BKB | He’s holding his nose | 3 | Hes | holding | nose | NA |
| BKB | The new road’s on the map | 3 | new | roads | map | NA |
| BKB | The boy forgot his book | 3 | boy | forgot | book | NA |
| BKB | A friend came for lunch | 3 | friend | came | lunch | NA |
| BKB | The match boxes are empty | 3 | match | boxes | empty | NA |
| BKB | He climbed his ladder | 3 | He | climbed | ladder | NA |
| BKB | The family bought a house | 3 | family | bought | house | NA |
| BKB | The jug stood on the shelf | 3 | jug | stood | shelf | NA |
| BKB | The ball broke the window | 3 | ball | broke | window | NA |
| BKB | They’re shopping for cheese | 3 | Theyre | shopping | cheese | NA |
| BKB | The pond water's dirty | 3 | pond | waters | dirty | NA |
| BKB | They heard a funny noise | 4 | They | heard | funny | noise |
| BKB | Police are clearing the road | 3 | Police | clearing | road | NA |
| BKB | The bus stopped suddenly | 3 | bus | stopped | suddenly | NA |
| BKB | She writes to her brother | 3 | She | writes | brother | NA |
| BKB | The footballer lost a boot | 3 | footballer | lost | boot | NA |
| BKB | The three girls are listening | 3 | three | girls | listening | NA |
| BKB | The coat lies on a chair | 4 | coat | lies | on | chair |
| BKB | The book tells a story | 3 | book | tells | story | NA |
| BKB | The young boy left home | 4 | young | boy | left | home |
| BKB | They’re climbing the tree | 3 | Theyre | climbing | tree | NA |
| BKB | She stood near her window | 3 | She | stood | window | NA |
| BKB | The table has three legs | 3 | table | three | legs | NA |
| BKB | A letter fell on the mat | 3 | letter | fell | mat | NA |
| BKB | The five men are working | 3 | five | men | working | NA |
| BKB | He listens to his father | 4 | He | listens | to | father |
| BKB | The shoes were very dirty | 3 | shoes | very | dirty | NA |
| BKB | They went on holiday | 3 | They | went | holiday | NA |
| BKB | Baby broke his mug | 3 | Baby | broke | mug | NA |
| BKB | The lady packed her bag | 3 | lady | packed | bag | NA |
| BKB | The dinner plate's hot | 3 | dinner | plates | hot | NA |
| BKB | The train’s moving fast | 3 | trains | moving | fast | NA |
| BKB | The child drank some milk | 3 | child | drank | milk | NA |
| BKB | The car hit a wall | 3 | car | hit | wall | NA |
| BKB | A tea towel’s by the sink | 3 | tea | towels | sink | NA |
| BKB | The cleaner used a broom | 3 | cleaner | used | broom | NA |
| BKB | She looked in her mirror | 4 | She | looked | in | mirror |
| BKB | The good boy's helping | 3 | good | boys | helping | NA |
| BKB | They followed the path | 3 | They | followed | path | NA |
| BKB | The kitchen clock was wrong | 3 | kitchen | clock | wrong | NA |
| BKB | The dog jumped on the chair | 4 | dog | jumped | on | chair |
| BKB | Someone’s crossing the road | 3 | Someones | crossing | road | NA |
| BKB | The postman brings a letter | 3 | postman | brings | letter | NA |
| BKB | They’re cycling along | 3 | Theyre | cycling | along | NA |
| BKB | He broke his leg | 3 | He | broke | leg | NA |
| BKB | The milk was by the front door | 3 | milk | front | door | NA |
| BKB | The shirts hang in the cupboard | 3 | shirts | hang | cupboard | NA |
| BKB | The ground was too hard | 3 | ground | too | hard | NA |
| BKB | The buckets hold water | 3 | buckets | hold | water | NA |
| BKB | The chicken laid some eggs | 3 | chicken | laid | eggs | NA |
| BKB | The sweet shop was empty | 3 | sweet | shop | empty | NA |
| BKB | The dogs go for a walk | 3 | dogs | go | walk | NA |
| BKB | She’s washing her dress | 3 | Shes | washing | dress | NA |
| BKB | The lady stayed for tea | 3 | lady | stayed | tea | NA |
| BKB | The driver waits by the corner | 3 | driver | waits | corner | NA |
| BKB | They finished the dinner | 3 | They | finished | dinner | NA |
| BKB | The policeman knows the way | 3 | policeman | knows | way | NA |
| BKB | The little girl was happy | 3 | little | girl | happy | NA |
| BKB | He wore his yellow shirt | 4 | He | wore | yellow | shirt |
| BKB | They’re coming for Christmas | 3 | Theyre | coming | Christmas | NA |
| BKB | The cow gave some milk | 3 | cow | gave | milk | NA |
| BKB | The boy got into bed | 4 | boy | got | into | bed |
| BKB | The two farmers are talking | 3 | two | farmers | talking | NA |
| BKB | Mother picked some flowers | 3 | Mother | picked | flowers | NA |
| BKB | A fish lay on the plate | 3 | fish | lay | plate | NA |
| BKB | The father writes a letter | 3 | father | writes | letter | NA |
| BKB | The food cost a lot | 3 | food | cost | lot | NA |
| BKB | The girl's washing her hair | 3 | girls | washing | hair | NA |
| BKB | The front garden was pretty | 3 | front | garden | pretty | NA |
| BKB | He lost his hat | 3 | He | lost | hat | NA |
| BKB | The taps are above the sink | 3 | taps | above | sink | NA |
| BKB | Father paid at the gate | 4 | Father | paid | at | gate |
| BKB | She’s waiting for her bus | 3 | Shes | waiting | bus | NA |
| BKB | The bread van's coming | 3 | bread | vans | coming | NA |
| BKB | They had some cold meat | 3 | They | cold | meat | NA |
| BKB | The football game's over | 3 | football | games | over | NA |
| BKB | They carry some shopping bags | 4 | They | carry | shopping | bags |
| BKB | The children help the milkman | 3 | children | help | milkman | NA |
| BKB | The picture came from a book | 3 | picture | came | book | NA |
| BKB | The rice pudding was ready | 3 | rice | pudding | ready | NA |
| BKB | The boy had a toy dragon | 3 | boy | toy | dragon | NA |
| BKB | A tree fell on the house | 3 | tree | fell | house | NA |
| BKB | The fruit came in a box | 3 | fruit | came | box | NA |
| BKB | The husband brings some flowers | 3 | husband | brings | flowers | NA |
| BKB | They’re playing in the park | 3 | Theyre | playing | park | NA |
| BKB | She argued with her sister | 3 | She | argued | sister | NA |
| BKB | A man told the police | 3 | man | told | police | NA |
| BKB | Potatoes grow in the ground | 3 | otatoes | row | ground | NA |
| BKB | He’s cleaning his car | 3 | Hes | cleaning | car | NA |
| BKB | The mouse found the cheese | 3 | mouse | found | cheese | NA |
| BKB | They waited for one hour | 4 | They | waited | one | hour |
| BKB | The big dog was dangerous | 3 | big | dog | dangerous | NA |
| BKB | The strawberry jam was sweet | 3 | strawberry | jam | sweet | NA |
| BKB | The plant hangs above the door | 4 | plant | hangs | above | door |
| BKB | The children are all eating | 3 | children | all | eating | NA |
| BKB | The boy has black hair | 3 | boy | black | hair | NA |
| BKB | The mother heard her baby | 3 | mother | heard | baby | NA |
| BKB | The lorry climbed the hill | 3 | lorry | climbed | hill | NA |
| BKB | The angry man shouted | 3 | angry | man | shouted | NA |
| BKB | The dog sleeps in a basket | 3 | dog | sleeps | basket | NA |
| BKB | They’re drinking tea | 3 | Theyre | drinking | tea | NA |
| BKB | Mother opens the drawer | 3 | Mother | opens | drawer | NA |
| BKB | An old woman was at home | 3 | old | woman | home | NA |
| BKB | He dropped his money | 3 | He | dropped | money | NA |
| BKB | They broke all the eggs | 4 | They | broke | all | eggs |
| BKB | The kitchen window was clean | 3 | kitchen | window | clean | NA |
| BKB | The girl plays with the baby | 3 | girl | plays | baby | NA |
| BKB | The big fish got away | 4 | big | fish | got | away |
| BKB | She’s helping her friend | 3 | Shes | helping | friend | NA |
| BKB | The children washed the plates | 3 | children | washed | plates | NA |
| BKB | The postman comes early | 3 | postman | comes | early | NA |
| BKB | The sign showed the way | 3 | sign | showed | way | NA |
| BKB | The grass is getting long | 3 | grass | getting | long | NA |
| BKB | The match fell on the floor | 3 | match | fell | floor | NA |
| BKB | A man’s turning the tap | 3 | mans | turning | tap | NA |
| BKB | The fire was very hot | 3 | fire | very | hot | NA |
| BKB | He’s sucking his thumb | 3 | Hes | sucking | thumb | NA |
| BKB | The shop closed for lunch | 3 | shop | closed | lunch | NA |
| BKB | The driver starts the engine | 3 | driver | starts | engine | NA |
| BKB | The boy hurried to school | 3 | boy | hurried | school | NA |
| BKB | Some nice people are coming | 3 | nice | people | coming | NA |
| BKB | She bumped her head | 3 | She | bumped | head | NA |
| BKB | They met some friends | 4 | They | met | some | friends |
| BKB | Flowers grow in the garden | 3 | Flowers | grow | garden | NA |
| BKB | The tiny baby was pretty | 3 | tiny | baby | pretty | NA |
| BKB | The daughter laid the table | 3 | daughter | laid | table | NA |
| BKB | They walked across the grass | 4 | They | walked | across | grass |
| BKB | The mother tied the string | 3 | mother | tied | string | NA |
| BKB | The train stops at the station | 3 | train | stops | station | NA |
| BKB | The puppy plays with a ball | 3 | puppy | plays | ball | NA |
| BKB | The children wave at the train | 3 | children | wave | train | NA |
| BKB | Mother cut the Christmas cake | 4 | Mother | cut | Christmas | cake |
| BKB | He closed his eyes | 3 | He | closed | eyes | NA |
| BKB | The raincoat's very wet | 3 | raincoats | very | wet | NA |
| BKB | A lady buys some butter | 3 | lady | buys | butter | NA |
| BKB | They called an ambulance | 3 | They | called | ambulance | NA |
| BKB | She’s paying for her bread | 3 | Shes | paying | bread | NA |
| BKB | The policeman found a dog | 3 | policeman | found | dog | NA |
| BKB | Some men shave in the morning | 3 | men | shave | morning | NA |
| BKB | The driver lost his way | 3 | driver | lost | way | NA |
| BKB | They stared at the picture | 3 | They | stared | picture | NA |
| BKB | The cat drank from a saucer | 3 | cat | drank | saucer | NA |
| BKB | The oven door was open | 3 | oven | door | open | NA |
| BKB | The cars going too fast | 4 | cars | going | too | fast |
| BKB | The silly boy's hiding | 3 | silly | boys | hiding | NA |
| BKB | The painter used a brush | 3 | painter | used | brush | NA |
| BKB | The apple pie's cooking | 3 | apple | pies | cooking | NA |
| BKB | He drinks from his mug | 3 | He | drinks | mug | NA |
| BKB | The sky was very blue | 3 | sky | very | blue | NA |
| BKB | They knocked on the window | 3 | They | knocked | window | NA |
| BKB | The big boy kicked the ball | 4 | big | boy | kicked | ball |
| BKB | People are going home | 3 | People | going | home | NA |
| BKB | The baby wants his bottle | 3 | baby | wants | bottle | NA |
| BKB | The lady sat on her chair | 3 | lady | sat | chair | NA |
| BKB | They had some jam pudding | 3 | They | jam | pudding | NA |
| BKB | The scissors are quite sharp | 3 | scissors | quite | sharp | NA |
| BKB | She’s calling her daughter | 3 | Shes | calling | daughter | NA |
| BKB | Some brown leaves fell off the tree | 4 | brown | leaves | fell | tree |
| BKB | The milkman carried the cream | 3 | milkman | carried | cream | NA |
| BKB | A girl ran along | 3 | girl | ran | along | NA |
| BKB | The mother reads a paper | 3 | mother | reads | paper | NA |
| BKB | The dog chased the cat | 3 | dog | chased | cat | NA |
| BKB | The cake shop's opening | 3 | cake | shops | opening | NA |
| BKB | They like orange marmalade | 4 | They | like | orange | marmalade |
| BKB | The mother shut the window | 3 | mother | shut | window | NA |
| BKB | He’s skating with his friend | 4 | Hes | skating | with | friend |
| BKB | The cheese pie was good | 3 | cheese | pie | good | NA |
| BKB | Rain falls from clouds | 3 | Rain | falls | clouds | NA |
| BKB | She talked to her doll | 3 | She | talked | doll | NA |
| BKB | They painted the wall | 3 | They | painted | wall | NA |
| BKB | The towel dropped on the floor | 3 | towel | dropped | floor | NA |
| BKB | The dog's eating some meat | 3 | dogs | eating | meat | NA |
| BKB | A boy broke the fence | 3 | boy | broke | fence | NA |
| BKB | The yellow pears were lovely | 3 | yellow | pears | lovely | NA |
| BKB | The police help the driver | 3 | police | help | driver | NA |
| BKB | The snow lay on the roof | 3 | snow | lay | roof | NA |
| BKB | The lady washed the shirt | 3 | lady | washed | shirt | NA |
| BKB | The cup hangs on a hook | 3 | cup | hangs | hook | NA |
| BKB | The family like fish | 3 | family | like | fish | NA |
| BKB | Sugar's very sweet | 3 | Sugars | very | sweet | NA |
| BKB | The baby lay on a rug | 3 | baby | lay | rug | NA |
| BKB | The washing machine broke | 3 | washing | machine | broke | NA |
| BKB | They’re clearing the table | 3 | Theyre | clearing | table | NA |
| BKB | The cleaner swept the floor | 3 | cleaner | swept | floor | NA |
| BKB | A grocer sells butter | 3 | grocer | sells | butter | NA |
| BKB | The bath water was warm | 3 | bath | water | warm | NA |
| BKB | He’s reaching for his spoon | 3 | Hes | reaching | spoon | NA |
| BKB | She hurt her hand | 3 | She | hurt | hand | NA |
| BKB | The milkman drives a small van | 4 | milkman | drives | small | van |
| BKB | The boy slipped on the stairs | 4 | boy | slipped | on | stairs |
| BKB | They’re staying for supper | 3 | Theyre | staying | supper | NA |
| BKB | The girl held a mirror | 3 | girl | held | mirror | NA |
| BKB | The cup stood on a saucer | 3 | cup | stood | saucer | NA |
| BKB | The cows went to market | 3 | cows | went | market | NA |
| BKB | The boy got into trouble | 3 | boy | got | trouble | NA |
| BKB | They’re going out | 3 | Theyre | going | out | NA |
| BKB | The football hit the goalpost | 3 | football | hit | goalpost | NA |
| BKB | He paid his bill | 3 | He | paid | bill | NA |
| BKB | The teacloth's quite wet | 3 | teacloths | quite | wet | NA |
| BKB | A cat jumped off the fence | 4 | cat | jumped | off | fence |
| BKB | The baby has blue eyes | 3 | baby | blue | eyes | NA |
| BKB | They sat on a wooden bench | 4 | They | sat | wooden | bench |
| BKB | Mother made some curtains | 3 | Mother | made | curtains | NA |
| BKB | The oven's too hot | 3 | ovens | too | hot | NA |
| BKB | The girl caught a cold | 3 | girl | caught | cold | NA |
| BKB | The raincoat's hanging up | 3 | raincoats | hanging | up | NA |
| BKB | She brushed her hair | 3 | She | brushed | hair | NA |
| BKB | The two children are laughing | 3 | two | children | laughing | NA |
| BKB | The man tied his scarf | 3 | man | tied | scarf | NA |
| BKB | The flower stands in a pot | 3 | flower | stands | pot | NA |
| BKB | The pepper pot was empty | 3 | pepper | pot | empty | NA |
| BKB | The dog drank from a bowl | 3 | dog | drank | bowl | NA |
| BKB | A girl came into the room | 3 | girl | came | room | NA |
| BKB | They’re pushing an old car | 4 | Theyre | pushing | old | car |
| BKB | The cat caught a mouse | 3 | cat | caught | mouse | NA |
| BKB | The road goes up a hill | 4 | road | goes | up | hill |
| BKB | She made her bed | 3 | She | made | bed | NA |
| BKB | Bananas are yellow fruit | 3 | Bananas | yellow | fruit | NA |
| BKB | The cow lies on the grass | 3 | cow | lies | grass | NA |
| BKB | The egg cups are on the table | 3 | egg | cups | table | NA |
| BKB | He frightened his sister | 3 | He | frightened | sister | NA |
| BKB | The cricket team's playing | 3 | cricket | teams | playing | NA |
| BKB | The father picked some pears | 3 | father | picked | pears | NA |
| BKB | The kettle boiled quickly | 3 | kettle | boiled | quickly | NA |
| BKB | The man’s painting a sign | 3 | mans | painting | sign | NA |
| BKB | They lost some money | 3 | They | lost | money | NA |
| ASL | They moved the furniture. | 3 | They | moved | furniture | NA |
| ASL | He's wiping the table. | 3 | Hes | wiping | table | NA |
| ASL | He hit his head. | 3 | He | hit | head | NA |
| ASL | The yellow leaves are falling. | 3 | yellow | leaves | falling | NA |
| ASL | The cat played with some wool. | 3 | cat | played | wool | NA |
| ASL | The bag was very heavy. | 3 | bag | very | heavy | NA |
| ASL | The towel dripped on the carpet. | 3 | towel | dripped | carpet | NA |
| ASL | The bull chased the lady. | 3 | bull | chased | lady | NA |
| ASL | The man dug his garden. | 3 | man | dug | garden | NA |
| ASL | The room has a lovely view. | 3 | room | lovely | view | NA |
| ASL | The girl helped in the kitchen. | 3 | girl | helped | kitchen | NA |
| ASL | The old shoes were muddy. | 3 | old | shoes | muddy | NA |
| ASL | Father's hiding the presents. | 3 | Fathers | hiding | presents | NA |
| ASL | The milk boiled over. | 3 | milk | boiled | over | NA |
| ASL | The neighbour knocked at the door. | 3 | neighbour | knocked | door | NA |
| ASL | He tore his shirt. | 3 | He | tore | shirt | NA |
| ASL | They finished the jigsaw. | 3 | They | finished | jigsaw | NA |
| ASL | She brought her camera. | 3 | She | brought | camera | NA |
| ASL | The lady watered her plants. | 3 | lady | watered | plants | NA |
| ASL | The salt cellar's full. | 3 | salt | cellars | full | NA |
| ASL | The boy hit his thumb. | 3 | boy | hit | thumb | NA |
| ASL | The mother shook her head. | 3 | mother | shook | head | NA |
| ASL | The snow lay on the hills. | 3 | snow | lay | hills | NA |
| ASL | The father used a towel. | 3 | father | used | towel | NA |
| ASL | The tree was in the back garden. | 3 | tree | back | garden | NA |
| ASL | The yacht sailed past. | 3 | yacht | sailed | past | NA |
| ASL | The lady pushed the pram. | 3 | lady | pushed | pram | NA |
| ASL | They're leaving today. | 3 | Theyre | leaving | today | NA |
| ASL | The picture hung on the wall. | 3 | picture | hung | wall | NA |
| ASL | The children sit under the tree. | 3 | children | sit | tree | NA |
| ASL | The lunch was very early. | 3 | lunch | very | early | NA |
| ASL | The dirty boy is washing. | 3 | dirty | boy | washing | NA |
| ASL | He hid his money. | 3 | He | hid | money | NA |
| ASL | The curtains were too short. | 3 | curtains | too | short | NA |
| ASL | The knife cut the cake. | 3 | knife | cut | cake | NA |
| ASL | They emptied their pockets. | 3 | They | emptied | pockets | NA |
| ASL | The new shoes were tight. | 3 | new | shoes | tight | NA |
| ASL | The coat hangs in a cupboard. | 3 | coat | hangs | cupboard | NA |
| ASL | The sun shone through the clouds. | 3 | sun | shone | clouds | NA |
| ASL | She took her purse. | 3 | She | took | purse | NA |
| ASL | The team lost the match. | 3 | team | lost | match | NA |
| ASL | The shirt caught on a nail. | 3 | shirt | caught | nail | NA |
| ASL | They picked some raspberries. | 3 | They | picked | raspberries | NA |
| ASL | The man climbed the mountain. | 3 | man | climbed | mountain | NA |
| ASL | The lady hurt her arm. | 3 | lady | hurt | arm | NA |
| ASL | The old clothes were dirty. | 3 | old | clothes | dirty | NA |
| ASL | He carried a stick. | 3 | He | carried | stick | NA |
| ASL | She read her book. | 3 | She | read | book | NA |
| ASL | The new house was empty. | 3 | new | house | empty | NA |
| ASL | The thief brought a ladder. | 3 | thief | brought | ladder | NA |
| ASL | The horse stands by the gate. | 3 | horse | stands | gate | NA |
| ASL | They're heading for the park. | 3 | Theyre | heading | park | NA |
| ASL | The gardener trimmed the hedge. | 3 | gardener | trimmed | hedge | NA |
| ASL | They're standing up. | 3 | Theyre | standing | up | NA |
| ASL | Someone's hiding in the bushes. | 3 | Someones | hiding | bushes | NA |
| ASL | The waiter lit the candles. | 3 | waiter | lit | candles | NA |
| ASL | The baker iced the cake. | 3 | baker | iced | cake | NA |
| ASL | The woman slipped on the ice. | 3 | woman | slipped | ice | NA |
| ASL | The small puppy was scared. | 3 | small | puppy | scared | NA |
| ASL | The lady changed her mind. | 3 | lady | changed | mind | NA |
| ASL | The daughter closed the box. | 3 | daughter | closed | box | NA |
| ASL | He broke into the safe. | 3 | He | broke | safe | NA |
| ASL | The doctor carries a bag. | 3 | doctor | carries | bag | NA |
| ASL | The new game was silly. | 3 | new | game | silly | NA |
| ASL | The little boy was tired. | 3 | little | boy | tired | NA |
| ASL | They saw the sign. | 3 | They | saw | sign | NA |
| ASL | She's wrapping the parcel. | 3 | Shes | wrapping | parcel | NA |
| ASL | The children laughed at the clown. | 3 | children | laughed | clown | NA |
| ASL | The apple pie was hot. | 3 | apple | pie | hot | NA |
| ASL | The ship sailed up the river. | 3 | ship | sailed | river | NA |
| ASL | The house had a lovely garden. | 3 | house | lovely | garden | NA |
| ASL | The noisy dog is barking. | 3 | noisy | dog | barking | NA |
| ASL | They bought some tickets. | 3 | They | bought | tickets | NA |
| ASL | The man goes to the bank. | 3 | man | goes | bank | NA |
| ASL | The nurse helped the child. | 3 | nurse | helped | child | NA |
| ASL | The girl knew the story. | 3 | girl | knew | story | NA |
| ASL | He reached for a cup. | 3 | He | reached | cup | NA |
| ASL | The lady was quite cross. | 3 | lady | quite | cross | NA |
| ASL | The rope was too short. | 3 | rope | too | short | NA |
| ASL | She's listening to the radio. | 3 | Shes | listening | radio | NA |
| ASL | The husband cleaned the car. | 3 | husband | cleaned | car | NA |
| ASL | The postman leaned on the fence. | 3 | postman | leaned | fence | NA |
| ASL | The china vase was broken. | 3 | china | vase | broken | NA |
| ASL | The other team won. | 3 | other | team | won | NA |
| ASL | They locked the safe. | 3 | They | locked | safe | NA |
| ASL | The leaves dropped from the trees. | 3 | leaves | dropped | trees | NA |
| ASL | The men watched the race. | 3 | men | watched | race | NA |
| ASL | The bird's building a nest. | 3 | birds | building | nest | NA |
| ASL | The woman called her dog. | 3 | woman | called | dog | NA |
| ASL | They're waving at the train. | 3 | Theyre | waving | train | NA |
| ASL | The cat scratched the chair. | 3 | cat | scratched | chair | NA |
| ASL | She tapped at the window. | 3 | She | tapped | window | NA |
| ASL | The man painted the gate. | 3 | man | painted | gate | NA |
| ASL | He slid on the floor. | 3 | He | slid | floor | NA |
| ASL | They're lifting the box. | 3 | Theyre | lifting | box | NA |
| ASL | The woman listened to her iend. | 3 | woman | listened | iend | NA |
| ASL | The driver hooted his horn. | 3 | driver | hooted | horn | NA |
| ASL | The cake tasted nice. | 3 | cake | tasted | nice | NA |
| ASL | The sailor stood on the deck. | 3 | sailor | stood | deck | NA |
| ASL | The young girls were pretty. | 3 | young | girls | pretty | NA |
| ASL | They painted the ceiling. | 3 | They | painted | ceiling | NA |
| ASL | The back door was shut. | 3 | back | door | shut | NA |
| ASL | The tree lost its leaves. | 3 | tree | lost | leaves | NA |
| ASL | The boy eats with his fork. | 3 | boy | eats | fork | NA |
| ASL | The young mother's shopping. | 3 | young | mothers | shopping | NA |
| ASL | The girl sharpened her pencil. | 3 | girl | sharpened | pencil | NA |
| ASL | She closed her eyes. | 3 | She | closed | eyes | NA |
| ASL | The puppy licked his master. | 3 | puppy | licked | master | NA |
| ASL | The plant grows on the wall. | 3 | plant | grows | wall | NA |
| ASL | The family's having a picnic. | 3 | familys | having | picnic | NA |
| ASL | The train arrived on time. | 3 | train | arrived | time | NA |
| ASL | They won the game. | 3 | They | won | game | NA |
| ASL | The lady waited for her husband. | 3 | lady | waited | husband | NA |
| ASL | The post office was near. | 3 | post | office | near | NA |
| ASL | They rowed the boat. | 3 | They | rowed | boat | NA |
| ASL | The old fox was sly. | 3 | old | fox | sly | NA |
| ASL | The baby lost his rattle. | 3 | baby | lost | rattle | NA |
| ASL | He dug with his spade. | 3 | He | dug | spade | NA |
| ASL | The boiled egg was soft. | 3 | boiled | egg | soft | NA |
| ASL | The two ladies were watching. | 3 | two | ladies | watching | NA |
| ASL | The car engine's running. | 3 | car | engines | running | NA |
| ASL | They parked by the station. | 3 | They | parked | station | NA |
| ASL | The lemons were quite bitter. | 3 | lemons | quite | bitter | NA |
| ASL | They're cutting the grass. | 3 | Theyre | cutting | grass | NA |
| ASL | The woman called a doctor. | 3 | woman | called | doctor | NA |
| ASL | The man shaved with a razor. | 3 | man | shaved | razor | NA |
| ASL | He tied his shoelaces. | 3 | He | tied | shoelaces | NA |
| ASL | The bus is leaving early. | 3 | bus | leaving | early | NA |
| ASL | She's sewing on a button. | 3 | Shes | sewing | button | NA |
| ASL | The horse kicked the rider. | 3 | horse | kicked | rider | NA |
| ASL | The yellow bananas are ripe. | 3 | yellow | bananas | ripe | NA |
| ASL | The lady has a fur coat. | 3 | lady | fur | coat | NA |
| ASL | The cat jumped onto the table. | 3 | cat | jumped | table | NA |
| ASL | The book sits on the shelf. | 3 | book | sits | shelf | NA |
| ASL | The boy told a joke. | 3 | boy | told | joke | NA |
| ASL | She sings in the bath. | 3 | She | sings | bath | NA |
| ASL | The meat was too tough. | 3 | meat | too | tough | NA |
| ASL | The child ate some jam. | 3 | child | ate | jam | NA |
| ASL | They're stealing the apples. | 3 | Theyre | stealing | apples | NA |
| ASL | The children dried the dishes. | 3 | children | dried | dishes | NA |
| ASL | The paper boy was cheeky. | 3 | paper | boy | cheeky | NA |
| ASL | The little car was slow. | 3 | little | car | slow | NA |
| ASL | The bath taps are dripping. | 3 | bath | taps | dripping | NA |
| ASL | They came at Easter. | 3 | They | came | Easter | NA |
| ASL | He's wearing a tie. | 3 | Hes | wearing | tie | NA |
| ASL | The new towel was clean. | 3 | new | towel | clean | NA |
| ASL | The water poured from a jug. | 3 | water | poured | jug | NA |
| ASL | The red apples were in a bowl. | 3 | red | apples | bowl | NA |
| ASL | The bus stopped at the shops. | 3 | bus | stopped | shops | NA |
| ASL | The man drew with a pencil. | 3 | man | drew | pencil | NA |
| ASL | The lady cut her finger. | 3 | lady | cut | finger | NA |
| ASL | The horses stood under the tree. | 3 | horses | stood | tree | NA |
| ASL | Mother's talking to the milkman. | 3 | Mothers | talking | milkman | NA |
| ASL | She polished her shoes. | 3 | She | polished | shoes | NA |
| ASL | Some iends stayed for supper. | 3 | iends | stayed | supper | NA |
| ASL | The pudding was very good. | 3 | pudding | very | good | NA |
| ASL | The apples came in a bag. | 3 | apples | came | bag | NA |
| ASL | The greedy boy was hungry. | 3 | greedy | boy | hungry | NA |
| ASL | The three men were angry. | 3 | three | men | angry | NA |
| ASL | The children cleared the table. | 3 | children | cleared | table | NA |
| ASL | The man forgot his change. | 3 | man | forgot | change | NA |
| ASL | The clothes are covered in mud. | 3 | clothes | covered | mud | NA |
| ASL | The raincoat's wet through. | 3 | raincoats | wet | through | NA |
| ASL | The three iends are cycling. | 3 | three | iends | cycling | NA |
| ASL | They tied the rope. | 3 | They | tied | rope | NA |
| ASL | Christmas is coming soon. | 3 | Christmas | coming | soon | NA |
| ASL | The tall man was thin. | 3 | tall | man | thin | NA |
| ASL | The girl broke a vase. | 3 | girl | broke | vase | NA |
| ASL | The other team are losing. | 3 | other | team | losing | NA |
| ASL | The girl's playing tennis. | 3 | girls | playing | tennis | NA |
| ASL | The lady spoke to the driver. | 3 | lady | spoke | driver | NA |
| ASL | The noise scared the sheep. | 3 | noise | scared | sheep | NA |
| ASL | She's sitting on the swing. | 3 | Shes | sitting | swing | NA |
| ASL | The red ball's bouncing. | 3 | red | balls | bouncing | NA |
| ASL | The children heard the doorbell. | 3 | children | heard | doorbell | NA |
| ASL | They worked in the rain. | 3 | They | worked | rain | NA |
| ASL | The children carried the suitcase. | 3 | children | carried | suitcase | NA |
| ASL | The new teacher's nice. | 3 | new | teachers | nice | NA |
| ASL | The traffic lights are green. | 3 | traffic | lights | green | NA |
| ASL | They're going to the seaside. | 3 | Theyre | going | seaside | NA |
| ASL | The story's very exciting. | 3 | storys | very | exciting | NA |
| ASL | He's kicking the door. | 3 | Hes | kicking | door | NA |
| ASL | The pool was very deep. | 3 | pool | very | deep | NA |
| ASL | Mother served the soup. | 3 | Mother | served | soup | NA |
| ASL | The woman used her key. | 3 | woman | used | key | NA |
| ASL | The red dress was pretty. | 3 | red | dress | pretty | NA |
| ASL | The pears were too hard. | 3 | pears | too | hard | NA |
| ASL | He turned on the taps. | 3 | He | turned | taps | NA |
| ASL | She tore her dress. | 3 | She | tore | dress | NA |
| ASL | Mother's filling the kettle. | 3 | Mothers | filling | kettle | NA |
| ASL | The lady writes to her sister. | 3 | lady | writes | sister | NA |
| ASL | They're looking at the clock. | 3 | Theyre | looking | clock | NA |
| ASL | The farmer's buying some pigs. | 3 | farmers | buying | pigs | NA |
| ASL | The old man is leaving. | 3 | old | man | leaving | NA |
| ASL | The boy ate his lunch. | 3 | boy | ate | lunch | NA |
| ASL | The chocolate box was empty. | 3 | chocolate | box | empty | NA |
| ASL | The boy filled the buckets. | 3 | boy | filled | buckets | NA |
| ASL | The lorry drove up the hill. | 3 | lorry | drove | hill | NA |
| ASL | They called the police. | 3 | They | called | police | NA |
| ASL | The lady wore her coat. | 3 | lady | wore | coat | NA |
| ASL | The policeman stopped the traffic. | 3 | policeman | stopped | traffic | NA |
| ASL | The dog heard a noise. | 3 | dog | heard | noise | NA |
| ASL | The rose bush was blooming. | 3 | rose | bush | blooming | NA |
| ASL | The cows grazed in the field. | 3 | cows | grazed | field | NA |
| ASL | The sun came out. | 3 | sun | came | out | NA |
| ASL | He's starting the engine. | 3 | Hes | starting | engine | NA |
| ASL | He's visiting his uncle. | 3 | Hes | visiting | uncle | NA |
| ASL | The jam sticks to the plate. | 3 | jam | sticks | plate | NA |
| ASL | They're bringing some pears. | 3 | Theyre | bringing | pears | NA |
| ASL | The garden's very neat. | 3 | gardens | very | neat | NA |
| ASL | The red bus was late. | 3 | red | bus | late | NA |
| ASL | They're leaning on the ladder. | 3 | Theyre | leaning | ladder | NA |
| ASL | The ice cream is melting. | 3 | ice | cream | melting | NA |
| ASL | The green apples were sour. | 3 | green | apples | sour | NA |
| ASL | The family ate supper. | 3 | family | ate | supper | NA |
| ASL | The horse was quite old. | 3 | horse | quite | old | NA |
| ASL | The towel's quite dry. | 3 | towels | quite | dry | NA |
| ASL | The birds sang from the tree. | 3 | birds | sang | tree | NA |
| ASL | The lion escaped from the zoo. | 3 | lion | escaped | zoo | NA |
| ASL | The man took a picture. | 3 | man | took | picture | NA |
| ASL | They're buying some lunch. | 3 | Theyre | buying | lunch | NA |
| ASL | He's combing his hair. | 3 | Hes | combing | hair | NA |
| ASL | They watched the sunset. | 3 | They | watched | sunset | NA |
| ASL | Someone's listening at the door. | 3 | Someones | listening | door | NA |
| ASL | The girl told her mother. | 3 | girl | told | mother | NA |
| ASL | They did their homework. | 3 | They | did | homework | NA |
| ASL | The sister hurt her leg. | 3 | sister | hurt | leg | NA |
| ASL | The two boys are laughing. | 3 | two | boys | laughing | NA |
| ASL | The big needle was sharp. | 3 | big | needle | sharp | NA |
| ASL | The hill was very steep. | 3 | hill | very | steep | NA |
| ASL | Roses grow in the garden. | 3 | Roses | grow | garden | NA |
| ASL | The puppy chased the ball. | 3 | puppy | chased | ball | NA |
| ASL | The father answered the door. | 3 | father | answered | door | NA |
| ASL | The iends came for tea. | 3 | iends | came | tea | NA |
| ASL | The old man was poor. | 3 | old | man | poor | NA |
| ASL | They ate some plums. | 3 | They | ate | plums | NA |
| ASL | The mice saw the trap. | 3 | mice | saw | trap | NA |
| ASL | The car hit the tree. | 3 | car | hit | tree | NA |
| ASL | They're hanging up their coats. | 3 | Theyre | hanging | coats | NA |
| ASL | She's holding a brush. | 3 | Shes | holding | brush | NA |
| ASL | The man climbed the ladder. | 3 | man | climbed | ladder | NA |
| ASL | Mother cooked the dinner. | 3 | Mother | cooked | dinner | NA |
| ASL | The ice rink was closed. | 3 | ice | rink | closed | NA |
| ASL | The ice cream was cold. | 3 | ice | cream | cold | NA |
| ASL | They closed the curtains. | 3 | They | closed | curtains | NA |
| ASL | The doctor came quickly. | 3 | doctor | came | quickly | NA |
| ASL | The ice melted in the sun. | 3 | ice | melted | sun | NA |
| ASL | They ran up the hill. | 3 | They | ran | hill | NA |
| ASL | The milk was very cold. | 3 | milk | very | cold | NA |
| ASL | The father signed a letter. | 3 | father | signed | letter | NA |
| ASL | He remembered the way. | 3 | He | remembered | way | NA |
| ASL | The loud noise was sudden. | 3 | loud | noise | sudden | NA |
| ASL | The boy grazed his knee. | 3 | boy | grazed | knee | NA |
| ASL | The lady has small feet. | 3 | lady | small | feet | NA |
| ASL | They helped with the dishes. | 3 | They | helped | dishes | NA |
| ASL | They watched the cricket. | 3 | They | watched | cricket | NA |
| ASL | The girl carried a basket. | 3 | girl | carried | basket | NA |
| ASL | The baby sleeps in a cot. | 3 | baby | sleeps | cot | NA |
| ASL | The daughter drank some lemonade. | 3 | daughter | drank | lemonade | NA |
| ASL | The little girl was staring. | 3 | little | girl | staring | NA |
| ASL | They're living by the sea. | 3 | Theyre | living | sea | NA |
| ASL | The church stood on the hill. | 3 | church | stood | hill | NA |
| ASL | The farmer sowed some seeds. | 3 | farmer | sowed | seeds | NA |
| ASL | The east wind was cold. | 3 | east | wind | cold | NA |
| ASL | The blue towel was damp. | 3 | blue | towel | damp | NA |
| ASL | The man forgot his hat. | 3 | man | forgot | hat | NA |
| ASL | Mother's reading a story. | 3 | Mothers | reading | story | NA |
| ASL | She ironed her skirt. | 3 | She | ironed | skirt | NA |
| ASL | The floor was quite slippery. | 3 | floor | quite | slippery | NA |
| ASL | The biscuit tin was empty. | 3 | biscuit | tin | empty | NA |

Table A2. Four hundred brain parcels defined by the Schaefer atlas (Schaefer et al., 2018) and their corresponding MNI coordinates.

| Index | Parcel Name | x | y | z |
| --- | --- | --- | --- | --- |
| 1 | 17Networks_LH_VisCent_ExStr_1 | -36 | -62 | -17 |
| 2 | 17Networks_LH_VisCent_ExStr_2 | -23 | -73 | -10 |
| 3 | 17Networks_LH_VisCent_ExStr_3 | -36 | -81 | -16 |
| 4 | 17Networks_LH_VisCent_ExStr_4 | -17 | -86 | -15 |
| 5 | 17Networks_LH_VisCent_ExStr_5 | -24 | -97 | -12 |
| 6 | 17Networks_LH_VisCent_ExStr_6 | -41 | -87 | -3 |
| 7 | 17Networks_LH_VisCent_Striate_1 | -7 | -98 | -7 |
| 8 | 17Networks_LH_VisCent_ExStr_7 | -46 | -73 | 6 |
| 9 | 17Networks_LH_VisCent_ExStr_8 | -24 | -96 | 6 |
| 10 | 17Networks_LH_VisCent_ExStr_9 | -39 | -84 | 14 |
| 11 | 17Networks_LH_VisCent_ExStr_10 | -11 | -97 | 17 |
| 12 | 17Networks_LH_VisCent_ExStr_11 | -25 | -85 | 21 |
| 13 | 17Networks_LH_VisPeri_ExStrInf_1 | -24 | -55 | -8 |
| 14 | 17Networks_LH_VisPeri_ExStrInf_2 | -12 | -62 | -5 |
| 15 | 17Networks_LH_VisPeri_ExStrInf_3 | -7 | -76 | -6 |
| 16 | 17Networks_LH_VisPeri_ExStrInf_4 | -13 | -43 | -5 |
| 17 | 17Networks_LH_VisPeri_ExStrInf_5 | -14 | -57 | 1 |
| 18 | 17Networks_LH_VisPeri_StriCal_1 | -5 | -88 | 2 |
| 19 | 17Networks_LH_VisPeri_StriCal_2 | -7 | -74 | 9 |
| 20 | 17Networks_LH_VisPeri_ExStrSup_1 | -19 | -65 | 7 |
| 21 | 17Networks_LH_VisPeri_ExStrSup_2 | -3 | -84 | 24 |
| 22 | 17Networks_LH_VisPeri_ExStrSup_3 | -12 | -71 | 20 |
| 23 | 17Networks_LH_VisPeri_ExStrSup_4 | -16 | -89 | 33 |
| 24 | 17Networks_LH_VisPeri_ExStrSup_5 | -12 | -81 | 36 |
| 25 | 17Networks_LH_SomMotA_1 | -8 | -15 | 47 |
| 26 | 17Networks_LH_SomMotA_2 | -41 | -14 | 47 |
| 27 | 17Networks_LH_SomMotA_3 | -49 | -17 | 54 |
| 28 | 17Networks_LH_SomMotA_4 | -48 | -29 | 58 |
| 29 | 17Networks_LH_SomMotA_5 | -39 | -25 | 53 |
| 30 | 17Networks_LH_SomMotA_6 | -9 | -38 | 54 |
| 31 | 17Networks_LH_SomMotA_7 | -4 | -25 | 56 |
| 32 | 17Networks_LH_SomMotA_8 | -4 | -9 | 59 |
| 33 | 17Networks_LH_SomMotA_9 | -36 | -19 | 65 |
| 34 | 17Networks_LH_SomMotA_10 | -32 | -29 | 63 |
| 35 | 17Networks_LH_SomMotA_11 | -30 | -38 | 65 |
| 36 | 17Networks_LH_SomMotA_12 | -23 | -11 | 65 |
| 37 | 17Networks_LH_SomMotA_13 | -19 | -24 | 67 |
| 38 | 17Networks_LH_SomMotA_14 | -9 | -42 | 70 |
| 39 | 17Networks_LH_SomMotA_15 | -4 | -25 | 69 |
| 40 | 17Networks_LH_SomMotA_16 | -14 | -11 | 73 |
| 41 | 17Networks_LH_SomMotA_17 | -19 | -31 | 69 |
| 42 | 17Networks_LH_SomMotA_18 | -19 | -40 | 72 |
| 43 | 17Networks_LH_SomMotA_19 | -12 | -27 | 73 |
| 44 | 17Networks_LH_SomMotB_Aud_1 | -50 | -9 | 0 |
| 45 | 17Networks_LH_SomMotB_Aud_2 | -56 | -22 | 8 |
| 46 | 17Networks_LH_SomMotB_Ins_1 | -36 | -24 | 10 |
| 47 | 17Networks_LH_SomMotB_S2_1 | -38 | -9 | 13 |
| 48 | 17Networks_LH_SomMotB_S2_2 | -36 | -26 | 19 |
| 49 | 17Networks_LH_SomMotB_Aud_3 | -59 | -37 | 16 |
| 50 | 17Networks_LH_SomMotB_Aud_4 | -41 | -35 | 14 |
| 51 | 17Networks_LH_SomMotB_S2_3 | -49 | -13 | 14 |
| 52 | 17Networks_LH_SomMotB_S2_4 | -60 | -2 | 11 |
| 53 | 17Networks_LH_SomMotB_S2_5 | -48 | -24 | 18 |
| 54 | 17Networks_LH_SomMotB_S2_6 | -61 | -18 | 19 |
| 55 | 17Networks_LH_SomMotB_Cent_1 | -59 | -2 | 23 |
| 56 | 17Networks_LH_SomMotB_Cent_2 | -63 | -10 | 31 |
| 57 | 17Networks_LH_SomMotB_Cent_3 | -53 | -9 | 30 |
| 58 | 17Networks_LH_SomMotB_Cent_4 | -51 | -7 | 43 |
| 59 | 17Networks_LH_SomMotB_Cent_5 | -48 | -16 | 40 |
| 60 | 17Networks_LH_DorsAttnA_TempOcc_1 | -45 | -42 | -21 |
| 61 | 17Networks_LH_DorsAttnA_TempOcc_2 | -33 | -42 | -21 |
| 62 | 17Networks_LH_DorsAttnA_TempOcc_3 | -49 | -56 | -15 |
| 63 | 17Networks_LH_DorsAttnA_TempOcc_4 | -45 | -70 | -8 |
| 64 | 17Networks_LH_DorsAttnA_ParOcc_1 | -48 | -65 | 15 |
| 65 | 17Networks_LH_DorsAttnA_ParOcc_2 | -32 | -84 | 27 |
| 66 | 17Networks_LH_DorsAttnA_SPL_1 | -26 | -70 | 31 |
| 67 | 17Networks_LH_DorsAttnA_SPL_2 | -21 | -79 | 45 |
| 68 | 17Networks_LH_DorsAttnA_SPL_3 | -23 | -65 | 46 |
| 69 | 17Networks_LH_DorsAttnA_SPL_4 | -29 | -58 | 50 |
| 70 | 17Networks_LH_DorsAttnA_SPL_5 | -36 | -52 | 56 |
| 71 | 17Networks_LH_DorsAttnA_SPL_6 | -15 | -71 | 57 |
| 72 | 17Networks_LH_DorsAttnA_SPL_7 | -29 | -61 | 62 |
| 73 | 17Networks_LH_DorsAttnB_PostC_1 | -61 | -23 | 33 |
| 74 | 17Networks_LH_DorsAttnB_PostC_2 | -55 | -20 | 41 |
| 75 | 17Networks_LH_DorsAttnB_PostC_3 | -55 | -32 | 45 |
| 76 | 17Networks_LH_DorsAttnB_PostC_4 | -46 | -29 | 44 |
| 77 | 17Networks_LH_DorsAttnB_PostC_5 | -39 | -37 | 49 |
| 78 | 17Networks_LH_DorsAttnB_PostC_6 | -30 | -46 | 63 |
| 79 | 17Networks_LH_DorsAttnB_PostC_7 | -7 | -59 | 63 |
| 80 | 17Networks_LH_DorsAttnB_PostC_8 | -20 | -57 | 66 |
| 81 | 17Networks_LH_DorsAttnB_PostC_9 | -13 | -50 | 72 |
| 82 | 17Networks_LH_DorsAttnB_FEF_1 | -40 | -3 | 51 |
| 83 | 17Networks_LH_DorsAttnB_FEF_2 | -25 | -1 | 55 |
| 84 | 17Networks_LH_DorsAttnB_FEF_3 | -30 | -8 | 52 |
| 85 | 17Networks_LH_DorsAttnB_PrCv_1 | -50 | 3 | 38 |
| 86 | 17Networks_LH_SalVentAttnA_ParOper_1 | -55 | -32 | 22 |
| 87 | 17Networks_LH_SalVentAttnA_ParOper_2 | -58 | -44 | 27 |
| 88 | 17Networks_LH_SalVentAttnA_ParOper_3 | -61 | -36 | 33 |
| 89 | 17Networks_LH_SalVentAttnA_Ins_1 | -39 | 2 | -4 |
| 90 | 17Networks_LH_SalVentAttnA_Ins_2 | -40 | -15 | -2 |
| 91 | 17Networks_LH_SalVentAttnA_Ins_3 | -33 | 19 | 8 |
| 92 | 17Networks_LH_SalVentAttnA_Ins_4 | -36 | 4 | 11 |
| 93 | 17Networks_LH_SalVentAttnA_FrOper_1 | -50 | 1 | 5 |
| 94 | 17Networks_LH_SalVentAttnA_FrOper_2 | -52 | 9 | 13 |
| 95 | 17Networks_LH_SalVentAttnA_ParMed_1 | -11 | -27 | 41 |
| 96 | 17Networks_LH_SalVentAttnA_ParMed_2 | -13 | -41 | 47 |
| 97 | 17Networks_LH_SalVentAttnA_ParMed_3 | -6 | -49 | 57 |
| 98 | 17Networks_LH_SalVentAttnA_FrMed_1 | -7 | 0 | 41 |
| 99 | 17Networks_LH_SalVentAttnA_FrMed_2 | -5 | 9 | 48 |
| 100 | 17Networks_LH_SalVentAttnA_FrMed_3 | -8 | -3 | 71 |
| 101 | 17Networks_LH_SalVentAttnB_PFCl_1 | -38 | 49 | 11 |
| 102 | 17Networks_LH_SalVentAttnB_PFCl_2 | -29 | 43 | 30 |
| 103 | 17Networks_LH_SalVentAttnB_PFCl_3 | -36 | 32 | 38 |
| 104 | 17Networks_LH_SalVentAttnB_Ins_1 | -34 | 16 | -8 |
| 105 | 17Networks_LH_SalVentAttnB_Ins_2 | -33 | 25 | -1 |
| 106 | 17Networks_LH_SalVentAttnB_Ins_3 | -43 | 12 | 2 |
| 107 | 17Networks_LH_SalVentAttnB_OFC_1 | -27 | 49 | -14 |
| 108 | 17Networks_LH_SalVentAttnB_PFCmp_1 | -6 | 22 | 31 |
| 109 | 17Networks_LH_LimbicB_OFC_1 | -12 | 25 | -21 |
| 110 | 17Networks_LH_LimbicB_OFC_2 | -24 | 23 | -20 |
| 111 | 17Networks_LH_LimbicB_OFC_3 | -10 | 47 | -21 |
| 112 | 17Networks_LH_LimbicB_OFC_4 | -4 | 23 | -19 |
| 113 | 17Networks_LH_LimbicB_OFC_5 | -15 | 65 | -8 |
| 114 | 17Networks_LH_LimbicA_TempPole_1 | -37 | -5 | -42 |
| 115 | 17Networks_LH_LimbicA_TempPole_2 | -25 | 6 | -39 |
| 116 | 17Networks_LH_LimbicA_TempPole_3 | -26 | -9 | -33 |
| 117 | 17Networks_LH_LimbicA_TempPole_4 | -54 | -21 | -31 |
| 118 | 17Networks_LH_LimbicA_TempPole_5 | -40 | -21 | -27 |
| 119 | 17Networks_LH_LimbicA_TempPole_6 | -32 | 12 | -29 |
| 120 | 17Networks_LH_LimbicA_TempPole_7 | -44 | 5 | -17 |
| 121 | 17Networks_LH_ContA_Temp_1 | -55 | -62 | -1 |
| 122 | 17Networks_LH_ContA_IPS_1 | -29 | -74 | 42 |
| 123 | 17Networks_LH_ContA_IPS_2 | -58 | -42 | 45 |
| 124 | 17Networks_LH_ContA_IPS_3 | -35 | -62 | 48 |
| 125 | 17Networks_LH_ContA_IPS_4 | -45 | -41 | 47 |
| 126 | 17Networks_LH_ContA_IPS_5 | -33 | -46 | 41 |
| 127 | 17Networks_LH_ContA_PFCd_1 | -21 | 5 | 65 |
| 128 | 17Networks_LH_ContA_PFClv_1 | -48 | 35 | 10 |
| 129 | 17Networks_LH_ContA_PFClv_2 | -42 | 38 | 22 |
| 130 | 17Networks_LH_ContA_PFCl_1 | -49 | 6 | 26 |
| 131 | 17Networks_LH_ContA_PFCl_2 | -45 | 20 | 27 |
| 132 | 17Networks_LH_ContA_PFCl_3 | -39 | 7 | 34 |
| 133 | 17Networks_LH_ContA_Cingm_1 | -3 | 5 | 29 |
| 134 | 17Networks_LH_ContB_Temp_1 | -60 | -36 | -18 |
| 135 | 17Networks_LH_ContB_Temp_2 | -60 | -49 | -10 |
| 136 | 17Networks_LH_ContB_IPL_1 | -49 | -60 | 47 |
| 137 | 17Networks_LH_ContB_IPL_2 | -53 | -50 | 45 |
| 138 | 17Networks_LH_ContB_IPL_3 | -42 | -52 | 49 |
| 139 | 17Networks_LH_ContB_PFCd_1 | -30 | 14 | 57 |
| 140 | 17Networks_LH_ContB_PFClv_1 | -42 | 49 | -6 |
| 141 | 17Networks_LH_ContB_PFClv_2 | -28 | 58 | -1 |
| 142 | 17Networks_LH_ContB_PFClv_3 | -28 | 57 | 13 |
| 143 | 17Networks_LH_ContB_PFCmp_1 | -4 | 28 | 47 |
| 144 | 17Networks_LH_ContC_pCun_1 | -10 | -70 | 32 |
| 145 | 17Networks_LH_ContC_pCun_2 | -9 | -77 | 45 |
| 146 | 17Networks_LH_ContC_pCun_3 | -5 | -64 | 52 |
| 147 | 17Networks_LH_ContC_Cingp_1 | -6 | -41 | 24 |
| 148 | 17Networks_LH_ContC_Cingp_2 | -4 | -22 | 29 |
| 149 | 17Networks_LH_DefaultA_IPL_1 | -47 | -64 | 31 |
| 150 | 17Networks_LH_DefaultA_IPL_2 | -41 | -72 | 43 |
| 151 | 17Networks_LH_DefaultA_PFCd_1 | -25 | 28 | 43 |
| 152 | 17Networks_LH_DefaultA_PFCd_2 | -18 | 36 | 48 |
| 153 | 17Networks_LH_DefaultA_PFCd_3 | -22 | 20 | 52 |
| 154 | 17Networks_LH_DefaultA_pCunPCC_1 | -4 | -53 | 20 |
| 155 | 17Networks_LH_DefaultA_pCunPCC_2 | -5 | -60 | 30 |
| 156 | 17Networks_LH_DefaultA_pCunPCC_3 | -7 | -44 | 32 |
| 157 | 17Networks_LH_DefaultA_pCunPCC_4 | -4 | -34 | 38 |
| 158 | 17Networks_LH_DefaultA_pCunPCC_5 | -3 | -15 | 37 |
| 159 | 17Networks_LH_DefaultA_pCunPCC_6 | -3 | -68 | 41 |
| 160 | 17Networks_LH_DefaultA_pCunPCC_7 | -7 | -51 | 43 |
| 161 | 17Networks_LH_DefaultA_PFCm_1 | -5 | 55 | -10 |
| 162 | 17Networks_LH_DefaultA_PFCm_2 | -6 | 35 | -9 |
| 163 | 17Networks_LH_DefaultA_PFCm_3 | -6 | 59 | 7 |
| 164 | 17Networks_LH_DefaultA_PFCm_4 | -6 | 45 | 6 |
| 165 | 17Networks_LH_DefaultA_PFCm_5 | -16 | 67 | 8 |
| 166 | 17Networks_LH_DefaultA_PFCm_6 | -5 | 34 | 21 |
| 167 | 17Networks_LH_DefaultB_Temp_1 | -44 | 13 | -34 |
| 168 | 17Networks_LH_DefaultB_Temp_2 | -54 | -2 | -30 |
| 169 | 17Networks_LH_DefaultB_Temp_3 | -62 | -18 | -21 |
| 170 | 17Networks_LH_DefaultB_Temp_4 | -57 | -9 | -14 |
| 171 | 17Networks_LH_DefaultB_Temp_5 | -61 | -35 | -3 |
| 172 | 17Networks_LH_DefaultB_Temp_6 | -52 | -22 | -6 |
| 173 | 17Networks_LH_DefaultB_IPL_1 | -45 | -58 | 21 |
| 174 | 17Networks_LH_DefaultB_IPL_2 | -57 | -55 | 30 |
| 175 | 17Networks_LH_DefaultB_PFCd_1 | -4 | 51 | 28 |
| 176 | 17Networks_LH_DefaultB_PFCd_2 | -14 | 58 | 31 |
| 177 | 17Networks_LH_DefaultB_PFCd_3 | -22 | 51 | 31 |
| 178 | 17Networks_LH_DefaultB_PFCd_4 | -8 | 43 | 51 |
| 179 | 17Networks_LH_DefaultB_PFCd_5 | -13 | 24 | 61 |
| 180 | 17Networks_LH_DefaultB_PFCd_6 | -6 | 10 | 65 |
| 181 | 17Networks_LH_DefaultB_PFCl_1 | -41 | 19 | 48 |
| 182 | 17Networks_LH_DefaultB_PFCl_2 | -42 | 7 | 48 |
| 183 | 17Networks_LH_DefaultB_PFCv_1 | -36 | 22 | -16 |
| 184 | 17Networks_LH_DefaultB_PFCv_2 | -36 | 37 | -13 |
| 185 | 17Networks_LH_DefaultB_PFCv_3 | -46 | 32 | -10 |
| 186 | 17Networks_LH_DefaultB_PFCv_4 | -48 | 28 | 0 |
| 187 | 17Networks_LH_DefaultB_PFCv_5 | -53 | 19 | 11 |
| 188 | 17Networks_LH_DefaultC_IPL_1 | -40 | -79 | 30 |
| 189 | 17Networks_LH_DefaultC_Rsp_1 | -13 | -49 | 4 |
| 190 | 17Networks_LH_DefaultC_Rsp_2 | -8 | -52 | 9 |
| 191 | 17Networks_LH_DefaultC_Rsp_3 | -13 | -61 | 19 |
| 192 | 17Networks_LH_DefaultC_PHC_1 | -21 | -21 | -26 |
| 193 | 17Networks_LH_DefaultC_PHC_2 | -30 | -33 | -18 |
| 194 | 17Networks_LH_DefaultC_PHC_3 | -18 | -37 | -12 |
| 195 | 17Networks_LH_TempPar_1 | -53 | 6 | -11 |
| 196 | 17Networks_LH_TempPar_2 | -61 | -13 | -3 |
| 197 | 17Networks_LH_TempPar_3 | -62 | -32 | 5 |
| 198 | 17Networks_LH_TempPar_4 | -52 | -43 | 5 |
| 199 | 17Networks_LH_TempPar_5 | -57 | -54 | 10 |
| 200 | 17Networks_LH_TempPar_6 | -59 | -49 | 16 |
| 201 | 17Networks_RH_VisCent_ExStr_1 | 36 | -53 | -17 |
| 202 | 17Networks_RH_VisCent_ExStr_2 | 37 | -73 | -16 |
| 203 | 17Networks_RH_VisCent_ExStr_3 | 23 | -74 | -11 |
| 204 | 17Networks_RH_VisCent_ExStr_4 | 20 | -88 | -13 |
| 205 | 17Networks_RH_VisCent_ExStr_5 | 42 | -84 | -12 |
| 206 | 17Networks_RH_VisCent_ExStr_6 | 25 | -97 | -10 |
| 207 | 17Networks_RH_VisCent_Striate_1 | 8 | -92 | -2 |
| 208 | 17Networks_RH_VisCent_ExStr_7 | 35 | -89 | 2 |
| 209 | 17Networks_RH_VisCent_ExStr_8 | 24 | -99 | 7 |
| 210 | 17Networks_RH_VisCent_ExStr_9 | 43 | -79 | 10 |
| 211 | 17Networks_RH_VisCent_ExStr_10 | 13 | -94 | 19 |
| 212 | 17Networks_RH_VisCent_ExStr_11 | 27 | -87 | 21 |
| 213 | 17Networks_RH_VisPeri_ExStrInf_1 | 26 | -52 | -9 |
| 214 | 17Networks_RH_VisPeri_ExStrInf_2 | 18 | -36 | -12 |
| 215 | 17Networks_RH_VisPeri_ExStrInf_3 | 9 | -72 | -5 |
| 216 | 17Networks_RH_VisPeri_ExStrInf_4 | 13 | -58 | -3 |
| 217 | 17Networks_RH_VisPeri_ExStrInf_5 | 18 | -45 | -3 |
| 218 | 17Networks_RH_VisPeri_StriCal_1 | 9 | -74 | 9 |
| 219 | 17Networks_RH_VisPeri_StriCal_2 | 22 | -59 | 6 |
| 220 | 17Networks_RH_VisPeri_ExStrSup_1 | 16 | -66 | 19 |
| 221 | 17Networks_RH_VisPeri_ExStrSup_2 | 5 | -80 | 24 |
| 222 | 17Networks_RH_VisPeri_ExStrSup_3 | 14 | -78 | 34 |
| 223 | 17Networks_RH_VisPeri_ExStrSup_4 | 16 | -87 | 36 |
| 224 | 17Networks_RH_SomMotA_1 | 54 | -17 | 40 |
| 225 | 17Networks_RH_SomMotA_2 | 52 | -13 | 49 |
| 226 | 17Networks_RH_SomMotA_3 | 44 | -10 | 49 |
| 227 | 17Networks_RH_SomMotA_4 | 49 | -26 | 56 |
| 228 | 17Networks_RH_SomMotA_5 | 7 | -10 | 51 |
| 229 | 17Networks_RH_SomMotA_6 | 43 | -21 | 54 |
| 230 | 17Networks_RH_SomMotA_7 | 37 | -20 | 64 |
| 231 | 17Networks_RH_SomMotA_8 | 32 | -34 | 63 |
| 232 | 17Networks_RH_SomMotA_9 | 31 | -41 | 64 |
| 233 | 17Networks_RH_SomMotA_10 | 34 | -27 | 61 |
| 234 | 17Networks_RH_SomMotA_11 | 4 | -25 | 58 |
| 235 | 17Networks_RH_SomMotA_12 | 29 | -11 | 65 |
| 236 | 17Networks_RH_SomMotA_13 | 9 | -40 | 68 |
| 237 | 17Networks_RH_SomMotA_14 | 21 | -24 | 67 |
| 238 | 17Networks_RH_SomMotA_15 | 22 | -29 | 68 |
| 239 | 17Networks_RH_SomMotA_16 | 22 | -35 | 71 |
| 240 | 17Networks_RH_SomMotA_17 | 17 | -6 | 69 |
| 241 | 17Networks_RH_SomMotA_18 | 5 | -22 | 72 |
| 242 | 17Networks_RH_SomMotA_19 | 17 | -18 | 73 |
| 243 | 17Networks_RH_SomMotA_20 | 13 | -33 | 76 |
| 244 | 17Networks_RH_SomMotB_Aud_1 | 53 | 3 | -6 |
| 245 | 17Networks_RH_SomMotB_Aud_2 | 53 | -14 | 6 |
| 246 | 17Networks_RH_SomMotB_Ins_1 | 39 | -19 | 5 |
| 247 | 17Networks_RH_SomMotB_S2_1 | 37 | -8 | 14 |
| 248 | 17Networks_RH_SomMotB_S2_2 | 35 | -21 | 14 |
| 249 | 17Networks_RH_SomMotB_Aud_3 | 60 | -24 | 11 |
| 250 | 17Networks_RH_SomMotB_S2_3 | 41 | -13 | 18 |
| 251 | 17Networks_RH_SomMotB_S2_4 | 41 | -29 | 18 |
| 252 | 17Networks_RH_SomMotB_S2_5 | 50 | -10 | 13 |
| 253 | 17Networks_RH_SomMotB_S2_6 | 59 | 1 | 10 |
| 254 | 17Networks_RH_SomMotB_S2_7 | 49 | -21 | 19 |
| 255 | 17Networks_RH_SomMotB_S2_8 | 62 | -12 | 15 |
| 256 | 17Networks_RH_SomMotB_Cent_1 | 61 | 6 | 30 |
| 257 | 17Networks_RH_SomMotB_Cent_2 | 60 | -5 | 27 |
| 258 | 17Networks_RH_SomMotB_Cent_3 | 52 | -6 | 37 |
| 259 | 17Networks_RH_DorsAttnA_TempOcc_1 | 34 | -37 | -23 |
| 260 | 17Networks_RH_DorsAttnA_TempOcc_2 | 50 | -49 | -18 |
| 261 | 17Networks_RH_DorsAttnA_TempOcc_3 | 50 | -64 | -9 |
| 262 | 17Networks_RH_DorsAttnA_ParOcc_1 | 48 | -66 | 4 |
| 263 | 17Networks_RH_DorsAttnA_ParOcc_2 | 54 | -56 | 12 |
| 264 | 17Networks_RH_DorsAttnA_ParOcc_3 | 36 | -79 | 24 |
| 265 | 17Networks_RH_DorsAttnA_SPL_1 | 29 | -78 | 37 |
| 266 | 17Networks_RH_DorsAttnA_SPL_2 | 32 | -66 | 35 |
| 267 | 17Networks_RH_DorsAttnA_SPL_3 | 19 | -79 | 50 |
| 268 | 17Networks_RH_DorsAttnA_SPL_4 | 31 | -64 | 53 |
| 269 | 17Networks_RH_DorsAttnA_SPL_5 | 21 | -69 | 53 |
| 270 | 17Networks_RH_DorsAttnA_SPL_6 | 34 | -50 | 54 |
| 271 | 17Networks_RH_DorsAttnA_SPL_7 | 27 | -58 | 61 |
| 272 | 17Networks_RH_DorsAttnA_SPL_8 | 14 | -64 | 65 |
| 273 | 17Networks_RH_DorsAttnB_TempOcc_1 | 59 | -55 | -2 |
| 274 | 17Networks_RH_DorsAttnB_PostC_1 | 61 | -14 | 30 |
| 275 | 17Networks_RH_DorsAttnB_PostC_2 | 57 | -23 | 44 |
| 276 | 17Networks_RH_DorsAttnB_PostC_3 | 44 | -37 | 50 |
| 277 | 17Networks_RH_DorsAttnB_PostC_4 | 45 | -28 | 42 |
| 278 | 17Networks_RH_DorsAttnB_PostC_5 | 35 | -36 | 51 |
| 279 | 17Networks_RH_DorsAttnB_PostC_6 | 7 | -54 | 59 |
| 280 | 17Networks_RH_DorsAttnB_PostC_7 | 24 | -50 | 68 |
| 281 | 17Networks_RH_DorsAttnB_PostC_8 | 16 | -47 | 74 |
| 282 | 17Networks_RH_DorsAttnB_FEF_1 | 39 | -3 | 53 |
| 283 | 17Networks_RH_DorsAttnB_FEF_2 | 27 | -3 | 52 |
| 284 | 17Networks_RH_DorsAttnB_FEF_3 | 25 | -3 | 64 |
| 285 | 17Networks_RH_SalVentAttnA_ParOper_1 | 58 | -31 | 24 |
| 286 | 17Networks_RH_SalVentAttnA_ParOper_2 | 60 | -22 | 22 |
| 287 | 17Networks_RH_SalVentAttnA_ParOper_3 | 63 | -26 | 38 |
| 288 | 17Networks_RH_SalVentAttnA_PrC_1 | 51 | 3 | 41 |
| 289 | 17Networks_RH_SalVentAttnA_Ins_1 | 40 | 5 | -15 |
| 290 | 17Networks_RH_SalVentAttnA_Ins_2 | 41 | 8 | -3 |
| 291 | 17Networks_RH_SalVentAttnA_Ins_3 | 40 | -10 | -4 |
| 292 | 17Networks_RH_SalVentAttnA_Ins_4 | 39 | -2 | 6 |
| 293 | 17Networks_RH_SalVentAttnA_FrOper_1 | 38 | 7 | 11 |
| 294 | 17Networks_RH_SalVentAttnA_FrOper_2 | 49 | 5 | 3 |
| 295 | 17Networks_RH_SalVentAttnA_FrOper_3 | 54 | 12 | 12 |
| 296 | 17Networks_RH_SalVentAttnA_FrMed_1 | 7 | 2 | 43 |
| 297 | 17Networks_RH_SalVentAttnA_ParMed_1 | 11 | -17 | 41 |
| 298 | 17Networks_RH_SalVentAttnA_ParMed_2 | 12 | -34 | 43 |
| 299 | 17Networks_RH_SalVentAttnA_FrMed_2 | 6 | 11 | 58 |
| 300 | 17Networks_RH_SalVentAttnA_ParMed_3 | 10 | -43 | 53 |
| 301 | 17Networks_RH_SalVentAttnA_ParMed_4 | 11 | -32 | 50 |
| 302 | 17Networks_RH_SalVentAttnA_FrMed_3 | 7 | -2 | 67 |
| 303 | 17Networks_RH_SalVentAttnA_FrMed_4 | 16 | 7 | 69 |
| 304 | 17Networks_RH_SalVentAttnB_IPL_1 | 62 | -37 | 37 |
| 305 | 17Networks_RH_SalVentAttnB_PFClv_1 | 49 | 40 | 5 |
| 306 | 17Networks_RH_SalVentAttnB_PFCl_1 | 42 | 46 | 14 |
| 307 | 17Networks_RH_SalVentAttnB_PFCl_2 | 25 | 54 | 25 |
| 308 | 17Networks_RH_SalVentAttnB_PFCl_3 | 33 | 45 | 28 |
| 309 | 17Networks_RH_SalVentAttnB_Ins_1 | 34 | 21 | -8 |
| 310 | 17Networks_RH_SalVentAttnB_Ins_2 | 37 | 23 | 5 |
| 311 | 17Networks_RH_SalVentAttnB_PFCmp_1 | 8 | 35 | 25 |
| 312 | 17Networks_RH_SalVentAttnB_PFCmp_2 | 7 | 19 | 35 |
| 313 | 17Networks_RH_LimbicB_OFC_1 | 13 | 24 | -21 |
| 314 | 17Networks_RH_LimbicB_OFC_2 | 23 | 22 | -21 |
| 315 | 17Networks_RH_LimbicB_OFC_3 | 8 | 47 | -23 |
| 316 | 17Networks_RH_LimbicB_OFC_4 | 20 | 43 | -18 |
| 317 | 17Networks_RH_LimbicB_OFC_5 | 5 | 22 | -21 |
| 318 | 17Networks_RH_LimbicB_OFC_6 | 9 | 63 | -14 |
| 319 | 17Networks_RH_LimbicA_TempPole_1 | 28 | -1 | -40 |
| 320 | 17Networks_RH_LimbicA_TempPole_2 | 49 | -7 | -39 |
| 321 | 17Networks_RH_LimbicA_TempPole_3 | 37 | 17 | -38 |
| 322 | 17Networks_RH_LimbicA_TempPole_4 | 39 | -15 | -31 |
| 323 | 17Networks_RH_LimbicA_TempPole_5 | 29 | 12 | -30 |
| 324 | 17Networks_RH_LimbicA_TempPole_6 | 50 | -28 | -26 |
| 325 | 17Networks_RH_ContA_IPS_1 | 35 | -71 | 47 |
| 326 | 17Networks_RH_ContA_IPS_2 | 54 | -33 | 51 |
| 327 | 17Networks_RH_ContA_IPS_3 | 47 | -44 | 46 |
| 328 | 17Networks_RH_ContA_IPS_4 | 36 | -44 | 45 |
| 329 | 17Networks_RH_ContA_PFCd_1 | 24 | 10 | 58 |
| 330 | 17Networks_RH_ContA_PFCl_1 | 50 | 30 | 18 |
| 331 | 17Networks_RH_ContA_PFCl_2 | 48 | 18 | 23 |
| 332 | 17Networks_RH_ContA_PFCl_3 | 47 | 29 | 28 |
| 333 | 17Networks_RH_ContA_PFCl_4 | 49 | 8 | 25 |
| 334 | 17Networks_RH_ContA_PFCl_5 | 39 | 11 | 34 |
| 335 | 17Networks_RH_ContA_Cingm_1 | 5 | 1 | 30 |
| 336 | 17Networks_RH_ContB_Temp_1 | 62 | -28 | -20 |
| 337 | 17Networks_RH_ContB_Temp_2 | 63 | -42 | -11 |
| 338 | 17Networks_RH_ContB_IPL_1 | 55 | -45 | 33 |
| 339 | 17Networks_RH_ContB_IPL_2 | 54 | -53 | 44 |
| 340 | 17Networks_RH_ContB_IPL_3 | 56 | -41 | 48 |
| 341 | 17Networks_RH_ContB_IPL_4 | 41 | -55 | 48 |
| 342 | 17Networks_RH_ContB_PFCld_1 | 39 | 33 | 38 |
| 343 | 17Networks_RH_ContB_PFCld_2 | 45 | 19 | 44 |
| 344 | 17Networks_RH_ContB_PFCld_3 | 43 | 7 | 51 |
| 345 | 17Networks_RH_ContB_PFCld_4 | 34 | 15 | 56 |
| 346 | 17Networks_RH_ContB_PFClv_1 | 35 | 38 | -13 |
| 347 | 17Networks_RH_ContB_PFClv_2 | 28 | 55 | -14 |
| 348 | 17Networks_RH_ContB_PFClv_3 | 42 | 51 | -6 |
| 349 | 17Networks_RH_ContB_PFClv_4 | 27 | 59 | 3 |
| 350 | 17Networks_RH_ContB_PFCmp_1 | 5 | 28 | 48 |
| 351 | 17Networks_RH_ContC_pCun_1 | 17 | -63 | 28 |
| 352 | 17Networks_RH_ContC_pCun_2 | 13 | -71 | 39 |
| 353 | 17Networks_RH_ContC_pCun_3 | 5 | -64 | 44 |
| 354 | 17Networks_RH_ContC_pCun_4 | 7 | -50 | 45 |
| 355 | 17Networks_RH_ContC_pCun_5 | 8 | -71 | 53 |
| 356 | 17Networks_RH_ContC_Cingp_1 | 7 | -44 | 20 |
| 357 | 17Networks_RH_ContC_Cingp_2 | 6 | -26 | 28 |
| 358 | 17Networks_RH_DefaultA_Temp_1 | 61 | -8 | -23 |
| 359 | 17Networks_RH_DefaultA_IPL_1 | 53 | -53 | 26 |
| 360 | 17Networks_RH_DefaultA_IPL_2 | 47 | -64 | 42 |
| 361 | 17Networks_RH_DefaultA_PFCd_1 | 26 | 34 | 39 |
| 362 | 17Networks_RH_DefaultA_PFCd_2 | 24 | 26 | 51 |
| 363 | 17Networks_RH_DefaultA_pCunPCC_1 | 6 | -52 | 23 |
| 364 | 17Networks_RH_DefaultA_pCunPCC_2 | 5 | -63 | 31 |
| 365 | 17Networks_RH_DefaultA_pCunPCC_3 | 7 | -39 | 35 |
| 366 | 17Networks_RH_DefaultA_pCunPCC_4 | 4 | -20 | 37 |
| 367 | 17Networks_RH_DefaultA_pCunPCC_5 | 10 | -53 | 35 |
| 368 | 17Networks_RH_DefaultA_PFCm_1 | 5 | 41 | -11 |
| 369 | 17Networks_RH_DefaultA_PFCm_2 | 9 | 67 | 1 |
| 370 | 17Networks_RH_DefaultA_PFCm_3 | 7 | 42 | 4 |
| 371 | 17Networks_RH_DefaultA_PFCm_4 | 7 | 54 | 13 |
| 372 | 17Networks_RH_DefaultA_PFCm_5 | 17 | 65 | 16 |
| 373 | 17Networks_RH_DefaultA_PFCm_6 | 6 | 25 | 18 |
| 374 | 17Networks_RH_DefaultB_Temp_1 | 63 | -23 | -7 |
| 375 | 17Networks_RH_DefaultB_Temp_2 | 63 | -38 | 0 |
| 376 | 17Networks_RH_DefaultB_AntTemp_1 | 49 | 9 | -33 |
| 377 | 17Networks_RH_DefaultB_PFCd_1 | 6 | 58 | 29 |
| 378 | 17Networks_RH_DefaultB_PFCd_2 | 16 | 52 | 36 |
| 379 | 17Networks_RH_DefaultB_PFCd_3 | 5 | 44 | 40 |
| 380 | 17Networks_RH_DefaultB_PFCd_4 | 14 | 39 | 52 |
| 381 | 17Networks_RH_DefaultB_PFCd_5 | 12 | 20 | 63 |
| 382 | 17Networks_RH_DefaultB_PFCv_1 | 35 | 23 | -18 |
| 383 | 17Networks_RH_DefaultB_PFCv_2 | 48 | 32 | -8 |
| 384 | 17Networks_RH_DefaultB_PFCv_3 | 54 | 24 | 6 |
| 385 | 17Networks_RH_DefaultC_IPL_1 | 48 | -64 | 22 |
| 386 | 17Networks_RH_DefaultC_IPL_2 | 45 | -75 | 31 |
| 387 | 17Networks_RH_DefaultC_Rsp_1 | 14 | -46 | 4 |
| 388 | 17Networks_RH_DefaultC_Rsp_2 | 12 | -55 | 15 |
| 389 | 17Networks_RH_DefaultC_PHC_1 | 23 | -18 | -27 |
| 390 | 17Networks_RH_DefaultC_PHC_2 | 31 | -31 | -18 |
| 391 | 17Networks_RH_TempPar_1 | 47 | 16 | -20 |
| 392 | 17Networks_RH_TempPar_2 | 55 | -4 | -14 |
| 393 | 17Networks_RH_TempPar_3 | 49 | -20 | -8 |
| 394 | 17Networks_RH_TempPar_4 | 62 | -19 | 0 |
| 395 | 17Networks_RH_TempPar_5 | 50 | -33 | 2 |
| 396 | 17Networks_RH_TempPar_6 | 59 | -46 | 7 |
| 397 | 17Networks_RH_TempPar_7 | 51 | -41 | 13 |
| 398 | 17Networks_RH_TempPar_8 | 65 | -34 | 11 |
| 399 | 17Networks_RH_TempPar_9 | 55 | -46 | 19 |
| 400 | 17Networks_RH_TempPar_10 | 62 | -40 | 22 |

Note A3. A description of the XGBoost Learning algorithm implemented for the current study.

In a classification task, XGBoost used a set of functions (i.e., trees) to predict an output vector $Y$ (in this case, task conditions) from an input feature matrix $X$ (i.e., brain activations per participant) in a dataset $D$ (Chen & Guestrin, 2016). Here, $D=\left\{ \left( x_{i}, y_{i} \right) \right\}$ ($\left| D \right|=n$, $x_{i}\in\mathbb{R}^{m}$, $y_{i}\in\mathbb{R}^{n}$) had n samples and m features (also see *Model training and evaluation* below). Functions $f_{k}$ in a tree ensemble of $K$ trees were learnt by minimizing the output of the objective function $O$ (Equation 1):

$O=\sum_{i} l(\hat{y_{i}}, y_{i})+\sum_{k} \Omega(f_{k})$ $f$ $\in F$, (1)

In Equation 1, the loss function $l$ measured the distance between a prediction $\hat{y_{i}}$ and the corresponding true class $y$. The regularisation term $\Omega$ calculated the penalty for the complexity of the model. $F=\{f\left( x \right)=w_{q(x)}\}$ ($q: \mathbb{R}^{m}\to T, w\mathbb{R}^{T}$) represented the space of classification trees, where each $f_{k}$ corresponded to a tree structure $q$ and a set of leaf weights $w$. The model was trained on an additive manner, where the prediction of iteration $t$ was $\hat{y_{i}}= {\hat{y_{i}}}^{\left( t-1 \right)}+f_{t}(x)$. The objective function on the t-th iteration was updated as in Equation 2 with the tree function $f_{t}$ that minimized $O^{(t)}$:

$O^{(t)}=\sum_{i=1}^{n} l\left( y_{i}, {\hat{y_{i}}}^{\left( t-1 \right)}+f_{t}(x_{i}) \right)+\Omega(f_{t})$ (2)

Here, the objective function was approximated using Taylor expansion (Equation 3), where $g_{i}= \partial_{\hat{y}^{\left( t-1 \right)}}l(y_{i}, {\hat{y_{i}}}^{\left( t-1 \right)})$ and $h_{i}=\partial_{\hat{y}^{\left( t-1 \right)}}^{2}l(y_{i}, {\hat{y_{i}}}^{\left( t-1 \right)})$ were the first- and second-order partial derivatives with regard to the prediction ${\hat{y_{i}}}^{\left( t-1 \right)}$ in the last iteration $t-1$.

$O^{(t)}\simeq\sum_{i=1}^{n} l[\left( y_{i}, {\hat{y_{i}}}^{\left( t-1 \right)} \right)+g_{i}f_{t}\left( x_{i} \right)+\frac{1}{2}h_{i}f_{t}^{2}(x_{i})]+\Omega(f_{t})$ (3)

The derivative statistics $g_{i}$ and $h_{i}$ provided information about the direction to which the model parameters should be updated to reduce loss $l$. Specifically, the negative $g_{i}$ and $h_{i}$ were used to determine the optimal weight $w_{j}^{*}$ of leaf $j$ in a set of leaf instances $I$ (Equation 4) and the splits of the tree structure $q(X)$ (in a greedy algorithm, see Chen & Guestrin, 2016 for details). In Equation 4, $\lambda$ was derived from $\Omega(f_{t})$ to avoid overfitting. A learning rate $\epsilon$ was applied to $w_{j}^{*}$ by scaling all weights uniformly to give $\epsilon w_{j}^{*}$, which governed the pace at which $w_{j}^{*}$ was updated (also see *Model training and evaluation*).

$w_{j}^{*}=-\frac{\sum_{i\in I_{j}} g_{i}}{\lambda+\sum_{i\in I_{j}} h_{i}}$ (4)

The final predicted value $F_{T}^{\left( k \right)}(x)$ for each class k (e.g., Sp E Vis E) after $T$ iterations was passed through a softmax function to obtain the conditional probability $P$ of observing a class $k$ given the input $x$ (Equation 5). The class with the highest predicted probability was considered the predicted class for the input $x$.

$P\left( y=k \mid x \right)\mathbf{=}\frac{e^{F_{T}^{\left( k \right)}(x)}}{\sum_{i=1}^{K} e^{F_{T}^{\left( i \right)}(x)}}$ (5)

Note A4. The computational details of Shapley value (Shapley, 1953).

Shapley value evaluates individual feature’s contribution to model prediction, as defined in Equation 6.

$\phi_{i}=\sum_{S\subseteq\{x_{1},\ldots,x_{n}\}\backslash\{x_{p}\}} \frac{\left| S \right|!\left( N-\left| S \right|-1 \right)!}{N!}(f(S\cup\left\{ x_{i} \right\}-f\{S\}))$ (6)

Here, $\phi_{i}$represents the Shapley value of input feature $i$, $S$ is a subset of features, $x_{i}$ is the input vector of feature $i$, $N$ is the number of features, and $f(S)$ is the model’s prediction when considering only the features in subset $S$. The equation considers all possible subsets $S$ that do not contain feature $i$, and calculates the marginal contribution of feature $i$ by adding it to each subset $S$ and taking the difference in the model’s prediction. The weight $\frac{\left| S \right|!\left( N-\left| S \right|-1 \right)!}{N!}$ represents the probability of including feature $i$ in a subset $S$, considering all possible permutations of features.

# B. Supplementary model configurations and outputs

Table B1. Hyperparameters tested for the top 13-feature XGBoost model. Tested values denote the values tested in an extensive grid search. Best value denotes the values corresponding to the highest validation accuracy. See descriptions for hyperparameters in Table 1.

| Hyperparameters | Tested values | Best value |
| --- | --- | --- |
| colsample_bytree | 0.1, 0.3, 0.5, 0.7 | 0.7 |
| gamma | 0, 1, 2, 3, 4, 5, 6, 7 | 0 |
| learning_rate | 0.01, 0.04, 0.07, 0.1, 0.13 | 0.1 |
| max_depth | 1, 2, 3, 4, 5, 6 | 4 |
| n_estimators | 50, 100, 150, 200, 250, 300 | 150 |

Table B2. Model outputs for the repeated-measure ANOVA assessing main effects of Speech- and Visual-task difficulty, and their interaction on the post-scan sentence recall dprime scores.

|  | *Df*  *(between)* | *Df*  *(within)* | *MSE* | *F* | *ges* | *p* |
| --- | --- | --- | --- | --- | --- | --- |
| DiffSpeech | 1.0 | 23.0 | 0.085 | 6.830 | 0.041 | **0.016** |
| DiffVisual | 1.0 | 23.0 | 0.056 | 0.597 | 0.002 | 0.447 |
| DiffSpeech:DiffVisual | 1.0 | 23.0 | 0.061 | 0.611 | 0.003 | 0.442 |

Abbreviations: DiffSpeech: Speech-task difficulty. DiffVisual: Visual-task difficulty. Df (between): degrees of freedom between groups. Df (within): degrees of freedom within groups. MSE: mean squared error. Ges: generalised eta-squared.

Table B3. Hyperparameters tested for the pairwise binary classification XGBoost models with the top 13 features. Tested values denote the values tested in an extensive grid search. Pairwise classes show the classes included in the corresponding models. Best value denotes the values corresponding to the highest validation accuracy. See descriptions for hyperparameters in Table 1.

| Hyperparameters | Tested values | Pairwise classes | Best value |
| --- | --- | --- | --- |
| colsample_bytree | 0.1, 0.3, 0.5, 0.7 | Sp H Vis E (1) and Sp E Vis E (0) | 0.1 |
|  |  | Sp H Vis H (1) and Sp E Vis H (0) | 0.5 |
|  |  | Sp E Vis H (1) and Sp E Vis E (0) | 0.1 |
|  |  | Sp H Vis H (1) and Sp H Vis E (0) | 0.1 |
| gamma | 0, 1, 2, 3, 4, 5, 6, 7 | Sp H Vis E (1) and Sp E Vis E (0) | 1 |
|  |  | Sp H Vis H (1) and Sp E Vis H (0) | 1 |
|  |  | Sp E Vis H (1) and Sp E Vis E (0) | 0 |
|  |  | Sp H Vis H (1) and Sp H Vis E (0) | 0 |
| learning_rate | 0.01, 0.04, 0.07, 0.1, 0.13 | Sp H Vis E (1) and Sp E Vis E (0) | 0.01 |
|  |  | Sp H Vis H (1) and Sp E Vis H (0) | 0.13 |
|  |  | Sp E Vis H (1) and Sp E Vis E (0) | 0.1 |
|  |  | Sp H Vis H (1) and Sp H Vis E (0) | 0.1 |
| max_depth | 1, 2, 3, 4, 5, 6 | Sp H Vis E (1) and Sp E Vis E (0) | 2 |
|  |  | Sp H Vis H (1) and Sp E Vis H (0) | 2 |
|  |  | Sp E Vis H (1) and Sp E Vis E (0) | 2 |
|  |  | Sp H Vis H (1) and Sp H Vis E (0) | 4 |
| n_estimators | 50, 100, 150, 200, 250, 300 | Sp H Vis E (1) and Sp E Vis E (0) | 100 |
|  |  | Sp H Vis H (1) and Sp E Vis H (0) | 50 |
|  |  | Sp E Vis H (1) and Sp E Vis E (0) | 150 |
|  |  | Sp H Vis H (1) and Sp H Vis E (0) | 50 |

# C. Supplementary Figures


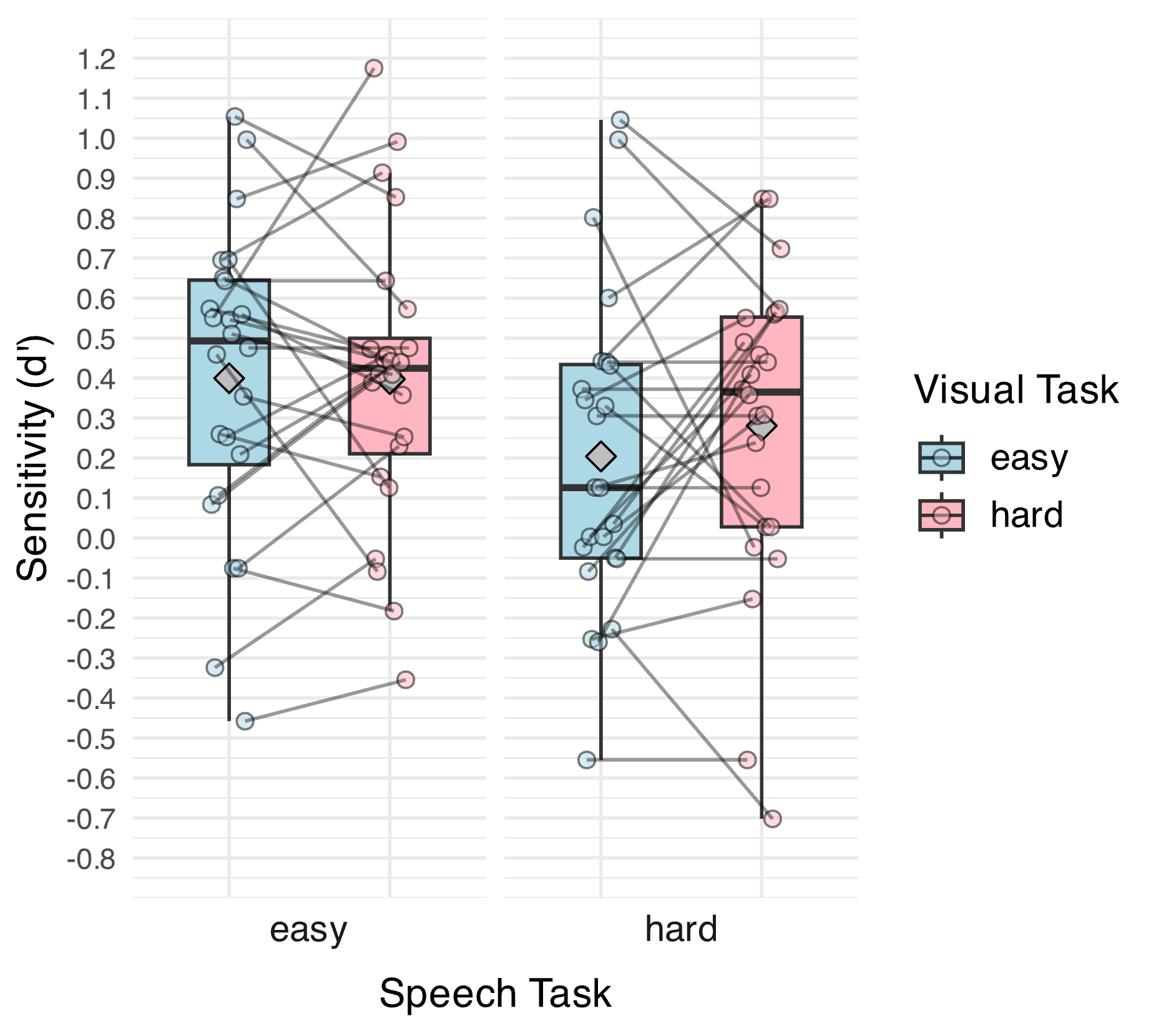


Figure C1. Sensitivity ($d'$) of detecting sentences used for the main runs from the unheard sentences. X-axis shows speech-task difficulty. Points display the mean response per participant and lines connect the individual responses across conditions. Grey diamonds denote the mean of each task condition.


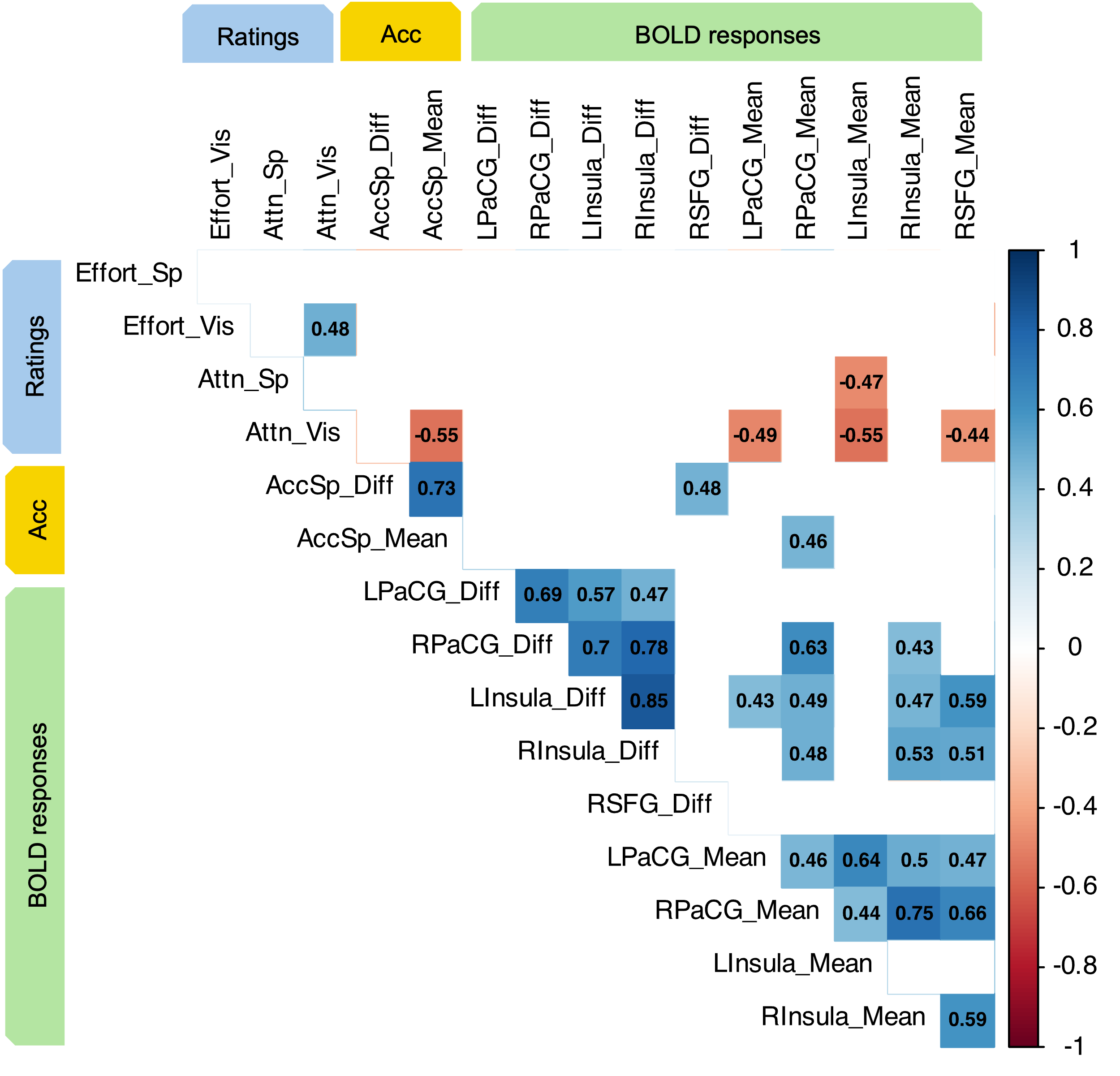


Figure C2. Figure legend continues on the next page.

Figure C2. An exploratory correlation matrix for the relationships between BOLD responses (i.e., GLM *β* estimates for the clusters showing a significant effect of speech hard > speech easy), scores for the effort and attention questionnaire, as well as task behavioural responses. Numbers show correlation coefficients. Positive correlations were displayed in blue and negative correlations in red. Blank tiles indicate non-significant correlations. The colour intensity of tiles reflects numeric value. Following Gennari et al. (2018), *p* values were not corrected for multiple correlations due to the nature of the exploratory analysis. Abbreviations: Effort_Sp: self-reported speech-task effort. Effort_Vis: self-reported visual-task effort. Attn_Sp: self-reported speech-task attention. Attn_Vis: self-reported visual-task attention. AccSp_Diff: difference in speech task response between hard and easy conditions. AccSp_Mean: mean speech-task response across all task conditions. LPaCG_Diff: difference in left PaCG response between hard and easy speech conditions. RPaCG_Diff: difference in right PaCG response between hard and easy speech conditions. LInsula_Diff: difference in left insula response between hard and easy speech conditions. RInsula_Diff: difference in right insula response between hard and easy speech conditions. LSFG_Diff: difference in left PaCG response between hard and easy speech conditions. LPaCG_Mean: mean left PaCG response across conditions. RPaCG_Mean: mean right PaCG response across conditions. LInsula_Mean: mean left insula response across conditions. RInsula_Mean: mean right insula response across conditions. RSFG_Mean: mean left PaCG response across conditions.


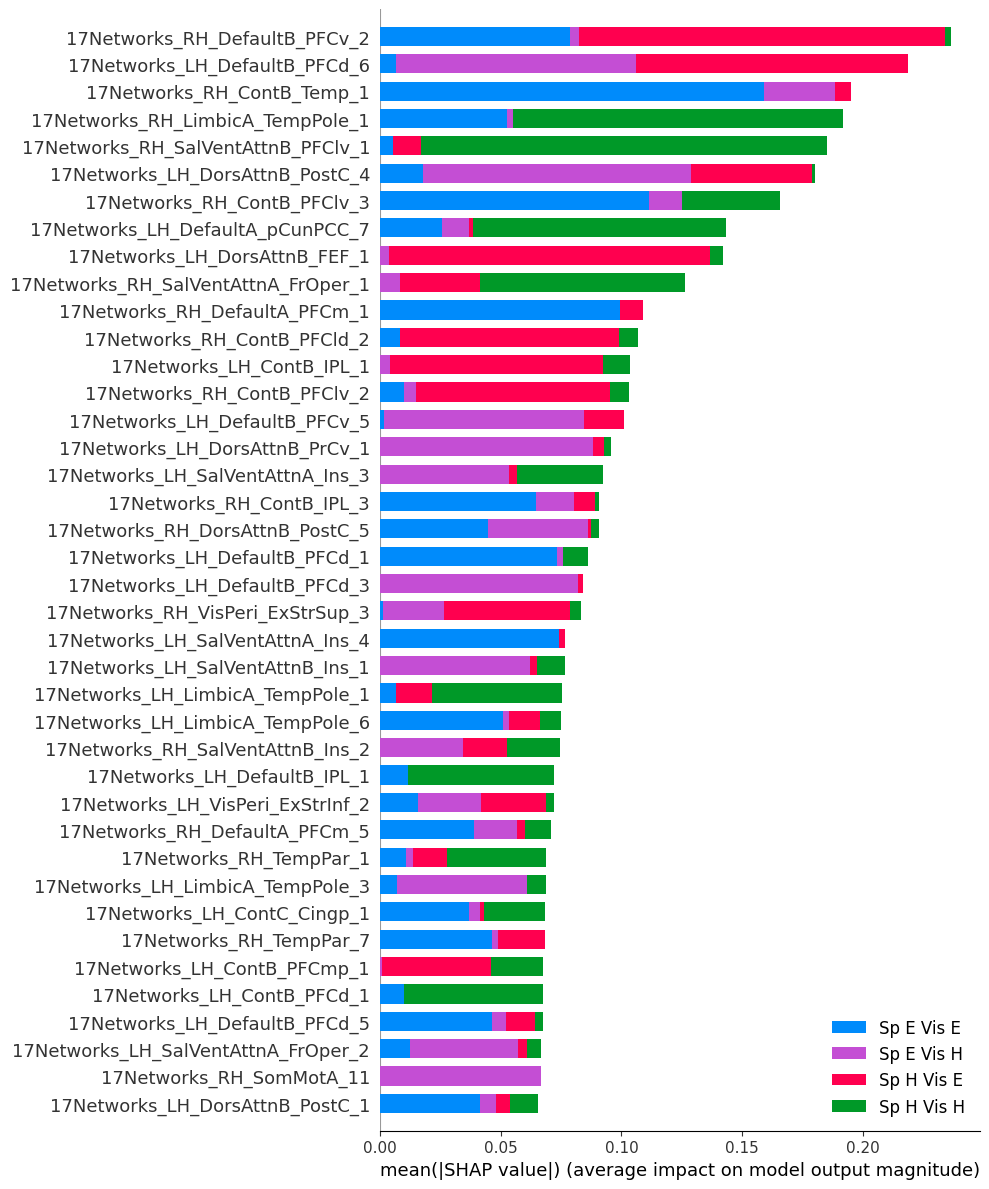


Figure C3. Figure legend continues on the next page.

Figure C3 (continued). Top 40 (out of 400) features ranked by their contribution (i.e., SHAP values) to classifying task conditions. Feature names follow the names of brain parcels defined by Schaefer et al. (2018). X axis displays the SHAP values averaged across all data points. Colour bars illustrate classes (i.e., task conditions), and the width of each bar represents the impact of a specific feature on differentiating a certain class from all other classes. See Table B12 for the corresponding MNI coordinates of these features. Abbreviations: Sp E Vis E: speech easy visual easy; Sp E Vis H: speech easy visual hard; Sp H Vis E: speech hard visual easy; Sp H Vis H: speech hard visual hard.

D. Questionnaires

Questionnaire D1

Effort and attention questionnaire

Those were all the trials in the main experiment phase. Thank you for your participation so far! We have a few short questions which take around 5 minutes to complete. Please make sure you write down the answer for all four questions, thanks!

**Q1**: Please indicate on a scale of 0-100 how **effortful** you found it to understand the **sentence**: /100

**Q2**: Please indicate on a scale of 0-100 how **effortful** you found it to decide the **angle of a patch**: /100

**Q3**: Please indicate on a scale of 0-100 how much **attention** you invested on understanding the **sentences**: /100

**Q4**: Please indicate on a scale of 0-100 how much **attention** you invested on deciding the **angle of a patch**: /100

**Q5**: Please leave any comments or remarks regarding the task procedure or unusual occurrences you may have below:
